# Discovery of novel DNA methylation biomarker panels for the diagnosis and differentiation between common adenocarcinomas and their liver metastases

**DOI:** 10.1038/s41598-024-53754-1

**Published:** 2024-02-07

**Authors:** Tina Draškovič, Nina Hauptman

**Affiliations:** Faculty of Medicine, Institute of Pathology, University of Ljubljana, Ljubljana, Slovenia

**Keywords:** Cancer, Cancer genetics, Epigenetics

## Abstract

Differentiation between adenocarcinomas is sometimes challenging. The promising avenue for discovering new biomarkers lies in bioinformatics using DNA methylation analysis. Utilizing a 2853-sample identification dataset and a 782-sample independent verification dataset, we have identified diagnostic DNA methylation biomarkers that are hypermethylated in cancer and differentiate between breast invasive carcinoma, cholangiocarcinoma, colorectal cancer, hepatocellular carcinoma, lung adenocarcinoma, pancreatic adenocarcinoma and stomach adenocarcinoma. The best panels for cancer type exhibit sensitivity of 77.8–95.9%, a specificity of 92.7–97.5% for tumors, a specificity of 91.5–97.7% for tumors and normal tissues and a diagnostic accuracy of 85.3–96.4%. We have shown that the results can be extended from the primary cancers to their liver metastases, as the best panels diagnose and differentiate between pancreatic adenocarcinoma liver metastases and breast invasive carcinoma liver metastases with a sensitivity and specificity of 83.3–100% and a diagnostic accuracy of 86.8–91.9%. Moreover, the panels could detect hypermethylation of selected regions in the cell-free DNA of patients with liver metastases. At the same time, these were unmethylated in the cell-free DNA of healthy donors, confirming their applicability for liquid biopsies.

## Introduction

Malignant liver tumors, which include primary liver tumors and liver metastases, are among the most common malignancies worldwide. Among primary malignant tumors, hepatocellular carcinoma is the most prevalent, followed by cholangiocarcinoma. Liver metastases include mostly carcinomas, the most common subtype being adenocarcinoma. The major sources of liver metastases are adenocarcinomas of the colon and rectum, followed by carcinomas of the pancreas, breast, lung and stomach. The disease has a poor prognosis and poor overall survival, especially with liver metastases^1–4^. Because of the variation in prognosis and treatment options, differentiation between malignant tumors of the liver and prediction of tumor origin in patients with carcinoma of unknown primary (CUP) in the liver are of vital importance. The correct diagnosis can be made using various methods; one of which is DNA methylation analysis.

DNA methylation plays an important role as an epigenetic mechanism of cancer initiation, maintenance and progression. Normally, knowledge of the methylation status of a single CpG site is of limited value unless it is related to the status of neighboring CpG sites. Therefore, efforts in the field of cancer epigenetics have primarily focused on the detection of differentially methylated regions (DMRs) comprising multiple consecutive methylated CpG sites. DMRs can occur throughout the genome but have been identified primarily in the promoter regions of genes, within the body of genes and in intergenic regulatory regions^5–8^. Clusters of hypermethylated CpG sites within a gene's promoter region are commonly associated with gene silencing, while coordinated hypermethylation in intragenic regions is associated with gene upregulation^5,7^. In cancer, hypermethylation contributes to the disease phenotype through genetic inactivation of tumor suppressor genes and DNA repair genes, thereby increasing the rate of mutagenesis. These genes are frequently associated with the cell cycle, apoptosis, cell division, DNA repair and DNA replication^8–10^.

DMRs and even single CpG sites with cancer-specific methylation changes have the potential to have clinical implications as diagnostic biomarkers. Epigenetic profiles inherently reflect tissue differentiation in normal and malignant tissues^11^. Alterations in methylation patterns are highly pervasive across a given tumor type. The unique methylation profile of cells can be used to distinguish cancer cells from healthy tissues and identify the tissue of origin of DNA^11,12^. In addition, a small number of selected CpG sites can be used to successfully differentiate between different cancer types^10^. Hypermethylation occurs early in cancer development and remains methylated across all stages of cancer progression^11,13,14^. Therefore, DNA methylation biomarkers can be used as potential diagnostic biomarkers to predict tumor origin in patient with metastatic cancer and CUP^10,12,15–17^. Additionally, DNA methylation markers can be extended from tissue samples to liquid biopsy, which is one of the most promising applications in the near future^3^.

Methylation array-based data from the Illumina Infinium HumanMethylation450 BeadChip (HM450) and lllumina MethylationEPIC BeadChip (EPIC) remain the most used platforms for genome wide methylation studies. They use hybridization of bisulfite-treated DNA with array probes in combination with single nucleotide extension, to measure methylation at the genomic hybridization site for a single CpG dinucleotide^18–21^. Because adjacent CpG sites are more likely to share the same methylation status, they often reflect the methylation status of a CpG-rich region^22^. Therefore, it is not surprising that the HM450 and EPIC arrays remain the most widely used assays for identifying DMRs to date. The chips enable simultaneous determination of the methylation status of more than 450,000/850,000 CpG sites in the human genome. To provide the most comprehensive overview of methylation status, they cover 96% of CpG islands, shelves, and shores, as well as gene regions^18–21^. The methylation values of the HM450 and EPIC arrays have been shown to be in excellent agreement with those of bisulfite sequencing, which further supports the credibility of those platforms^18,23,24^.

The aim of our research was to identify and verify novel cancer-specific methylation biomarkers that successfully differentiate between selected adenocarcinomas: breast invasive carcinoma (BRCA), cholangiocarcinoma (CHOL), colorectal cancer (CRC), hepatocellular carcinoma (LIHC), lung adenocarcinoma (LUAD), pancreatic adenocarcinoma (PAAD) and stomach adenocarcinoma (STAD). We used two different approaches for the identification dataset: a non-clustered approach (no sample clustering was performed) and a clustered approach (unsupervised sample clustering was performed within each project before probe selection). For each cancer type, we identified and verified multiple candidate methylation biomarkers in each approach and constructed panels that successfully differentiate between included adenocarcinoma and their liver metastases and can be detected in cell-free DNA (cfDNA). In addition, we were interested in the differences/similarities in the results of non-clustered and clustered approach.

## Results

The bioinformatic analysis was performed on primary tumor samples and normal tissue samples from the BRCA, CHOL, COAD (colon adenocarcinoma), LIHC, LUAD, PAAD, READ (rectum adenocarcinoma) and STAD projects from The Cancer Genome Atlas (TCGA) dataset. The COAD and READ projects were merged and further addressed as CRC. After data preparation, a total of 2609 primary tumor samples and 244 normal tissue samples of selected projects were included. The number of primary tumor samples and normal samples per project included in our study is shown in Supplementary Table S1. The methylation data used in our study were collected through experiment with the HM450, the most comprehensive collection of methylation data available at TCGA. The chip provides information on the methylation status of more than 450,000 CpG sites in the human genome, which formed the starting point for our selection. In our results, methylation levels are quantified by the average beta value, which ranges from zero (unmethylated) to one (fully methylated)^25^. The range of beta values between zero and one can be interpreted as an approximation of the percentage of methylation of a selected CpG site or CpG sites in the studied region in the sample^26^. Using our bioinformatics analysis, we narrowed down and selected the best probe candidates for each cancer type to differentiate between the included cancer types. We focused on probes that were hypermethylated in the cancer types of interest and unmethylated in the comparator types. The results from the TCGA dataset were verified using an independent Gene Expression Omnibus (GEO) dataset with 823 samples (418 primary tumor samples, 364 normal tissue samples, 31 liver metastasis samples and 9 cfDNA samples) (Supplementary Table S2).

### Clustering of methylation data

Unsupervised clustering was performed separately for each project in TCGA dataset. Each individual methylation cluster (MC) represent a group of samples within the project with similar methylation level, where calculated beta value represent the average methylation level of all HM450 probes in individual sample. Our analysis resulted in four to seven MCs per individual project. MC1 represent the group of samples within the project with the highest average methylation level, MC2 represent the group of samples with the second highest average methylation level, etc. The results of clustering analysis, the beta values and the number of tumor samples in each cluster are shown in Supplementary Table S1. DNA methylation heatmaps depicting the methylation pattern of the included adenocarcinomas are shown in Supplementary Fig. S1.

### Selected hypermethylated probes

#### Non-clustered approach

For each cancer type, our bioinformatic analysis revealed a set of probes and associated CpG sites in human genome that are hypermethylated in that cancer and hypomethylated in the compared cancer types. The non-clustered approach resulted in two to twenty-one probes per cancer type. All probes and beta values across all cancer types and groups of normal tissue samples and distribution of hypermethylated samples across projects for every probe can be found in the Supplementary Table S3 and Supplementary Table S4. The best probes from non-clustered approach are listed in Supplementary Table S5. The individual probes beta values of included tumor samples and normal tissue samples are shown in Supplementary Fig. S2.

#### Clustered approach

Using a clustered approach, bioinformatic analyses revealed a number of probes and associated CpG sites in the human genome that were hypermethylated in the MC of interest and hypomethylated in the compared cancer types. More specifically, it reveals a set of probes that are hypermethylated in a subset of the included tumor samples (in MC of interest) and does not necessarily show up in the majority of samples as in the non-clustered approach. The number of probes varied from zero to 74 probes per MC. The absence of hypermethylated probes was most frequently observed in MCs with the lowest average methylation levels. All probes and beta values across all cancer types and groups of normal tissue samples and distribution of hypermethylated samples across projects for every probe can be found in the Supplementary Table S6 and Supplementary Table S7. For the selected probes the sensitivity and specificity of each probe to differentiate between cancer and paired normal tissue samples was calculated. In addition, the sensitivity and specificity of the probes to differentiate between all cancer types (all primary tumor samples) and all included samples (all primary tumor samples and normal tissue samples) were calculated. The best probes from non-clustered approach are listed in Supplementary Table S8. The distribution of beta values of individual probes over all included tumor clusters and normal tissue samples is shown in Supplementary Fig. S3. Some of the probes obtained with the clustered approach were the same as those obtained with the non-clustered approach. The majority of these probes were significantly hypermethylated in multiple MCs.

### Panels

#### Hepatocellular carcinoma (LIHC)

**Figure 1:**
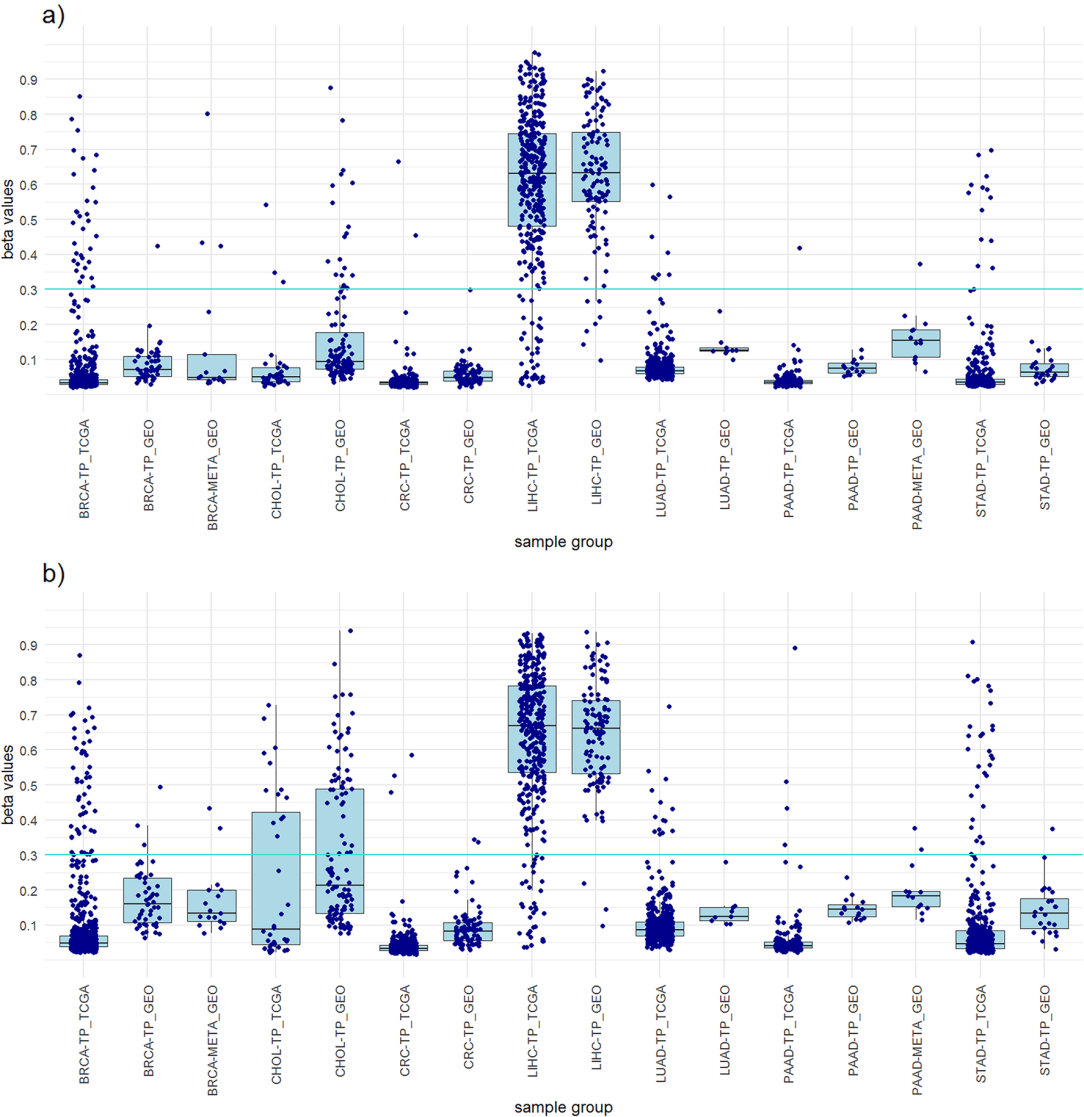
Boxplots showing the distribution of the highest beta values of all included samples from the LIHC panels of both approaches and a comparison between the TCGA dataset and the GEO dataset. (**a**) LIHC panel clustered approach. (**b**) LIHC panel non-clustered approach.

In TCGA dataset LIHC resulted in highest number of hypermethylated probes among all cancers. Six probes included in the LIHC panel from the non-clustered approach differentiate LIHC from other cancers with 91.2% sensitivity, 94.8% specificity for all tumor samples, 95.2% specificity for all samples and 94.7% diagnostic accuracy (Table 1). The panel successfully differentiate LIHC from normal liver tissue with a specificity of 100%. In the clustered approach, eight probes were selected for the panel design. The panel showed slightly lower sensitivity and specificity for LIHC, but higher specificities and diagnostic accuracy than the panel from non-clustered approach (Table 1). The probe cg18485193 was used in both panels (Supplementary Fig. S2). This probe was hypermethylated in five of six LIHC clusters (LIHC MC2–MC6). The results of the GEO dataset show a high degree of agreement with the results of the TCGA dataset, with even higher LIHC sensitivity and similar specificities (Table 1). This can be seen on Fig. 1, which shows the distribution of the highest beta values of all included samples from the panels of both approaches and a comparison between the TCGA dataset and the GEO dataset.

**Table 1:** LIHC panels from clustered and non-clustered approach.

LIHC panel	Selected probes	No. of probes	Primary cancer						All tumor samples			All samples			Statistics				
			No. TS β > 0.3	No. TS β < 0.3	No. all TS	No. NT β > 0.3	No. NT β < 0.3	No. all NT	No. other TS β > 0.3	No. other TS β < 0.3	No. all TS	No. other β > 0.3	No. other β < 0.3	No. all samples	LIHC Sensitivity	LIHC Specificity	TS Specificity	All samples Specificity	Accuracy
TCGA dataset																			
Non-clustered approach	cg13204512 cg18771357 cg18028711 cg26240185 cg16579555 cg18485193	6	344	33	377	0	50	50	116	2116	2609	119	2357	2853	91.2%	100.0%	94.8%	95.2%	94.7%
Clustered approach	cg18485193 cg10376598 cg04012924 cg01362570 cg05040544 cg18354742 cg20797142 cg24217704	8	336	41	377	1	49	50	61	2171	2609	62	2413	2853	89.1%	98.0%	97.3%	97.5%	96.4%
GEO dataset																			
Non-clustered approach	cg13204512 cg18771357 cg18028711 cg26240185 cg16579555 cg18485193	6	112	2	114	0	48	48	51	253	418	66	602	782	98.2%	100.0%	83.3%	90.1%	91.3%
Clustered approach	cg18485193 cg10376598 cg04012924 cg01362570 cg05040544 cg18354742 cg20797142 cg24217704	8	110	4	114	4	44	48	18	286	418	24	644	782	96.5%	91.7%	94.1%	96.4%	96.4%

LIHC sensitivity, LIHC specificity, all tumor samples specificity, all samples specificity and diagnostic accuracy was calculated.

LIHC, Liver hepatocellular carcinoma; No., Number of samples; TS, Tumor samples; NS, Normal tissues; β, Beta value; TCGA, The Cancer Genome Atlas; GEO, Gene Expression Omnibus.

#### Cholangiocarcinoma (CHOL)

**Figure 2:**
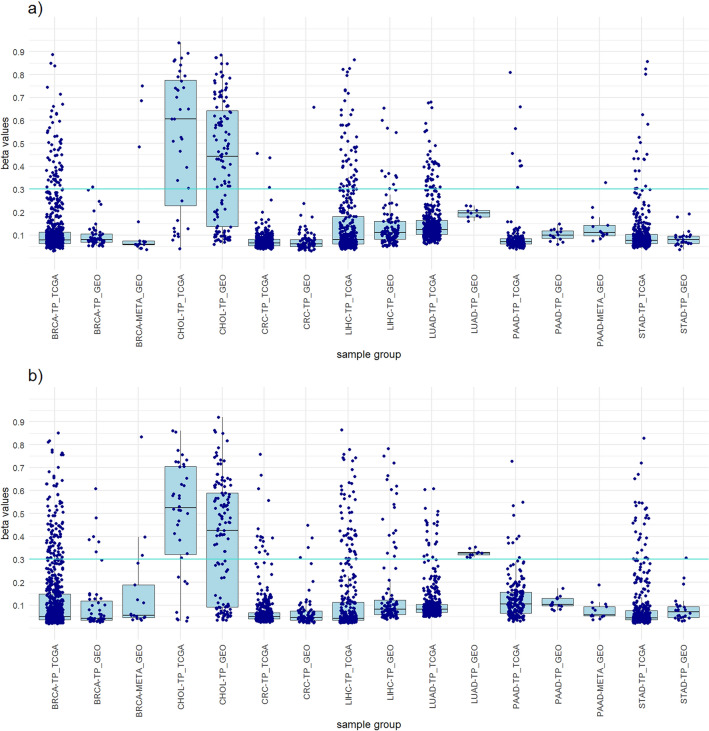
Boxplots showing the distribution of the highest beta values of all included samples from the CHOL panels of both approaches and a comparison between the TCGA dataset and the GEO dataset. (**a**) CHOL panel clustered approach. (**b**) CHOL panel non-clustered approach.

Three probes from non-clustered approach were used in the CHOL panel and achieved a sensitivity of 77.8% and high specificities in TCGA dataset (Table 2). Although adding an additional probe would result in higher sensitivity, the CHOL specificity would decrease. Therefore, we decided to use fewer probes and maintain the high CHOL specificity. The panel with four probes from the clustered approach shows a slightly lower sensitivity (72.2%). Specificity for tumor samples (92.7%), specificity for all samples (93.1%) and diagnostic accuracy (92.8%) were slightly higher compared to the panel from non-clustered approach. Although probe cg02228804 was not included in either panel, it was obtained through both approaches (Supplementary Table S4, Supplementary Table S7 and Supplementary Fig. S2 and Supplementary Fig. S3). The results of the GEO dataset are similar to the results of the TCGA dataset, with slightly lower CHOL sensitivity and similar specificities (Table 2). Similarities can be seen in Fig. 2, which shows the distribution of the highest beta values of all included samples from the panels of both approaches and a comparison between the TCGA dataset and the GEO dataset.

**Table 2:** CHOL panels from clustered and non-clustered approach.

CHOL panel	Selected probes	No. of probes	Primary cancer						All tumor samples			All samples			Statistics				
			No. TS β > 0.3	No. TS β < 0.3	No. all TS	No. NT β > 0.3	No. NT β < 0.3	No. all NT	No. other TS β > 0.3	No. other TS β < 0.3	No. all TS	No. other β > 0.3	No. other β < 0.3	No. all samples	CHOL Sensitivity	CHOL Specificity	TS Specificity	All samples Specificity	Accuracy
TCGA dataset																			
Non-clustered approach	cg27146152 cg18101249 cg11094122	3	28	8	36	0	9	9	235	2338	2609	235	2582	2853	77.8%	100.0%	90.9%	91.7%	91.5%
Clustered approach	cg02228804 cg08099174 cg17218628 cg23661183	4	26	10	36	0	9	9	189	2384	2609	195	2622	2853	72.2%	100.0%	92.7%	93.1%	92.8%
GEO dataset																			
Non-clustered approach	cg27146152 cg18101249 cg11094122	3	69	47	116	0	16	16	39	263	418	69	597	782	59.5%	100.0%	87.1%	89.6%	85.2%
Clustered approach	cg02228804 cg08099174 cg17218628 cg23661183	4	83	33	116	0	16	16	5	297	418	13	653	782	71.6%	100.0%	98.3%	98.0%	94.1%

CHOL sensitivity, CHOL specificity, all tumor samples specificity, all samples specificity and diagnostic accuracy of the panels was calculated.

CHOL, Cholangiocarcinoma; No., Number of samples; TS, Tumor samples; NT, Normal tissues; β, Beta value; TCGA, The Cancer Genome Atlas; GEO, Gene Expression Omnibus.

#### Colorectal cancer (CRC)

**Figure 3:**
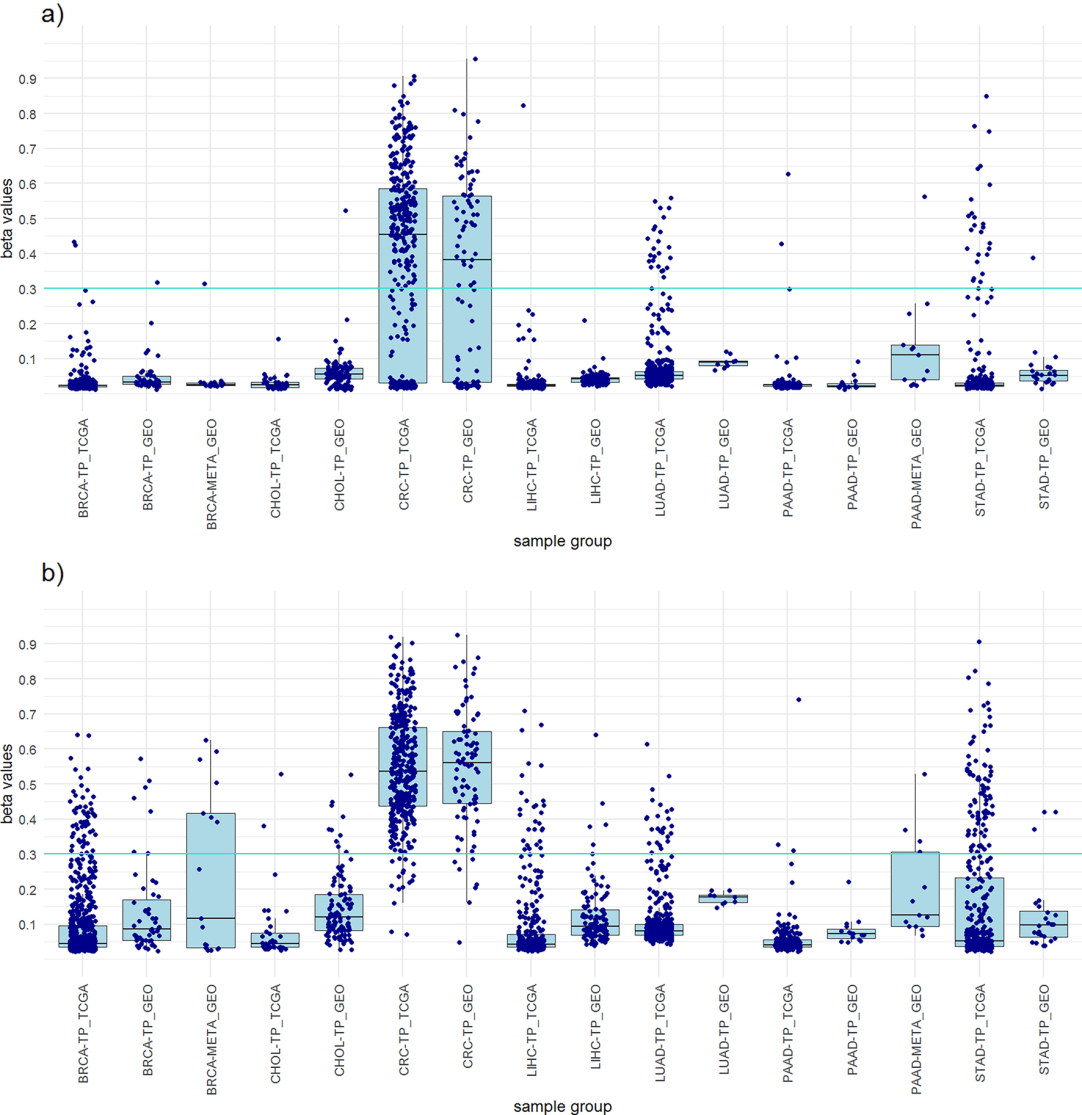
Boxplots showing the distribution of the highest beta values of all included samples from the CRC panels of both approaches and a comparison between the TCGA dataset and the GEO dataset. (**a**) CRC panel clustered approach. (**b**) CRC panel non-clustered approach.

For the CRC panel, four probes were selected from the non-clustered approach and three probes from the clustered approach (Table 3). Although both panels achieved a CRC specificity of 100%, the non-clustered panel yielded a much higher CRC sensitivity than the panel with the clustered approach (95.9% vs. 62.8%). In contrast, slightly higher specificity for all samples and higher diagnostic accuracy were observed with the clustered approach (Table 3). In the clustered approach, an additional probe can be added to achieve higher CRC sensitivity. While specificity for tumor samples and specificity for all samples would remain high, CRC specificity would decrease. Although not included in the final panel design, three identical probes were obtained from both approaches (Supplementary Table S4 and Supplementary Table S7). In the GEO dataset the panels resulted in slightly lower CRC sensitivity and CRC specificity, but achieved higher specificity for tumor samples, specificity for all samples and higher diagnostic accuracy (Table 3). The distribution of the highest beta values of all included samples from the panels of both approaches and a comparison between the TCGA dataset and the GEO dataset are shown in Fig. 3.

**Table 3:** CRC panels from clustered and non-clustered approach.

CRC panel	Selected probes	No. of probes	Primary cancer						All tumor samples			All samples			Statistics				
			No. TS β > 0.3	No. TS β < 0.3	No. all TS	No. NT β > 0.3	No. NT β < 0.3	No. all NT	No. other TS β > 0.3	No. other TS β < 0.3	No. all TS	No. other β > 0.3	No. other β < 0.3	No. all samples	CRC Sensitivity	CRC Specificity	TS Specificity	All samples Specificity	Accuracy
TCGA dataset																			
Non-clustered approach	cg18174928 cg17528648 cg04696193 cg00552973	4	371	16	387	0	45	45	199	2023	2609	199	2267	2853	95.9%	100.0%	91.0%	91.9%	92.5%
Clustered approach	cg05930133 cg14786398 cg01325460	3	243	144	387	0	45	45	56	2166	2609	56	2410	2853	62.8%	100.0%	97.5%	97.7%	93.0%
GEO dataset																			
Non-clustered approach	cg18174928 cg17528648 cg04696193 cg00552973	4	82	7	89	10	71	81	26	303	418	41	652	782	92.1%	87.7%	92.1%	94.1%	93.9%
Clustered approach	cg05930133 cg14786398 cg01325460	3	51	38	89	0	81	81	2	327	418	2	691	782	57.3%	100.0%	99.4%	99.7%	94.6%

CRC sensitivity, CRC specificity, all tumor samples specificity, all tumor samples specificity, all samples specificity and diagnostic accuracy of the panels was calculated.

Abbreviations: CRC, colorectal carcinoma; No., number of samples; TS, Tumor samples; NT, normal tissues; β, beta value; TCGA, The Cancer Genome Atlas; GEO, Gene Expression Omnibus.

#### Lung adenocarcinoma (LUAD)

**Figure 4:**
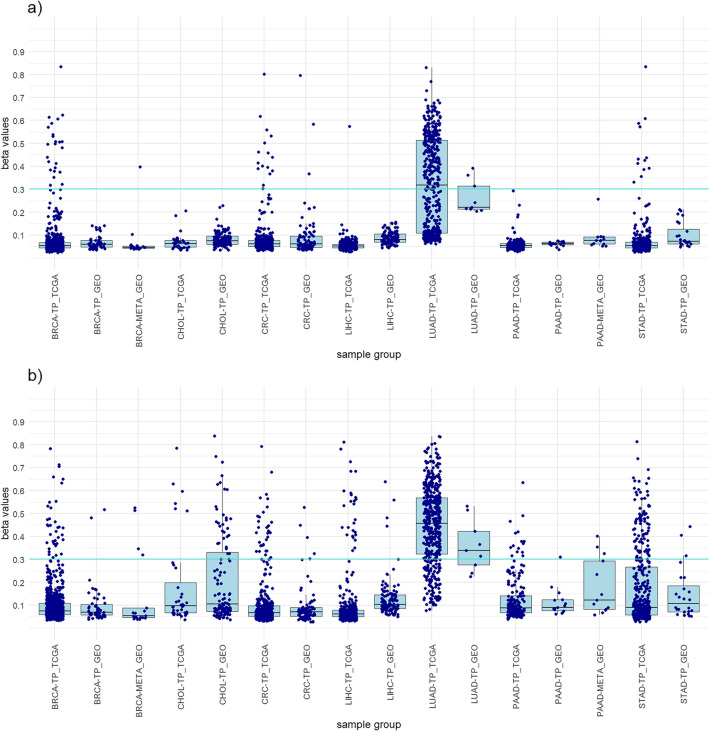
Boxplots showing the distribution of the highest beta values of all included samples from the LUAD panels of both approaches and a comparison between the TCGA dataset and the GEO dataset. (**a**) LUAD panel clustered approach. (**b**) LUAD panel non-clustered approach.

LUAD was the cancer for which the fewest probes were obtained. The non-clustered approach yielded only two probes, cg21929771 is LUAD-specific and cg00907427 is hypermethylated in LUAD tumor samples and normal samples, but hypomethylated in all comparison groups. The panel consisting of these two probes yielded a LUAD sensitivity of 78.9%, a specificity of 89.8% for tumor samples, a specificity of 89.8% for all samples, and a diagnostic accuracy of 88.5% (Table 4). Although the panel is not LUAD specific, it can be used to differentiate between cancer types. To achieve 100% LUAD specificity, we recommend using only the cg21929771 probe, which successfully differentiate LUAD from other samples with a sensitivity of 65.2% and a specificity of 94.5% for all samples (Supplementary Fig. S2). For the non-clustered approach, significant probes were found only in LUAD MC1 (Supplementary Fig. S3). The panel developed from seven probes of the clustered approach resulted in lower LUAD sensitivity (51.8%), but higher specificities. Similar results were observed in the GEO dataset, which are shown graphically in Fig. 4 (Table 4).

**Table 4:** LUAD panels from clustered and non-clustered approach.

LUAD panel	Selected probes	No. of probes	Primary cancer						All tumor samples			All samples			Statistics				
			No. TS β > 0.3	No. TS β < 0.3	No. all TS	No. NT β > 0.3	No. NT β < 0.3	No. all NS	No. other TS β > 0.3	No. other TS β < 0.3	No. all TS	No. other β > 0.3	No. other β < 0.3	No. all samples	LUAD Sensitivity	LUAD Specificity	TS Specificity	All samples Specificity	Accuracy
TCGA dataset																			
Non-clustered approach	cg00907427 cg21929771	2	358	96	454	32	0	32	207	1948	2609	241	2126	2853	78.9%	0.0%	90.4%	89.8%	88.5%
Clustered approach	cg05930133 cg14786398 cg01325460	3	243	144	387	0	45	45	56	2166	2609	56	2410	2853	62.8%	100.0%	97.5%	97.7%	93.0%
GEO dataset																			
Non-clustered approach	cg00907427 cg21929771	2	6	3	9	28	0	28	49	360	418	78	695	782	66.7%	0.0%	88.0%	89.9%	89.6%
Clustered approach	cg01015199 cg01244124 cg02294176 cg08091147 cg09590094 cg12165782 cg14562915	7	3	6	9	0	28	28	3	406	418	22	751	782	33.3%	100.0%	99.3%	97.2%	96.4%

LUAD sensitivity, LUAD specificity, all tumor samples specificity, all samples specificity and diagnostic accuracy of the panels was calculated.

LUAD, Lung adenocarcinoma; No., Number of samples; TS, Tumor samples; NT, Normal tissues; β, Beta value; TCGA, The Cancer Genome Atlas; GEO, Gene Expression Omnibus.

#### Pancreas adenocarcinoma (PAAD)

**Figure 5:**
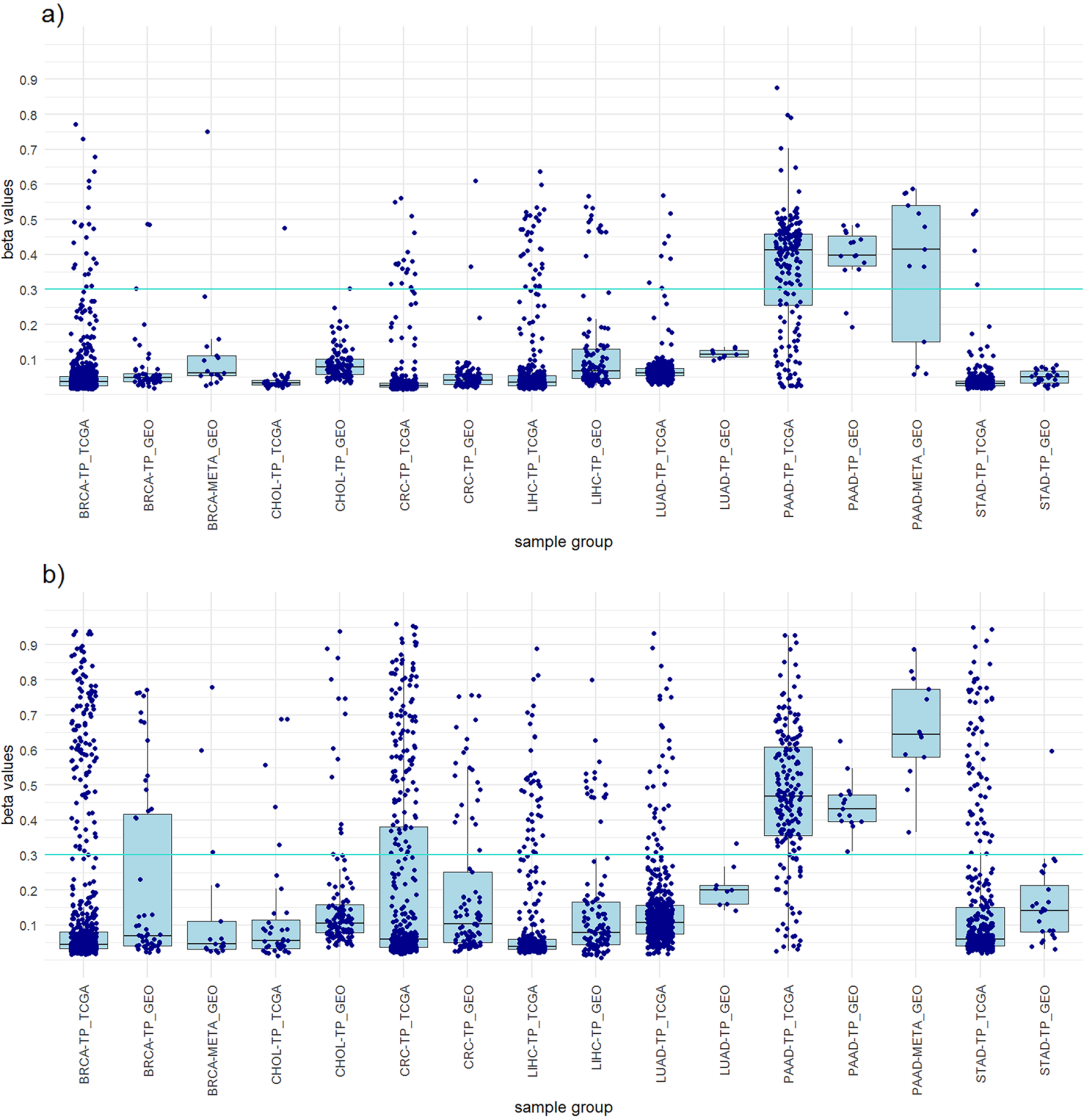
Boxplots showing the distribution of the highest beta values of all included samples from the PAAD panels of both approaches and a comparison between the TCGA dataset and the GEO dataset. (**a**) PAAD panel clustered approach. (**b**) PAAD panel non-clustered approach.

Besides LUAD, PAAD was the one for which only a small number of probes were obtained. The non-clustered approach yielded only two probes, cg01237565 and cg00955911, which are hypermethylated not only in PAAD tumor samples but also in normal samples (Supplementary Table S5). The panel using these two probes detects PAAD with a sensitivity of 82.6% and a diagnostic accuracy of 85.3% (Table 5). The probe cg01237565 was obtained also in clustered approach (Supplementary Fig. S3). This probe was hypermethylated in four of five PAAD clusters. The four-probe PAAD panel developed using the clustered approach shows lower PAAD sensitivity (71.2%) but higher specificity and overall accuracy (94.5%) than the panel developed using the non-clustered approach. Similarly, it shows low PAAD specificity. Although the panels are not PAAD-specific and do not successfully differentiate between PAAD tumor samples and paired normal samples, they can be used to differentiate between included cancer types. Verification of the constructed panels in the GEO dataset confirmed that the panels do not differentiate between PAAD and the adjacent healthy pancreatic tissue, but successfully differentiate PAAD from other included tumors and healthy tissues (Table 5). This can be evident in Fig. 5. For successful differentiation between PAAD tumor samples and paired normal samples, we recommend the use of additional independent methylation biomarkers.

**Table 5:** PAAD panels from clustered and non-clustered approach.

PAAD panel	Selected probes	No. of probes	Primary cancer						All tumor samples			All samples			Statistics				
			No. TS β > 0.3	No. TS β < 0.3	No. all TS	No. NT β > 0.3	No. NT β < 0.3	No. all NT	No. other TS β > 0.3	No. other TS β < 0.3	No. all TS	No. other β > 0.3	No. other β < 0.3	No. all samples	PAAD Sensitivity	PAAD Specificity	TS Specificity	All samples Specificity	Accuracy
TCGA dataset																			
Non-clustered approach	cg01237565 cg00955911	2	152	32	184	8	2	10	374	2051	2609	379	2282	2853	82.6%	20.0%	84.6%	85.8%	85.3%
Clustered approach	cg01237565 cg20178172 cg16788099 cg19326153	4	131	53	184	8	2	10	87	2338	2609	97	2564	2853	71.2%	20.0%	96.4%	96.1%	94.5%
GEO dataset																			
Non-clustered approach	cg01237565 cg00955911	2	15	0	15	3	0	3	69	334	418	88	679	782	100.0%	0.0%	82.9%	88.7%	88.9%
Clustered approach	cg01237565 cg20178172 cg16788099 cg19326153	4	13	2	15	3	0	3	19	384	418	27	740	782	86.7%	0.0%	95.3%	96.5%	96.3%

PAAD sensitivity, PAAD specificity, all tumor samples specificity, all samples specificity and diagnostic accuracy of the panels was calculated.

PAAD, Pancreas adenocarcinoma; No., Number of samples; TS, Tumor samples; NT, Normal tissues; β, Beta value; TCGA, The Cancer Genome Atlas; GEO, Gene Expression Omnibus.

#### Stomach adenocarcinoma (STAD)

**Figure 6:**
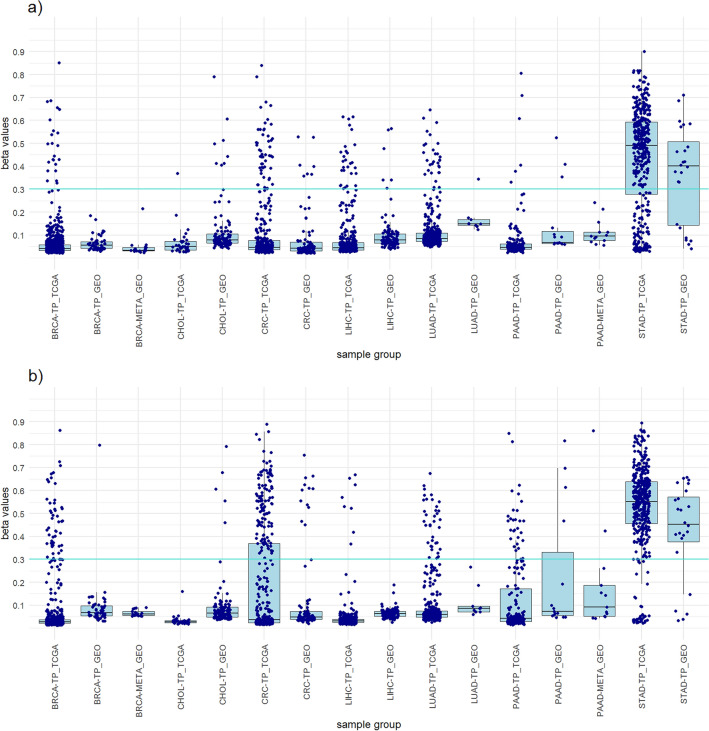
Boxplots showing the distribution of the highest beta values of all included samples from the STAD panels of both approaches and a comparison between the TCGA dataset and the GEO dataset. (**a**) STAD panel clustered approach. (**b**) STAD panel non-clustered approach.

For the STAD panels, three probes were selected from the non-clustered approach and seven probes from the clustered approach (Table 6). Although both panels achieved a STAD specificity of 100%, the non-clustered panel yielded a much higher STAD sensitivity than the panel with the clustered approach (90.1% vs. 73.7%). Slightly higher specificities and higher diagnostic accuracy were observed with the non-clustered approach. The probe cg06622735 was used in both panels (Supplementary Fig. S2 and Supplementary Fig. S3). The results of the GEO dataset show a high degree of agreement with the results of the TCGA dataset, with even higher diagnostic accuracy (Table 6). This can also be seen in the graphic representation in Fig. 6.

**Table 6:** STAD panels from clustered and non-clustered approach.

STSAD panel	Selected probes	No. of probes	Primary cancer						All tumor samples			All samples			Statistics				
			No. TS β > 0.3	No. TS β < 0.3	No. all TS	No. NS β > 0.3	No. NS β < 0.3	No. all NS	No. other TS β > 0.3	No. other TS β < 0.3	No. all TS	No. other β > 0.3	No. other β < 0.3	No. all samples	STSAD Sensitivity	SSTAD Specificity	TS samples Specificity	All samples Specificity	Accuracy
TCGA dataset																			
Non-clustered approach	cg08649919 cg15446670 cg06622735 cg12484686 cg27599958	5	356	39	395	0	2	2	198	2016	2609	238	2220	2853	90.1%	100.0%	94.1%	90.3%	90.3%
Clustered approach	cg06622735 cg17284070 cg06373764 cg21972430 cg15161047 cg14375387 cg04416750	7	291	104	395	0	2	2	138	2076	2609	209	2249	2853	73.7%	100.0%	93.8%	91.5%	89.0%
GEO dataset																			
Non-clustered approach	cg08649919 cg15446670 cg06622735 cg12484686 cg27599958	5	22	2	24	0	84	84	19	375	418	19	739	782	91.7%	100.0%	95.2%	97.5%	97.3%
Clustered approach	cg06622735 cg17284070 cg06373764 cg21972430 cg15161047 cg14375387 cg04416750	7	17	7	24	0	84	84	25	369	418	31	727	782	70.8%	100.0%	93.7%	95.9%	95.1%

PAAD sensitivity, PAAD specificity, all tumor samples specificity, all samples specificity and diagnostic accuracy of the panels was calculated.

STAD, Stomach adenocarcinoma; No., Number of samples; TS, Tumor samples; NT, Normal tissues; β, Beta value; TCGA, The Cancer Genome Atlas; GEO, Gene Expression Omnibus.

#### Breast invasive carcinoma (BRCA)

**Figure 7:**
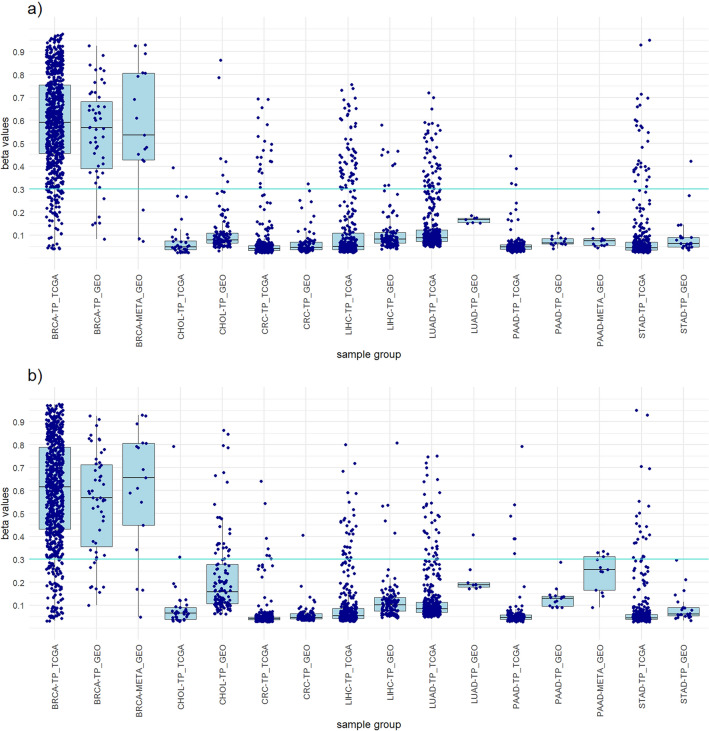
Boxplots showing the distribution of the highest beta values of all included samples from the BRCA panels of both approaches and a comparison between the TCGA dataset and the GEO dataset. (**a**) BRCA panel clustered approach. (**b**) BRCA panel non-clustered approach.

BRCA panels consisted of 7 and 10 selected probes from the non-clustered and clustered approaches, respectively. Both panels differentiate BRCA from other cancers with high sensitivity and specificity (Table 7). The panel from the clustered approach resulted in slightly higher BRCA sensitivity than the panel from the non-clustered approach (91.8% vs. 88.9%). In contrast, the latter resulted in higher BRCA specificity (96.9% vs. 94.8%), higher specificity for tumor samples (94.1% vs. 90.6%), higher specificity for all samples (94.7% vs. 91.4%), and higher diagnostic accuracy (93% vs. 91.5%). Both panels included probes cg17652435, cg02085210 and cg02435495. All those probes were hypermethylated in multiple BRCA clusters (Supplementary Table S8). The panels tested on GEO dataset also resulted in high sensitivities, specificities and diagnostic accuracies (Table 7). This can be seen in Fig. 7, which shows the distribution of the highest beta values of all included samples from the panels of both approaches and a comparison between the TCGA dataset and the GEO dataset.

**Table 7:** BRCA panels from clustered and non-clustered approach.

BRCA panel	Selected probes	No. of probes	Primary cancer						All tumor samples			All samples			Statistics				
			No. TS β > 0.3	No. TS β < 0.3	No. all TS	No. NT β > 0.3	No. NT β < 0.3	No. all NT	No. other TS β > 0.3	No. other TS β < 0.3	No. all TS samples	No. other β > 0.3	No. other β < 0.3	No. all samples	BRCA Sensitivity	BRCA Specificity	TS Specificity	All samples Specificity	Accuracy
TCGA dataset																			
Non-clustered approach	cg16005540 cg02435495 cg17652435 cg02085210 cg22783363 cg23051664 cg25147193	7	690	86	776	3	93	96	109	1724	2609	110	1964	2853	88.9%	96.9%	94.1%	94.7%	93.0%
Clustered approach	cg24797187 cg18565473 cg01698108 cg05709124 cg02435495 cg09463047 cg17652435 cg00091827 cg02085210 cg09679923	10	712	64	776	5	91	96	173	1660	2609	173	1899	2853	91.8%	94.8%	90.6%	91.4%	91.5%
GEO dataset																			
Non-clustered approach	cg16005540 cg02435495 cg17652435 cg02085210 cg22783363 cg23051664 cg25147193	7	43	8	51	0	104	104	33	334	418	35	696	782	84.3%	100.0%	91.0%	95.2%	94.5%
Clustered approach	cg24797187 cg18565473 cg01698108 cg05709124 cg02435495 cg09463047 cg17652435 cg00091827 cg02085210 cg09679923	10	44	7	51	2	102	104	17	350	418	19	712	782	86.3%	98.1%	95.4%	97.4%	96.7%

BRCA sensitivity, BRCA specificity, all tumor samples specificity, all samples specificity and diagnostic accuracy of the panels was calculated.

BRCA, Breast invasive carcinoma; No., Number of samples; TS, Tumor samples; NT, Normal tissues; β, Beta value; TCGA, The Cancer Genome Atlas; GEO, Gene Expression Omnibus.

#### Liver metastases

The designed PAAD and BRCA panels were tested in metastatic tumor samples. The PAAD panel from the non-clustered approach showed excellent 100% sensitivity for PAAD liver metastases and good specificities (Table 8). In contrast, the PAAD panel from the clustered approach showed a lower sensitivity but higher specificities between 92.4 and 94.9%. Both BRCA panels showed a sensitivity of 83.3% for BRCA liver metastases. Although the panel from the clustered approach achieved higher specificities than the panel from the non-clustered approach, both panels resulted in a high diagnostic accuracy (89.5% and 91.9%) (Table 8). The conservation of methylation from primary tumors to liver metastases and their cancer specificity is presented in Figs. 5 and 7.

**Table 8:** The results of testing PAAD and BRCA panels in PAAD liver metastases and BRCA liver metastases.

Cancer	Approach	Selected probes	No. of probes	Metastases						Tumor samples			All samples			Statistics				
				No. MTS β > 0.3	No. MTS β < 0.3	No. all MTS	No. Other MTS β > 0.3	No. Other MTS β < 0.3	No. all other MTS	No. other TS β > 0.3	No. other TS β < 0.3	No. all TS	No. other β > 0.3	No. other β < 0.3	No. all samples	Metastases sensitivity	Metastases specificity	TS Specificity	All samples Specificity	Accuracy
GEO dataset																				
PAAD LM	Non-clustered approach	cg01237565 cg00955911	2	13	0	13	3	15	18	88	350	449	107	695	813	100.0%	83.3%	79.8%	86.8%	86.8%
PAAD LM	Clustered approach	cg01237565 cg20178172 cg16788099 cg19326153	4	9	4	13	1	17	18	33	404	449	41	760	813	69.2%	94.4%	92.4%	94.9%	94.5%
BRCA LM	Non-clustered approach	cg16005540 cg02435495 cg17652435 cg02085210 cg22783363 cg23051664 cg25147193	7	15	3	18	4	9	13	80	352	449	82	714	813	83.3%	69.2%	81.4%	89.7%	89.5%
BRCA LM	Clustered approach	cg24797187 cg18565473 cg01698108 cg05709124 cg02435495 cg09463047 cg17652435 cg02085210 cg09679923	9	15	3	18	0	13	13	61	371	449	63	733	813	83.3%	100.0%	85.8%	92.1%	91.9%

Metastases sensitivity, Metastases specificity (specificity between included metastases), tumor samples specificity (including primary tumors and metastases), all samples specificity (including primary tumors, normal tissue samples and metastases) and the diagnostic accuracy of the panels were calculated.

PAAD, Pancreas adenocarcinoma; BRCA, Breast invasive carcinoma; No., Number of samples; MTS, Metastatic tumor samples; TS, Tumor samples; β, Beta value; LM, Liver metastases; TCGA, The Cancer Genome Atlas; GEO, Gene Expression Omnibus.

#### Cell-free DNA

The design panels were tested on the available EPIC data from cfDNA samples from the GSE122126 dataset^12^. Three healthy individuals, three patients with CRC liver metastases and three patients with BRCA liver metastases, were included. Using our panels, we successfully detected cfDNA hypermethylation in two out of three patients with CRC liver metastases and in all three patients with BRCA liver metastases. All selected biomarkers were unmethylated in the cfDNA of healthy donors. The beta values for the selected biomarkers and the included cfDNA samples are listed in Supplementary Table S9.

## Discussion

While a significant proportion of liver malignancies are primary tumors, the occurrence of liver metastases originating from adenocarcinomas is relatively common and frequently observed in clinical practice^3,27^. Sometimes the primary site of a metastatic tumor cannot be determined. These tumors, termed CUP, are frequently found in the liver^4,28^. The differential diagnosis and origin of metastatic adenocarcinoma are usually determined by histomorphologic examination and immunohistochemical studies^3,27^. However, in some cases, primary liver adenocarcinomas are poorly differentiated and indistinguishable from metastatic adenocarcinoma and the primary site of a metastatic tumor cannot be determined^3,29^. Therefore, the importance of genetics and epigenetics in differential diagnosis is increasing. Given the extensive research on epigenetic changes in cancer, DNA methylation is one of the most thoroughly studied. To identify new potential DNA methylation biomarkers, we focused on methylation data from the HM450, which includes approximately 450,000 CpGs in the human genome. This platform focuses on CpG islands and gene promoters that are typically unmethylated in healthy tissue and hypermethylated in cancer. This enabled the identification of hypermethylated regions, even though the majority of the cancer genome is typically hypomethylated^30^.

Although many DNA methylation biomarkers have been identified, most previous cancer research based on HM450 methylation array data mainly focused on the abnormal methylation patterns in a cancer or use a large number of probes for successful cancer differentiation. Some research groups have successfully identified potential DNA methylation markers that can differentiate and successfully diagnose various cancers^31–39^. Hao et al. successfully differentiate LUAD, LIHC, COAD, BRCA and adjacent normal tissues based on their DNA methylation profiles using machine learning on HM450 TCGA data. Moreover, they successfully identified majority of CRC liver metastases with a panel of 46 CpG biomarkers, which support the potential for using the DNA methylation signatures to improve the diagnosis of CUP in the liver^35^. Recent study accurately categorized samples of 19 tumor types (including BRCA, COAD, LUAD, LIHC and STAD) according to histology with diagnostic accuracy of 86%^39^. They used random forest model to derived 305-probe classifier set. Tang et al. achieve excellent performance using random forest models on 14 tumor types using between 9 and 738 CpG sites^17^. The results of these studies are not directly comparable with our results because the same cancer types were not included. Nevertheless, they show that high accuracies can be achieved, which is further confirmed by our study. The important difference is that significantly fewer methylation biomarkers were used in our study to achieve similar accuracies. Ding et al. conducted one of the most promising study on TCGA HM450 data. Group used machine learning to narrow down 12 CpG sites that could effectively differentiate between tumor samples for 26 main TCGA cancers. In addition, the group demonstrated that the proposed biomarkers can be extended to metastases and predict the origin of CUP^10^. Although the drawback of this study is that no clinical validation was performed, this study demonstrates that a small number of methylation biomarkers can be used to differentiate multiple cancer types. Despite the fact that the methods in our study are not consistent with theirs, our study supports their findings and shows that high diagnostic sensitivity and specificity can be achieved with a small number of methylation biomarkers. Furthermore, we have not come across any study that uses and compares the results of our two selected approaches.

In our study, we identified DMRs that are hypermethylated in the cancers of interest, whereas they are unmethylated in comparable cancers and the majority of normal tissue samples. Most identified DMRs can be used as methylation biomarkers that can successfully diagnose primary cancer and also differentiate it from adjacent healthy tissue. Furthermore, because hypermethylation of some otherwise methylation-resistant sites occurs early in cancer development and remains methylated at advanced stages, our biomarkers could be a valuable tool for differential diagnosis between primary and metastatic adenocarcinomas of the liver^3,11,13^. This positions them as a potentially invaluable resource for predicting tumor origin in patients with CUP, particularly in the liver, as selected cancers represent the two most common primary liver cancers and frequent sources of liver metastases^1–4^. Two different approaches were used to identify novel methylation biomarkers that were further used to design panels that successfully differentiate between included cancer types.

The first approach was a non-clustered approach in which no clustering of methylation data was performed. This approach resulted in much higher numbers of hypermethylated probes after the filtering process in some cancers, such as LIHC, STAD, BRCA, and CRC, than in others. The cancer-specific panels developed from these probes have the highest sensitivity, specificity and diagnostic accuracy (Tables 1, 3, 6 and 7). As expected, the panels for cancers with a low initial number of probes such as CHOL, LUAD and PAAD have lower sensitivity and specificity. Nevertheless, relatively high diagnostic accuracy is observed for these panels, ranging from 85.3 to 91.5% (Tables 2, 4 and 5).

In the second, clustered approach, unsupervised clustering was performed within each project prior to probe selection. It has been mainly used to identify methylation subtypes with distinct clinicopathologic, molecular and immunologic features, predictive and prognostic subtypes, and methylation-based classification^40–59^. In some studies, methylation clustering has been used as a preliminary step in diagnostic biomarker selection^44,49^. In our study, clustering allowed us to identify MCs within each cancer type based on their methylation patterns. Each MC is represented by a group of samples that, based on their methylation signatures, belong to a specific methylation subtype within each cancer type. Candidate probes were selected for each MC. Probes hypermethylated in multiple MCs generally resulted in higher overall cancer sensitivity than probes hypermethylated in only one MC. Probes selected from different MCs within a cancer type were combined into a cancer-specific panel. Because probes from different clusters detect specific subsets of samples within a cancer type, combining these probes should result in detection of the majority of tumor samples. Our results support this assumption. Panels that included probes from all tumor-related MCs showed high sensitivity. For example, the probes included in the LIHC panel from the clustered approach were selected from all six LIHC MCs. The combination of the selected probes resulted in a LIHC sensitivity of 89.1% (Table 1). In CRC, the significantly hypermethylated probes were obtained in all four MCs. Only three probes that were hypermethylated in CRC-MC1 to CRC-MC3 were included in the final panel design. They yielded a CRC sensitivity of 62.8%. Although inclusion of probe cg01655898, which is also hypermethylated in CRC-MC4 (Table 3), would result in higher CRC specificity (79%), high tumor samples specificity (97.6%) and all samples specificity (97.3%), we decided not to include it in the final panel because the CRC specificity would decrease dramatically. Probe cg01655898 is hypermethylated in CRC and adjacent healthy tissue samples and does not differentiate between them. Nevertheless, it can be used as an additional methylation biomarker that successfully differentiate between included cancers because it has a high specificity for CRC over other included cancers (99.8%). For some cancers, not all MCs resulted in significant probes. As expected, panels that did not contain probes from all MCs of a cancer type have lower sensitivity. The absence of probes hypermethylated in selected cancer types and hypomethylated in other cancer types was most frequently observed in MCs with the lowest average methylation levels (Supplementary Table S6). In PAAD, STAD and BRCA probes were not found in MCs with the lowest average methylation values (PAAD-MC5, STAD-MC5, STAD-MC6, and BRCA-MC7). In LUAD, only LUAD-MC1 resulted in significantly hypermethylated probes (Supplementary Table S6). Surprisingly, probes were obtained in CHOL clusters with lower average methylation, but not in CHOL-MC1. Using our cut-of criteria, we obtained probes that were hypomethylated in CHOL-MC1. However, when we removed LIHC from the probe selection criteria, the hypermethylated probes were identified. This observation leads us to suggest that the absence of hypermethylated probes may be due to the inherent similarities between different adenocarcinomas, particularly in this context between LIHC and CHOL. Since both tumors share the same tissue of origin—the liver—they naturally exhibit overlapping methylation features. Furthermore, cases of combined hepatocellular and cholangiocarcinomas further complicate the differentiation of primary liver malignancies, as such a primary liver tumor has features of both LIHC and CHOL^60^.

The results show that both approaches successfully identified several candidate probes that can be used as methylation biomarkers for the included cancer types. Although most of the probes obtained by the two approaches were different, some of the same candidate probes were found in all cancer types except LUAD. In the clustered approach, most of them were found in multiple MCs. This is not surprising since these probes are significant in a large number of tumor samples within the cancer of interest and therefore similar to the probes identified by the non-clustered approach. Some of these probes were used in cancer-specific panels from both approaches (e.g. LIHC, PAAD, STAD and BRCA panels). A trend was observed in the specificity and sensitivity of the panels. In general, as the sensitivity of the panels increased, the specificity for tumor samples and the specificity for all samples decreased (Tables 1, 2, 3, 4, 5, 6 and 7). The majority of panels from the non-clustered approach yielded higher sensitivity with still very high sensitivity than panels from the clustered approach (Tables 1, 2, 3, 4, 5, 6 and 7). In contrast, the clustered BRCA panel yielded higher BRCA sensitivity but lower specificity (Table 7). Given the relatively high sensitivity and specificity, both BRCA panels can be considered appropriate. The same is true for other cancer-specific panels (e.g. LIHC and CHOL).

To achieve greater credibility of the results and evaluate the performance of the designed panels, we perform a verification on an independent dataset from the GEO database. The 782 samples of primary tumors and normal tissues from the GEO database were used (Supplementary Table S2). The results obtained with the GEO dataset were very similar to those of the TCGA dataset and mostly achieved the same high sensitivities and high specificities for individual cancer types (Tables 1, 2, 3, 4, 5, 6 and 7). The high concordance of the results confirms the suitability of our approaches and ensures that the observed results are not specific to the features of the identification dataset (Figs. 1, 2, 3, 4, 5, 6 and 7).

To increase clinical significance of our results, the design panels were tested in metastatic tumor samples. EPIC methylation data from two primary adenocarcinomas that had metastasized to the liver were acquired from the GEO database (GSE217384 and GSE212375)^14,61^. The data included 31 liver metastases (13 PAAD liver metastases and 18 BRCA liver metastases). The best design panels show a sensitivity of 100% for PAAD liver metastases and a sensitivity of 83.3% for BRCA liver metastases. The design panels also show high specificity for liver metastases, all tumor samples and all samples included in the GEO dataset (Table 8). These results are consistent with studies suggesting that aberrant hypermethylation of CpG islands occurs early in cancer development and is maintained from the primary tumor to its metastases^11,13,14^. The recent study, whose samples were also included in our analysis, showed that the 5000 most variably methylated CpGs exhibited remarkable conservation of cancer-associated hypermethylation between the primary tumor and metastases^14^. Although a different set of probes was used, our results show the same trend. The conservation of methylation from primary tumors to liver metastases and their cancer specificity can be seen in Figs. 5 and 7.

To increase the clinical applicability of our results, we have shown that our biomarkers can be successfully extended to liquid biopsies from cancer patients with metastatic disease. Using our panels, we were able to successfully detect hypermethylation of selected regions in cfDNA from patients with CRC liver metastases and BRCA liver metastases, while these were unmethylated in cfDNA from healthy donors (Supplementary Table S9). All this strongly support that DNA methylation biomarkers can be used as potential diagnostic biomarkers to predict tumor origin in patients with metastatic cancer in cfDNA. In addition, the study from which our cfDNA data were derived showed how cfDNA methylation could be the basis for a non-invasive approach to identify the origin of CUP. They were able to successfully predict the origin of cfDNA in CUP patients with metastatic cancer.

To better understand the selected DMRs and their potential role in tumor biology, we performed an annotation of the selected probes (Supplementary Table S10). Most of the selected probes are located in promoter regions of genes and miRNAs or at CCTC binding factor (CTCF) binding sites. It is noteworthy that some of selected probes and their corresponding genes have already been associated with cancer. Hypermethylation of these regions has been associated with epigenetic reprogramming, tumorigenesis, metastasis and poor prognosis^62–66^. In addition, promoter regions have been associated with downregulation of the corresponding genes^37,62–64,67–72^. Some of the selected regions have already been identified as potential diagnostic methylation biomarkers that differentiate between primary tumors, adjacent normal tissue samples and other tumor types^37,62,66,69,70,72–77^. In addition, the aberrant methylation of annotated genes has been identified as a diagnostic methylation biomarker in liquid biopsies^78,79^.

To further evaluate the suitability of the proposed panels, the developed panels should be verified using additional data with tissue samples and cfDNA samples, including patients with primary tumors and liver metastases.

## Conclusion

With this study, we have identified and verified novel DNA methylation biomarkers that successfully differentiate selected adenocarcinomas based on HM450 and EPIC DNA methylation data from TCGA and GEO. Two different approaches were used to identify hypermethylated probe candidates: a non-clustered approach (no clustering was performed) and a clustered approach (unsupervised clustering was performed within each project, followed by probe selection for each cluster). Two panels of selected methylation biomarkers from each approach were developed for each cancer type on the TCGA dataset. To demonstrate the robustness of our results, the panels were verified on an independent GEO dataset, which shows a high agreement with the TCGA dataset. The majority of the panels exhibit high sensitivity and specificity, suggesting that both approaches may be useful in the search for novel methylation biomarkers that differentiate primary cancers and adjacent normal tissues, differentiate between different cancer types and can be extended to liver metastases. To increase the clinical relevance of our findings, we have shown that our biomarkers can be detected in liquid biopsies from cancer patients with liver metastases. Using our panels, we were able to detect hypermethylation of selected regions in the cfDNA of patients with metastatic disease, while these were unmethylated in the cfDNA of healthy donors. We believe that the developed panels have potential for the diagnosis of selected primary adenocarcinomas, the characterization of liver metastases and the determination of cancer origin in CUP in the liver using tissue samples and cfDNA.

## Methods

### TCGA data download and preparation

For data collection and bioinformatics analysis software environment and language R were used^80^. HM450 array DNA methylation data for selected projects (BRCA, CHOL, COAD, LIHC, LUAD, PAAD, READ and STAD) were downloaded from the National Cancer Institute's Genomic Data Commons Data Portal (GDC), which is part of TCGA^81^. We used level three data, which represent beta value (level of methylation) for each individual probe, for primary tumor samples and normal tissue samples of each cancer project. All selected samples were fresh tissue samples. Formalin-fixed paraffin-embedded tissue samples, representing only a few samples in each project, were excluded from further analysis. For selected samples, probes on the X and Y chromosome were removed to avoid gender influence in downstream differential analyses. In addition, probes with missing data and duplicated measurement were removed. In the identification step, the data were analyzed using two different approaches: a non-clustered approach (no sample clustering was performed) and a clustered approach (unsupervised sample clustering was performed within each project). Separate results were obtained for each of these approaches. Simple study workflow is presented in Supplementary Fig. S4.

### Clustering

Clustering was performed with recursively petitioned mixture model (RPMM), which has been applied extensively for clustering large-scale genomic data^82^. The RPMM is a model-based unsupervised clustering algorithm developed for beta-distributed DNA methylation measurement. RPMM clustering was performed on 5000 probes that showed the most variable methylation levels in tumor samples for each project. For initialization of a latent class model, a fanny algorithm (a fuzzy clustering algorithm) was used. A level-weighted version of the Bayesian information criterion (BIC) was used as a split criterion for an existing cluster. The MC were denoted according to the average methylation level per cluster: the cluster with the highest methylation value was defined as MC1, the cluster with the second highest methylation value as MC2, and so on. Although, the clustering was performed separately on each project, the exceptions were the COAD and READ projects, which were merged and further addressed as CRC. The normal tissue samples of the individual projects were not clustered (Supplementary Fig. S5).

### Differentially methylated regions analysis

DMR analyses were performed in both, non-clustered and clustered approach (Supplementary Fig. S5). In the non-clustered approach, DMR analysis was performed on tumor samples of each project and normal tissue samples of each project, both representing an independent group. DMR analyses were performed between all groups of tumor samples and normal tissue samples. In the clustered approach, each cluster assigned to the corresponding tumor samples represented an independent group. The normal tissue samples of the individual projects were kept as independent groups. Similar to the non-clustered approach, DMR analyses were performed between all groups. The exception was groups of clusters assigned to the same cancer type. In total, we performed more than 700 DMR analyses.

DMR analysis was performed using TCGAbiolinks package^80^. The difference between the mean methylation (mean beta-values) of each group for each probe was calculated. The *p*-value was calculated using the Wilcoxon test using the Benjamini–Hochberg adjustment method (adjusted *p*-value). The cut-off parameters for DMR were set: minimum mean difference in methylation between compared groups had to be 0.2 and the adjusted *p*-value had to be 0.05 or smaller.

### Selection of differentially methylated probes

According to the DMR analyses, the probes in each comparison were classified as significant (probes in which the minimum mean difference in methylation level between the group of interest and the comparison group was greater than 0.2 and the adjusted *p*-value was 0.05 or less) and not significant (probes that did not meet the cutoff criteria). The significant probes in each comparison were used as the basis for further selection.

In the non-clustered approach, the significant probes from DMR analyses comparing the cancer type of interest with other cancer types were intersected. For each cancer type, the probes that were hypermethylated in the cancer of interest and hypomethylated in the compared cancers were selected. As part of the clustered approach, the probe selection was performed for each cluster within each cancer type. Probe intersection was used to extract the probes that were differentially methylated in the MC of interest compared with all MCs from other cancer types. For the each MC, the probes that were hypermethylated in the MC of interest and hypomethylated in the compared MCs were selected.

The cutoff criteria for the probe selection in both approaches were that the difference in average methylation between the group of interest and the comparison groups had to be greater than 0.3 and the methylation in the comparison groups had to be less than 0.1. In cases where few or no significant probes were found, we introduced less stringent criteria: the difference in average methylation between the group of interest and the compared groups had to be greater than 0.2 and/or the methylation in the comparison groups had to be less than 0.15. The probes obtained were verified in normal tissue samples (Supplementary Fig. S5).

### Panels design

To achieve better differentiation between selected cancer types, we designed a methylation biomarker panel for each cancer type. Where possible, we designed two panels for each cancer type: one with the probes obtained using a non-clustered approach and one with the probes obtained using a clustered approach. The probes with the highest average methylation in the investigated cancer were preferentially selected for the panel design. Probes detected in multiple clusters associated with the cancer type of interest were preferentially selected for panel design from the clustered dataset. Probes that were hypermethylated in cancer of interest and hypomethylated in normal tissue samples were preferentially selected in both approaches. The combination of the smallest possible number of hypermethylated probes that resulted in the highest sensitivity and highest specificity was selected for the final panel design.

### Probes and panels testing

For selected probes and panels, experimental data (beta values) were checked for each sample in all investigated projects. The probes and panels were evaluated according to how many individual samples could be detected in investigated project and in the comparison projects based on their beta values. A beta value of 0.3 was chosen as the cut-off criterion. Sensitivity and specificity of the probes and panels to differentiate between cancer of interest and normal tissue samples were calculated. In addition, the sensitivity and specificity to differentiate between all cancer types (all primary tumor samples) and all included samples (all primary tumor samples and normal tissue samples) were calculated. For the panels, diagnostic accuracy was calculated^83^. The statistics was performed with R package EpiR^84^.

### Verification of the results

The verification of the results was performed on an independent dataset from the GEO database (Fig. 1). HM450 and EPIC array DNA methylation data for selected projects comprising selected adenocarcinomas, available liver metastases and cfDNA (GSE75041, GSE113017^85^, GSE113019^85^, GSE217384^61^, GSE201241^86^, GSE49656^87^, GSE220160, GSE119526^88^, GSE149282^89^, GSE159898^90^, GSE63704^91^, GSE134217^92^, GSE207846^93^, GSE99553^94^, GSE100503^95^, GSE88883^96^, GSE212375^14^, GSE122126^12^) were downloaded. Where available, the raw data were downloaded and used to perform quality control, normalization and calculation of the beta value with the minfi package^97^. In this case, only samples that passed quality check were included. For projects for which no raw data were available, we used the project data provided by the authors for which quality check and normalization had already been performed. The data were downloaded and the beta values were extracted using the GEOquery package^98^. In the verification step, the experimental data (beta values) of the selected probes were checked for each sample in all projects. A beta value of 0.3 was selected as the cut-off criterion for a probe and thus for a sample to be labelled as hypermethylated. Panels were evaluated according to how many individual samples could be correctly classified based on their beta values. Sensitivity, specificity and diagnostic accuracy of the panels were calculated and compared with those from the TCGA dataset.

### Annotation of selected probes

Annotation of selected probes were performed using Ensemble Release 110^99^.

### Supplementary Information

Supplementary Figures.

Supplementary Tables.

## Data Availability

The datasets analyzed in this study are available from The Cancer Genome Atlas (https://portal.gdc.cancer.gov/) or Gene Expression Omnibus (https://www.ncbi.nlm.nih.gov/geo/). All data generated or analyzed during this study are included in this published article and its additional files.
