# Supplementary material for: Discovery of novel DNA methylation biomarker panels for the diagnosis and differentiation between common adenocarcinomas and their liver metastases
Source: Sci Rep. 2024 Feb 7;14:3095. doi: 10.1038/s41598-024-53754-1 (PMC10850119; doi:10.1038/s41598-024-53754-1)
Supplement: Supplementary file 1 — Supplementary Figures. [file 41598_2024_53754_MOESM1_ESM.pdf]

Discovery of Novel DNA Methylation Biomarker Panels for the Diagnosis and Differentiation between Common Adenocarcinomas and Their Liver Metastases

Tina Draškovič, Nina Hauptman

Institute of Pathology, Faculty of Medicine, University of Ljubljana, Ljubljana, Slovenia

Supplementary Figures

Fig. S1: DNA methylation heatmaps showing HM450 DNA methylation levels and RPMM-based classification of included adenocarcinomas.

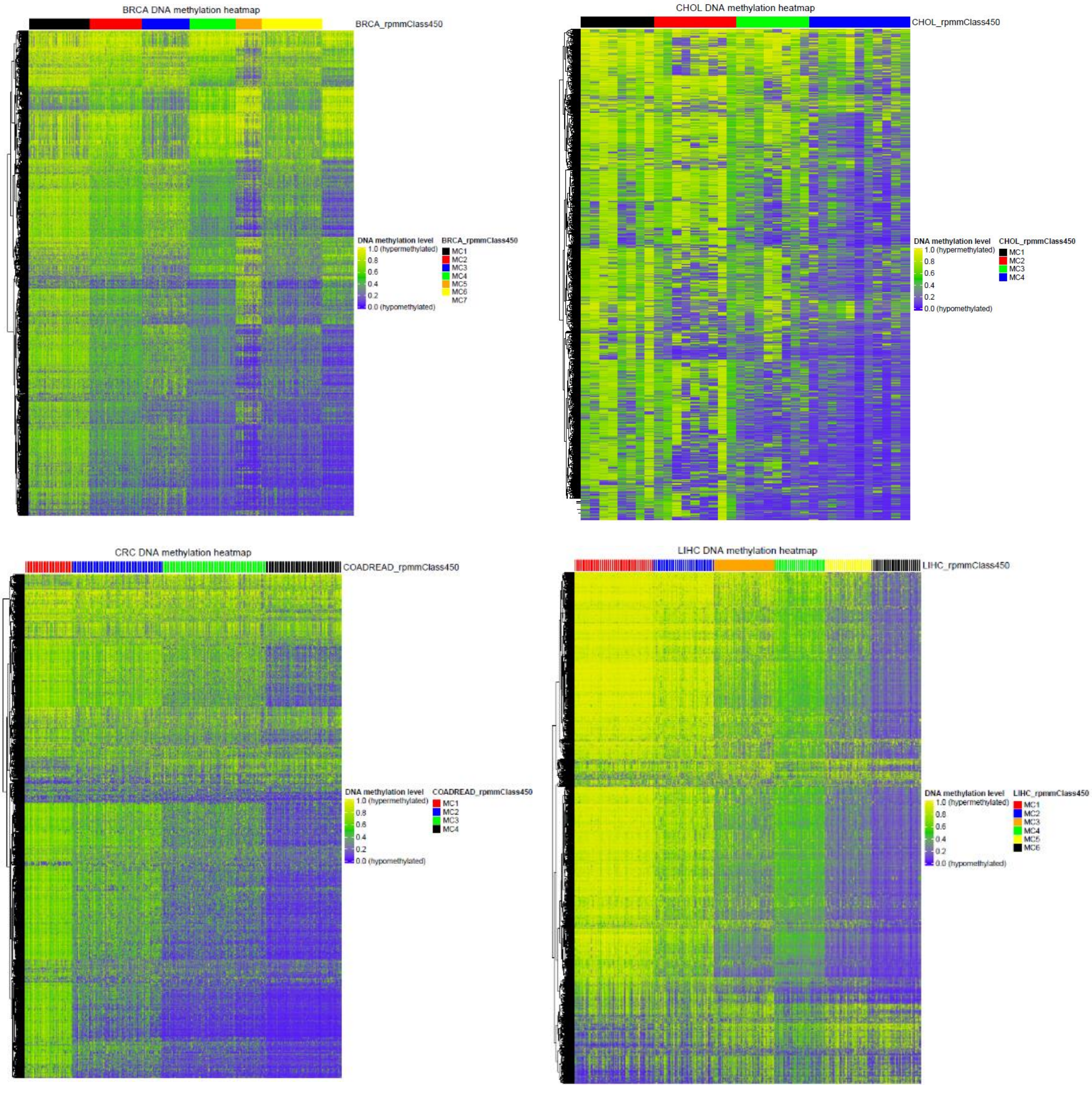

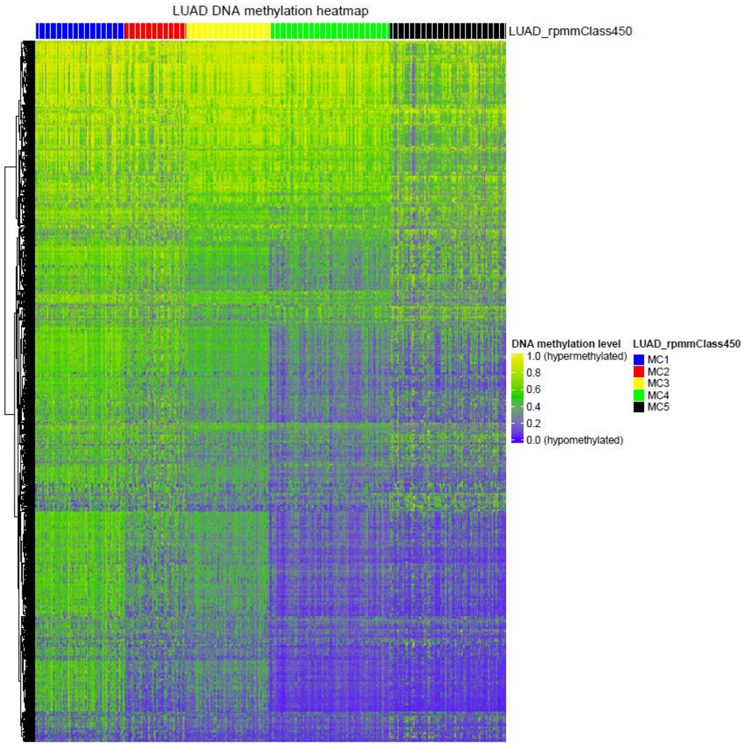
LUAD DNA methylation heatmap
LUAD_rpmmClass450
DNA methylation level
1.0 (hypermethylated)
0.8
0.6
0.4
0.2
0.0 (hypomethylated)
LUAD_rpmmClass450
MC1
MC2
MC3
MC4
MC5

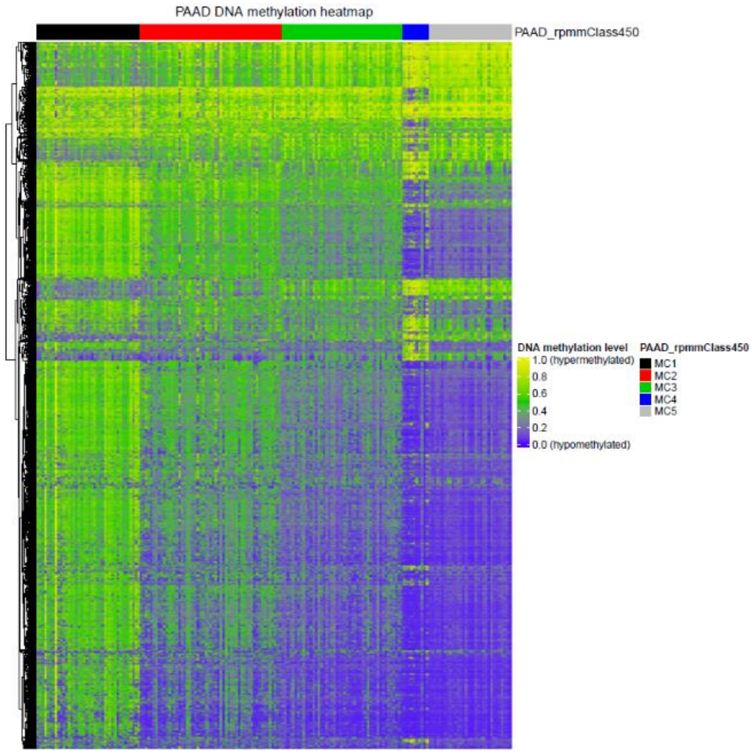
PAAD DNA methylation heatmap
PAAD_rpmmClass450
DNA methylation level
1.0 (hypermethylated)
0.8
0.6
0.4
0.2
0.0 (hypomethylated)
PAAD_rpmmClass450
MC1
MC2
MC3
MC4
MC5

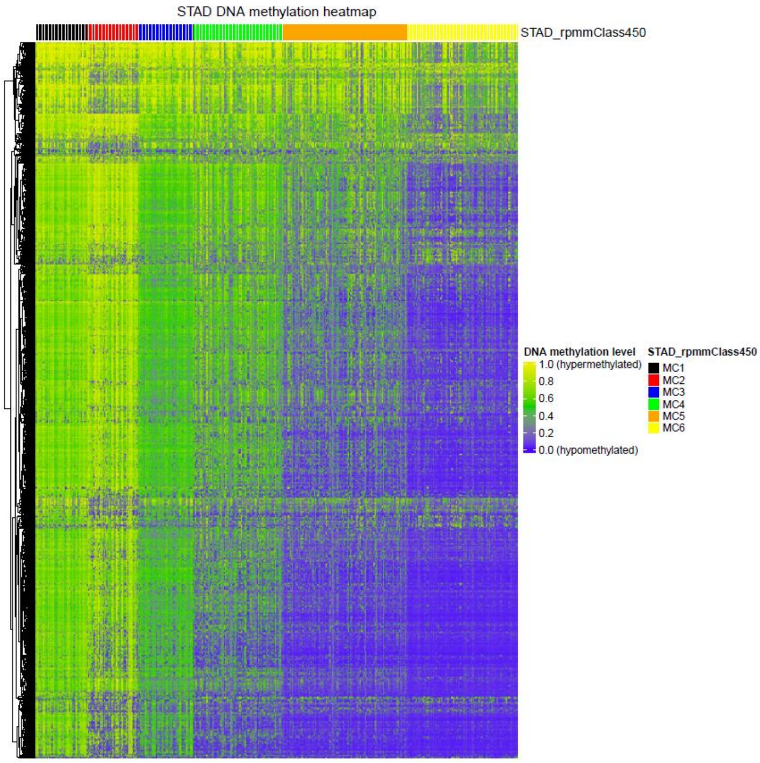
STAD DNA methylation heatmap
STAD_rpmmClass450
DNA methylation level
1.0 (hypermethylated)
0.8
0.6
0.4
0.2
0.0 (hypomethylated)
STAD_rpmmClass450
MC1
MC2
MC3
MC4
MC5
MC6

Fig. S2: Boxplots showing the distribution of beta values of selected probes from the non-clustered approach across the included tumor samples and the normal tissue samples.

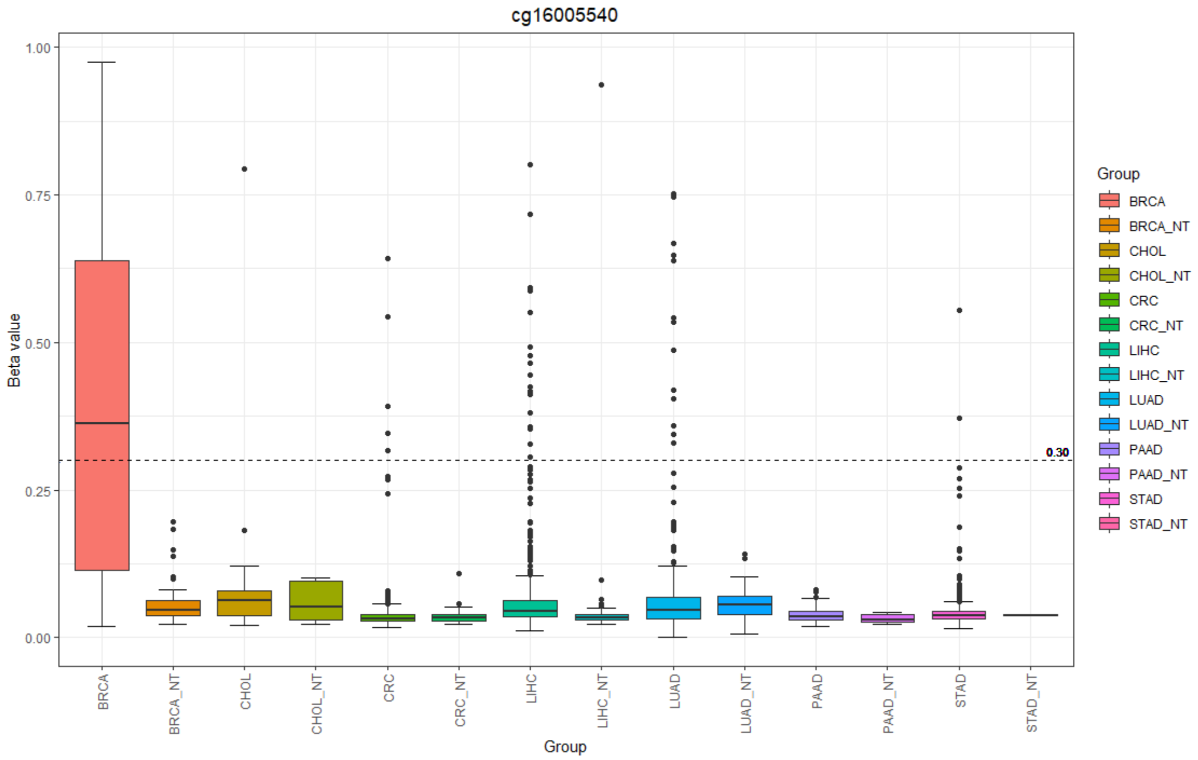

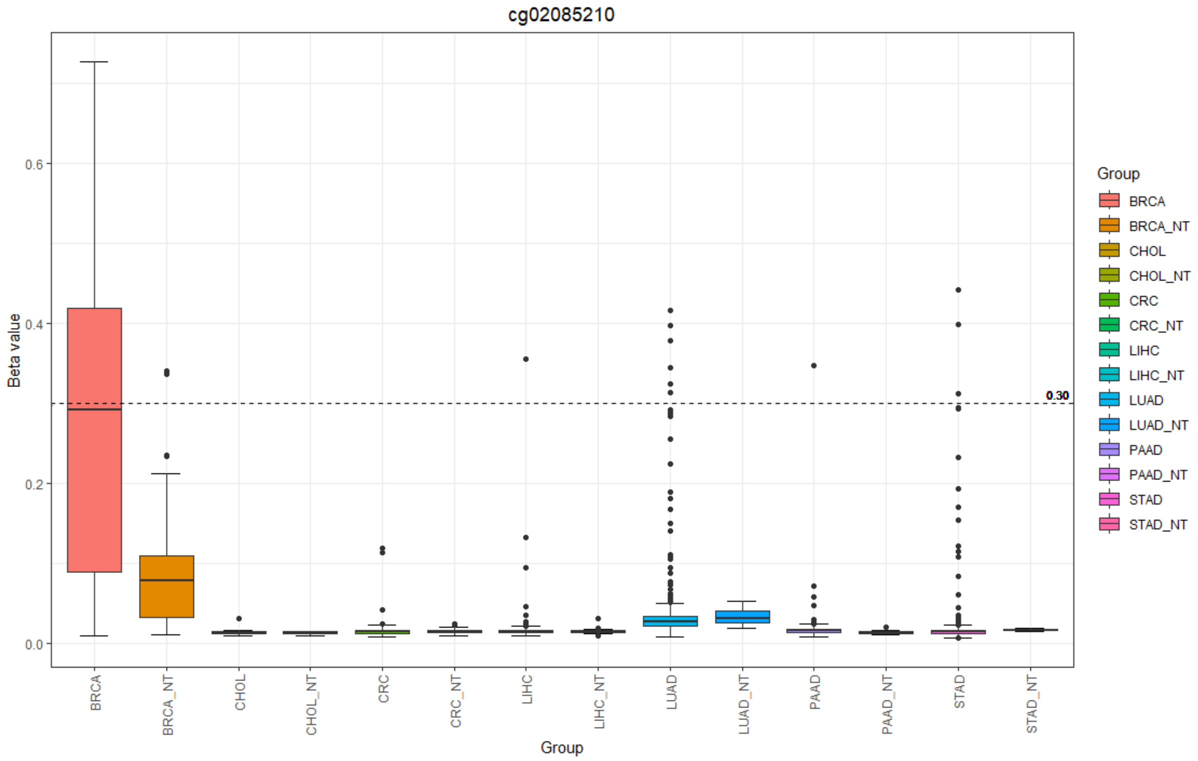

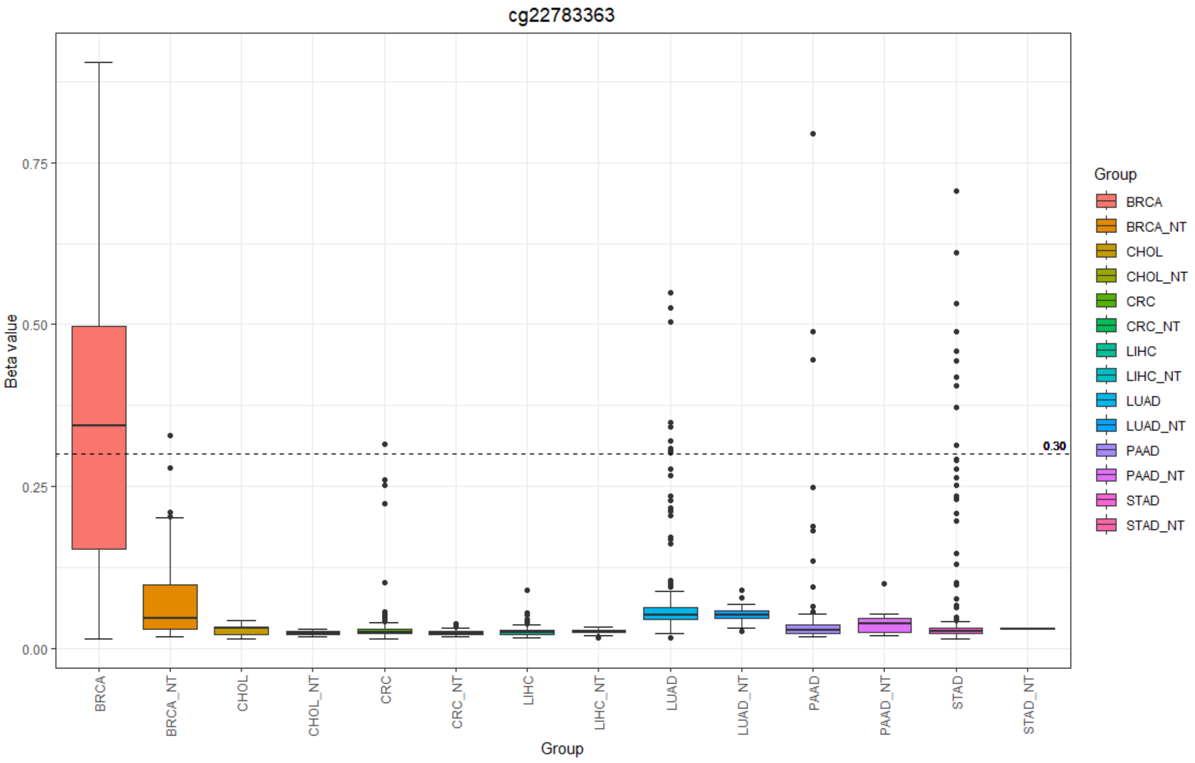
cg22783363
Beta value
0.00
0.25
0.50
0.75
0.30
Group
BRCA
BRCA_NT
CHOL
CHOL_NT
CRC
CRC_NT
LIHC
LIHC_NT
LUAD
LUAD_NT
PAAD
PAAD_NT
STAD
STAD_NT

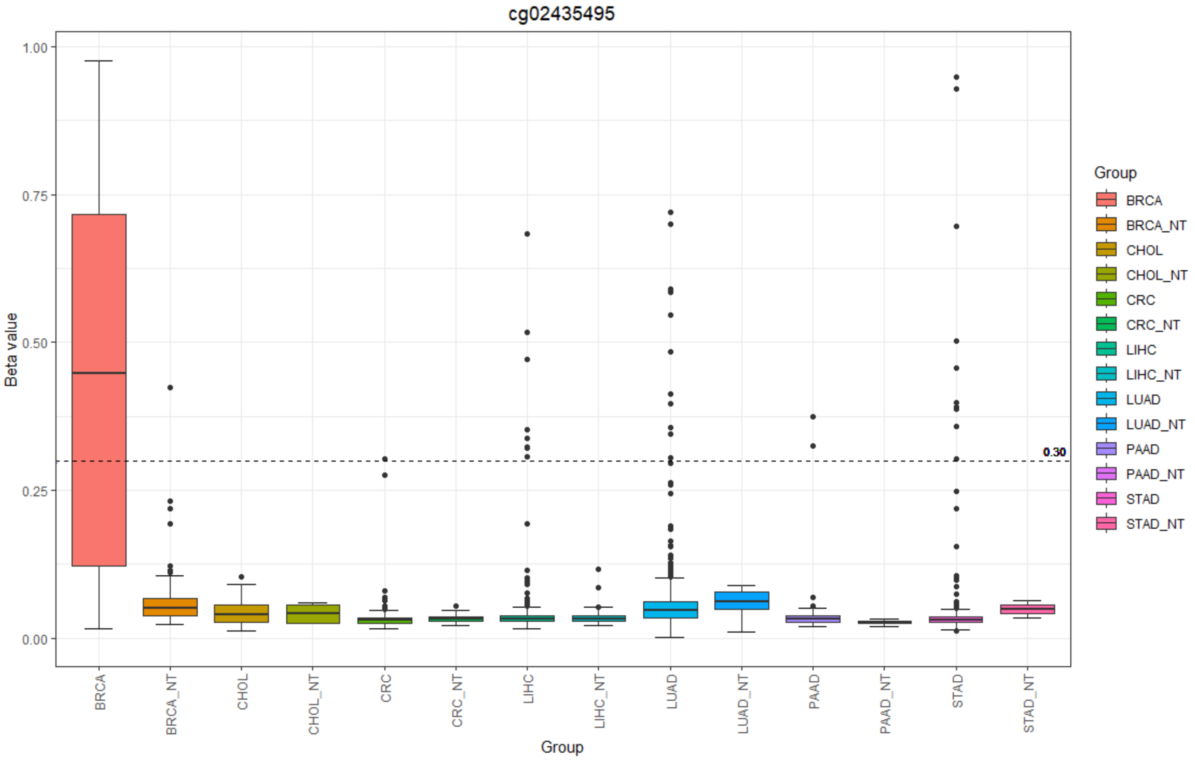
cg02435495
Beta value
0.00
0.25
0.50
0.75
1.00
0.30
Group
BRCA
BRCA_NT
CHOL
CHOL_NT
CRC
CRC_NT
LIHC
LIHC_NT
LUAD
LUAD_NT
PAAD
PAAD_NT
STAD
STAD_NT

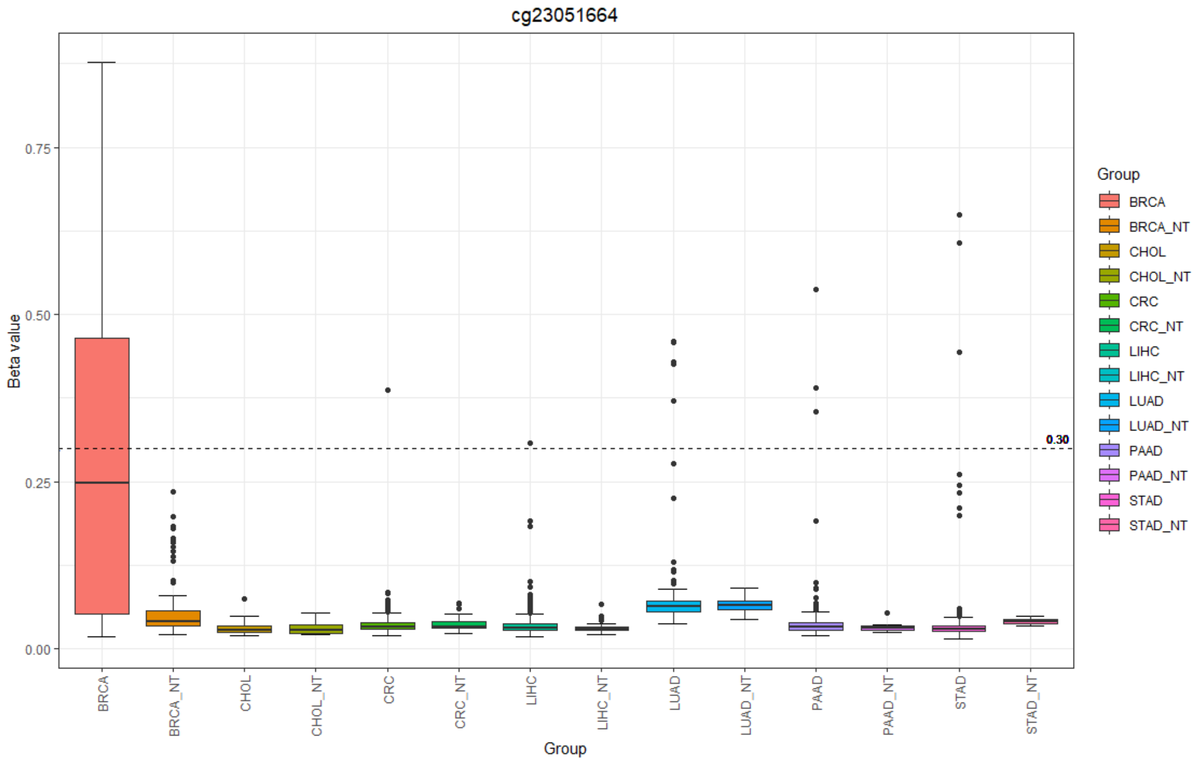
cg23051664
Beta value
0.75
0.50
0.25
0.00
0.30
Group
BRCA
BRCA_NT
CHOL
CHOL_NT
CRC
CRC_NT
LIHC
LIHC_NT
LUAD
LUAD_NT
PAAD
PAAD_NT
STAD
STAD_NT

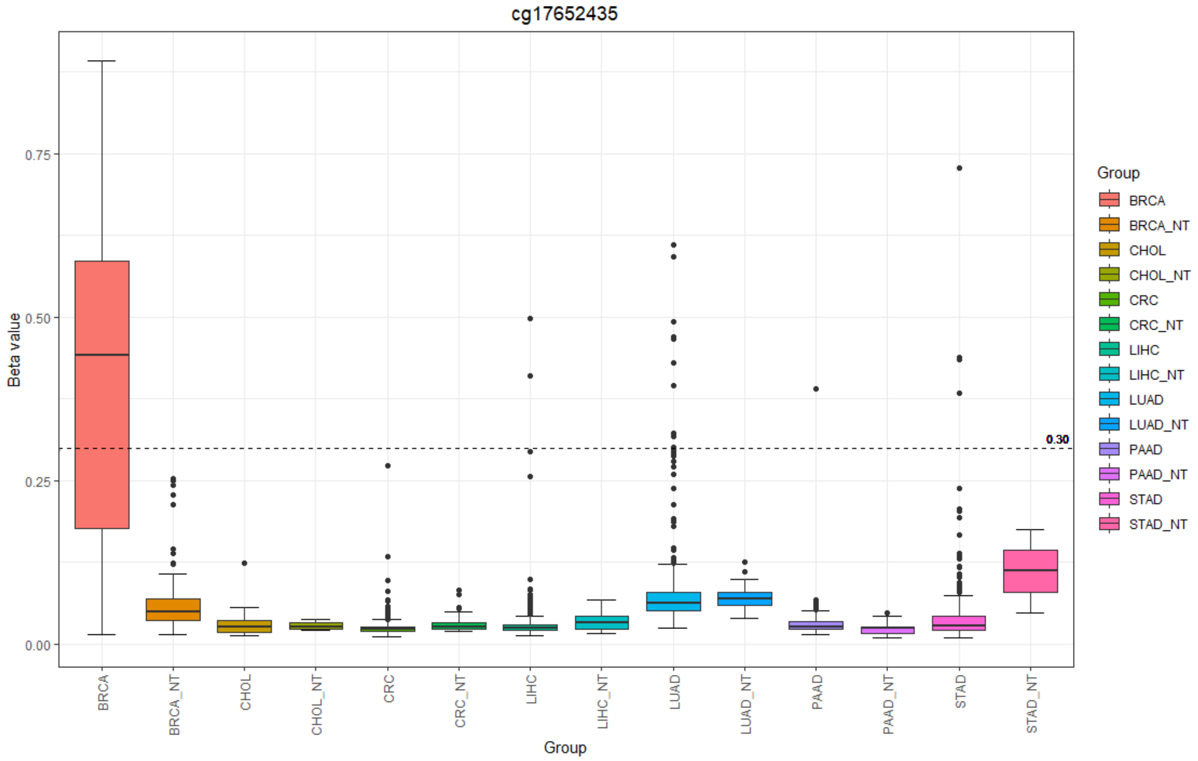
cg17652435
Beta value
0.75
0.50
0.25
0.00
0.30
Group
BRCA
BRCA_NT
CHOL
CHOL_NT
CRC
CRC_NT
LIHC
LIHC_NT
LUAD
LUAD_NT
PAAD
PAAD_NT
STAD
STAD_NT

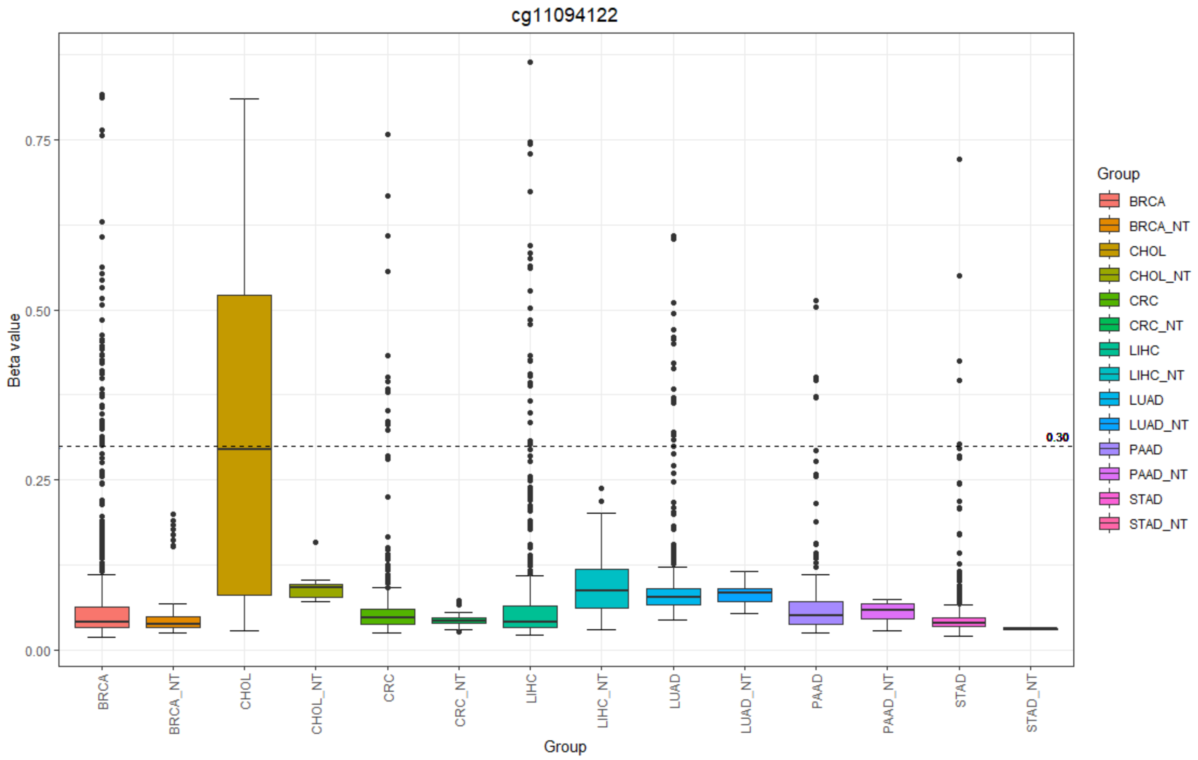
cg11094122
Beta value
0.00
0.25
0.50
0.75
0.30
BRCA
BRCA_NT
CHOL
CHOL_NT
CRC
CRC_NT
LIHC
LIHC_NT
LUAD
LUAD_NT
PAAD
PAAD_NT
STAD
STAD_NT
Group
Group
BRCA
BRCA_NT
CHOL
CHOL_NT
CRC
CRC_NT
LIHC
LIHC_NT
LUAD
LUAD_NT
PAAD
PAAD_NT
STAD
STAD_NT

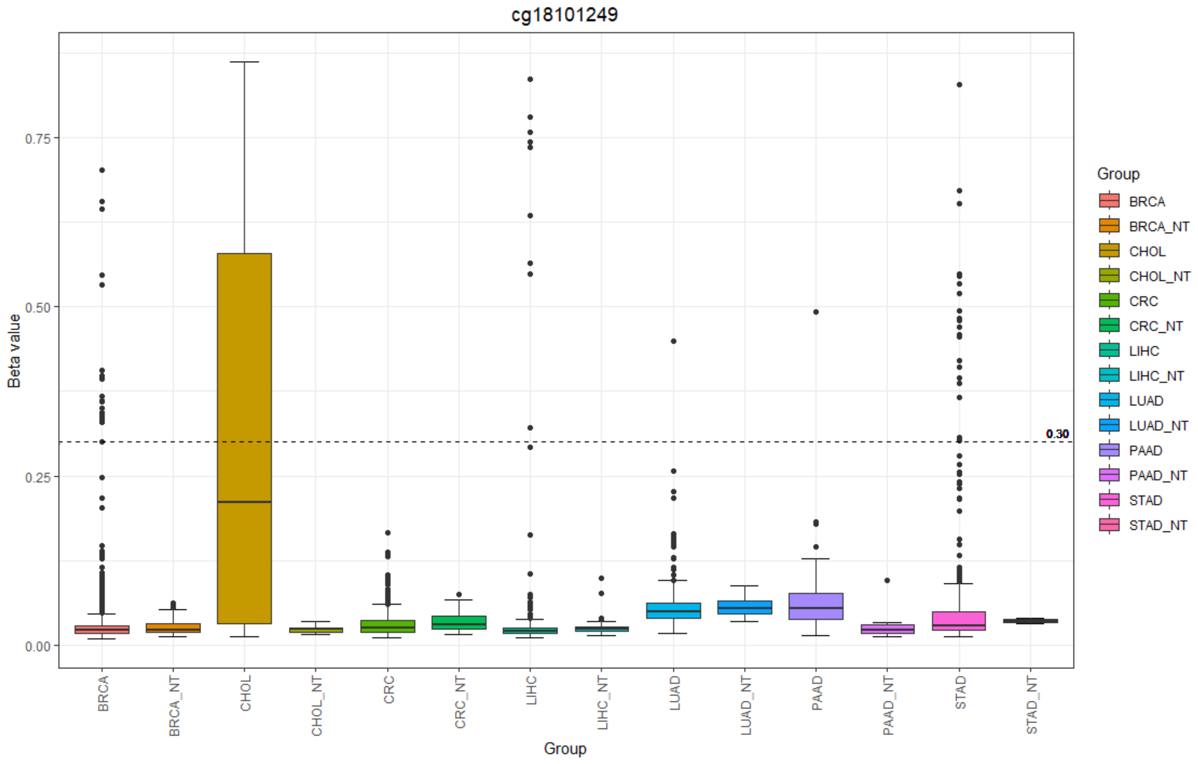
cg18101249
Beta value
0.00
0.25
0.50
0.75
0.30
BRCA
BRCA_NT
CHOL
CHOL_NT
CRC
CRC_NT
LIHC
LIHC_NT
LUAD
LUAD_NT
PAAD
PAAD_NT
STAD
STAD_NT
Group
Group
BRCA
BRCA_NT
CHOL
CHOL_NT
CRC
CRC_NT
LIHC
LIHC_NT
LUAD
LUAD_NT
PAAD
PAAD_NT
STAD
STAD_NT

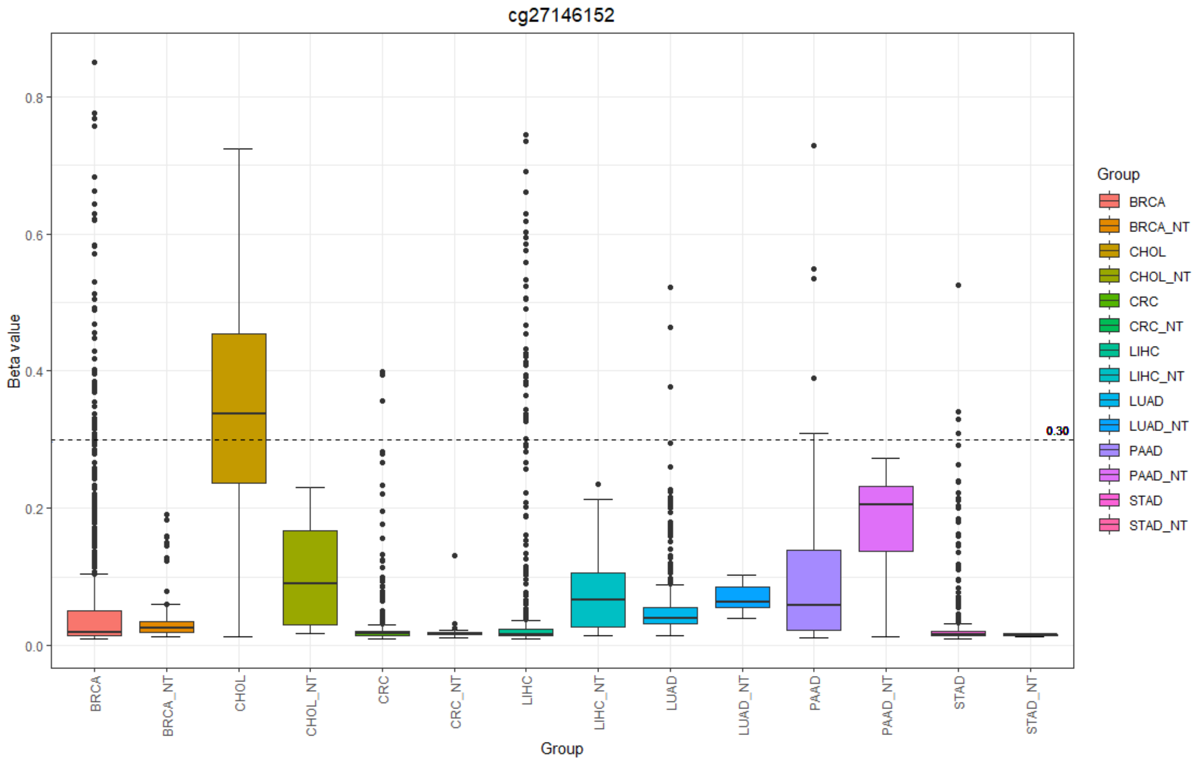
cg27146152
Beta value
0.0
0.2
0.4
0.6
0.8
0.30
Group
BRCA
BRCA_NT
CHOL
CHOL_NT
CRC
CRC_NT
LIHC
LIHC_NT
LUAD
LUAD_NT
PAAD
PAAD_NT
STAD
STAD_NT

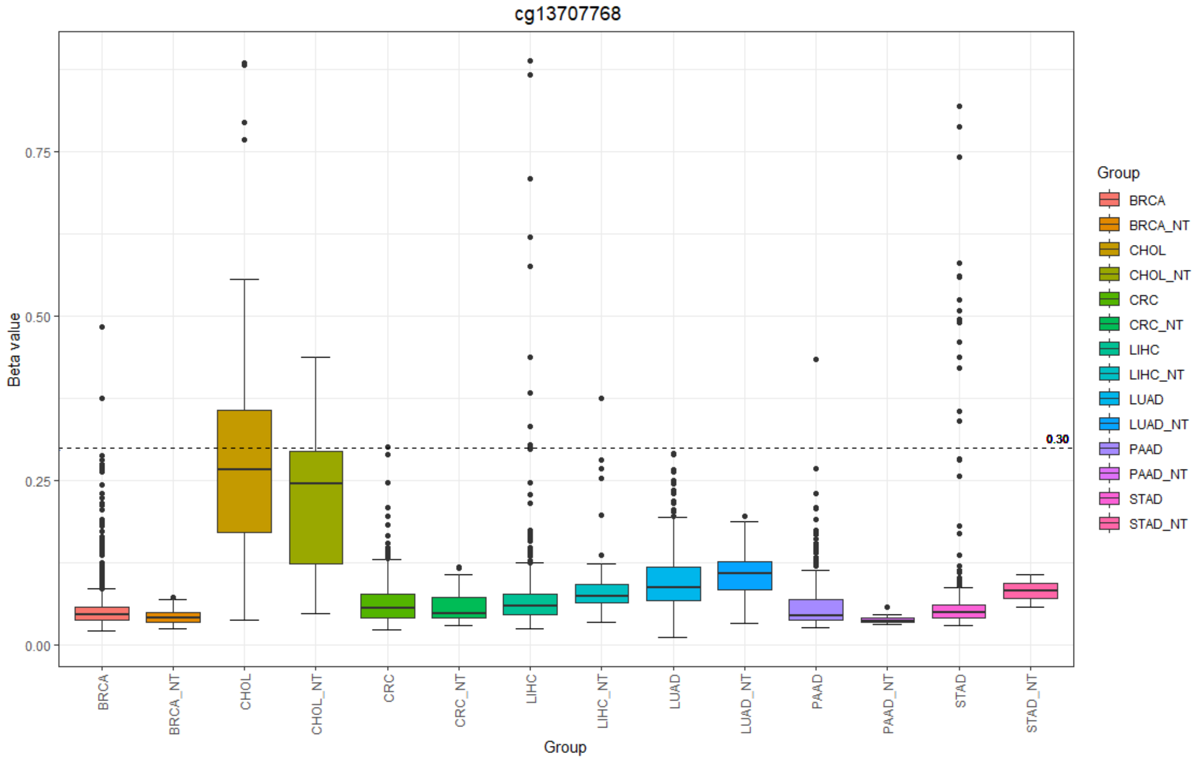
cg13707768
Beta value
0.00
0.25
0.50
0.75
0.30
Group
BRCA
BRCA_NT
CHOL
CHOL_NT
CRC
CRC_NT
LIHC
LIHC_NT
LUAD
LUAD_NT
PAAD
PAAD_NT
STAD
STAD_NT

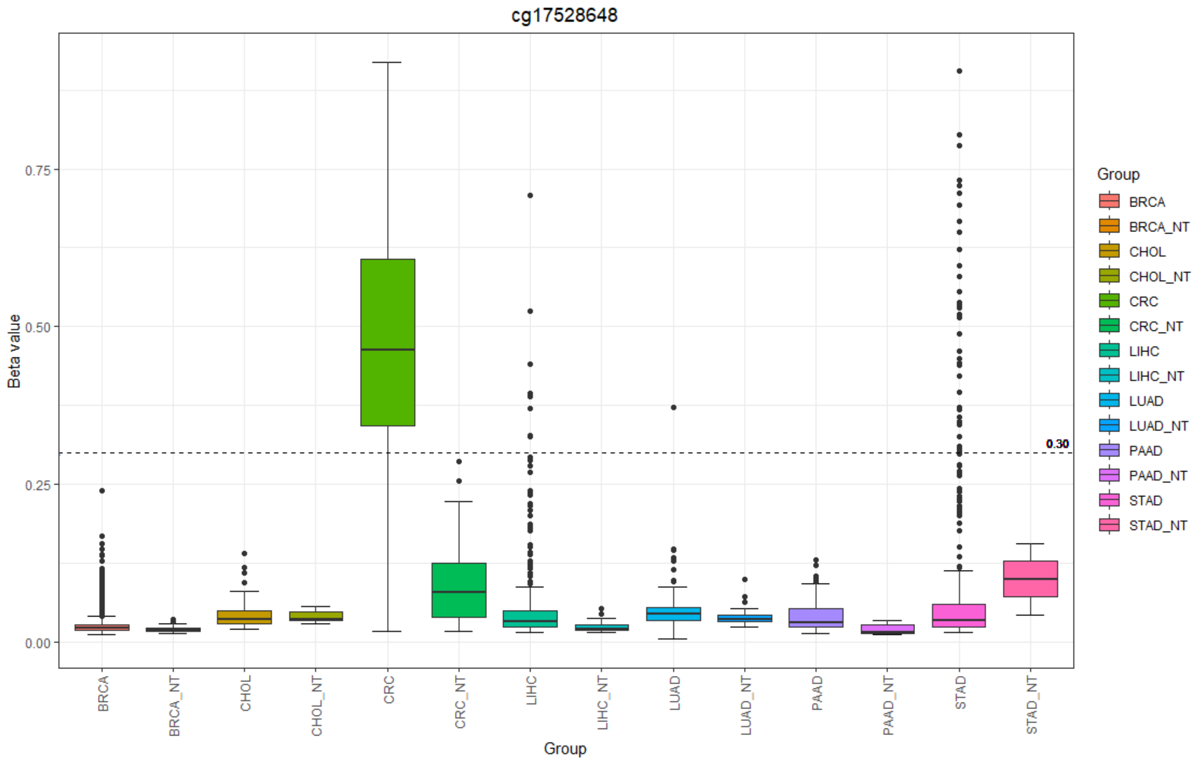
cg17528648
Beta value
0.00
0.25
0.50
0.75
0.30
Group
BRCA
BRCA_NT
CHOL
CHOL_NT
CRC
CRC_NT
LIHC
LIHC_NT
LUAD
LUAD_NT
PAAD
PAAD_NT
STAD
STAD_NT

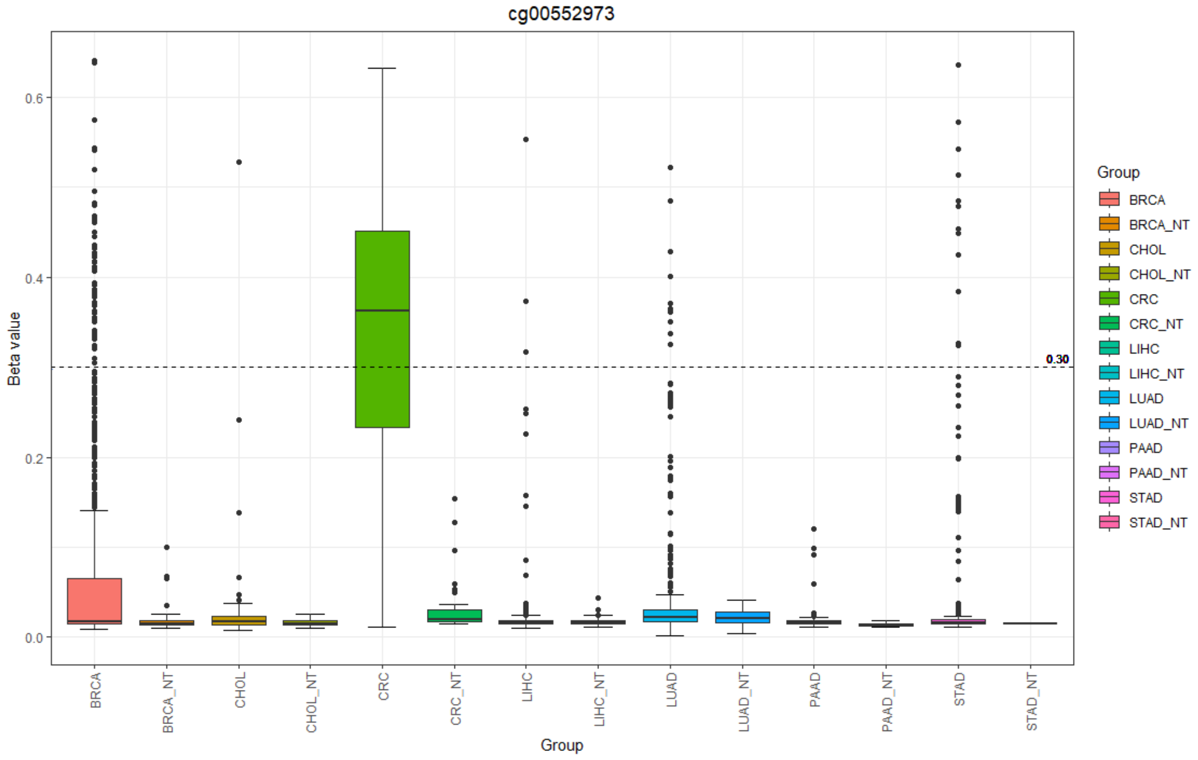
cg00552973
Beta value
0.0
0.2
0.4
0.6
0.30
Group
BRCA
BRCA_NT
CHOL
CHOL_NT
CRC
CRC_NT
LIHC
LIHC_NT
LUAD
LUAD_NT
PAAD
PAAD_NT
STAD
STAD_NT

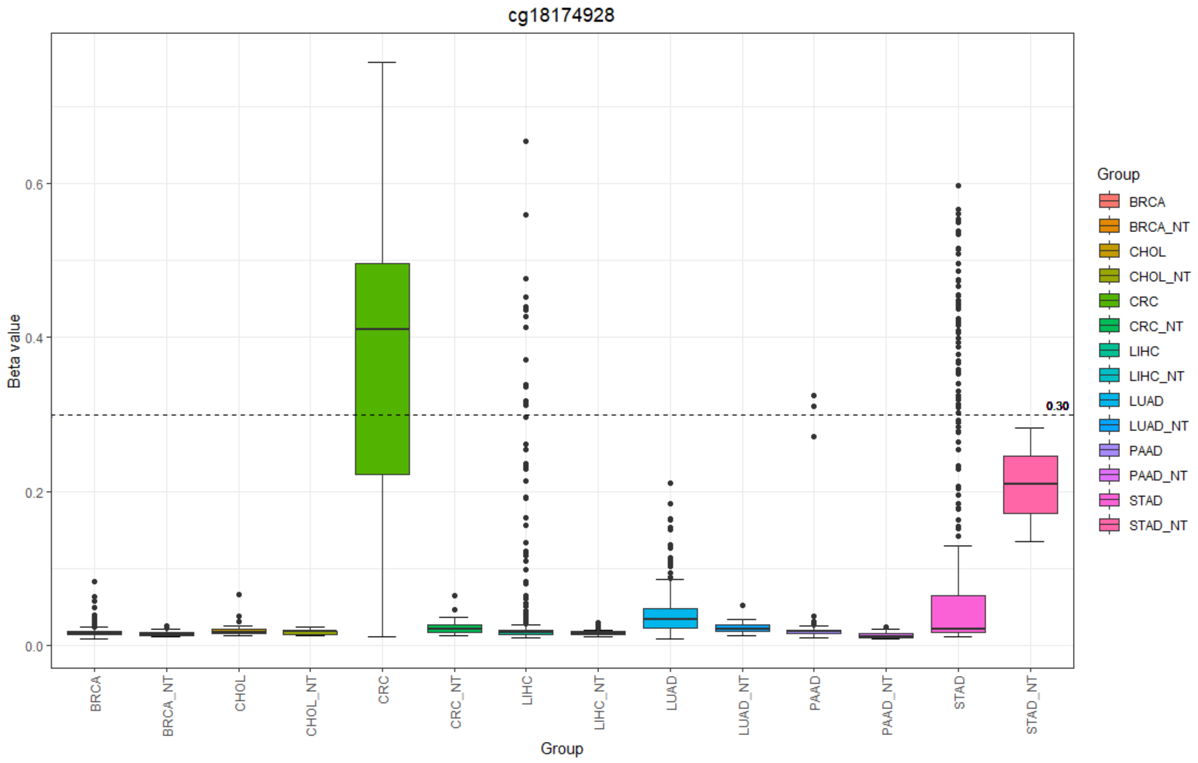

cg18174928
Beta value
0.0
0.2
0.4
0.6
0.30
Group
BRCA
BRCA_NT
CHOL
CHOL_NT
CRC
CRC_NT
LIHC
LIHC_NT
LUAD
LUAD_NT
PAAD
PAAD_NT
STAD
STAD_NT

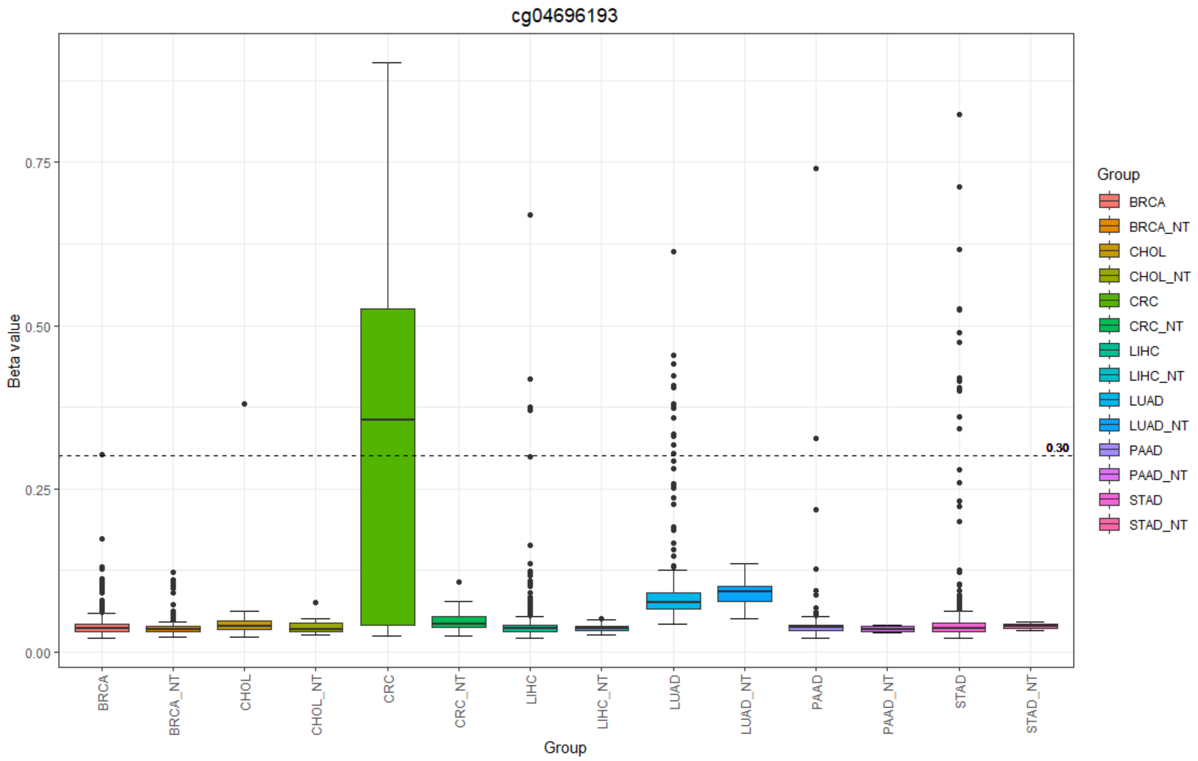

cg04696193
Beta value
0.00
0.25
0.50
0.75
0.30
Group
BRCA
BRCA_NT
CHOL
CHOL_NT
CRC
CRC_NT
LIHC
LIHC_NT
LUAD
LUAD_NT
PAAD
PAAD_NT
STAD
STAD_NT

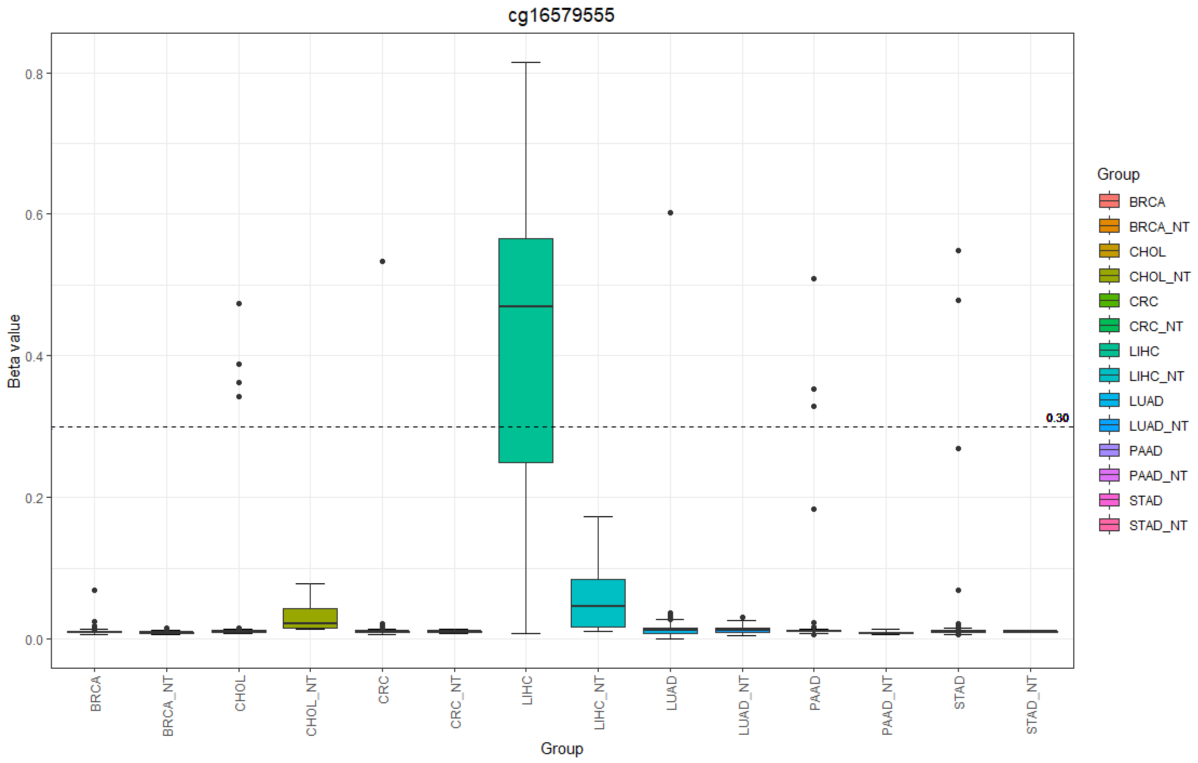
cg16579555
Beta value
0.0
0.2
0.4
0.6
0.8
0.30
Group
BRCA
BRCA_NT
CHOL
CHOL_NT
CRC
CRC_NT
LIHC
LIHC_NT
LUAD
LUAD_NT
PAAD
PAAD_NT
STAD
STAD_NT
Group
BRCA
BRCA_NT
CHOL
CHOL_NT
CRC
CRC_NT
LIHC
LIHC_NT
LUAD
LUAD_NT
PAAD
PAAD_NT
STAD
STAD_NT

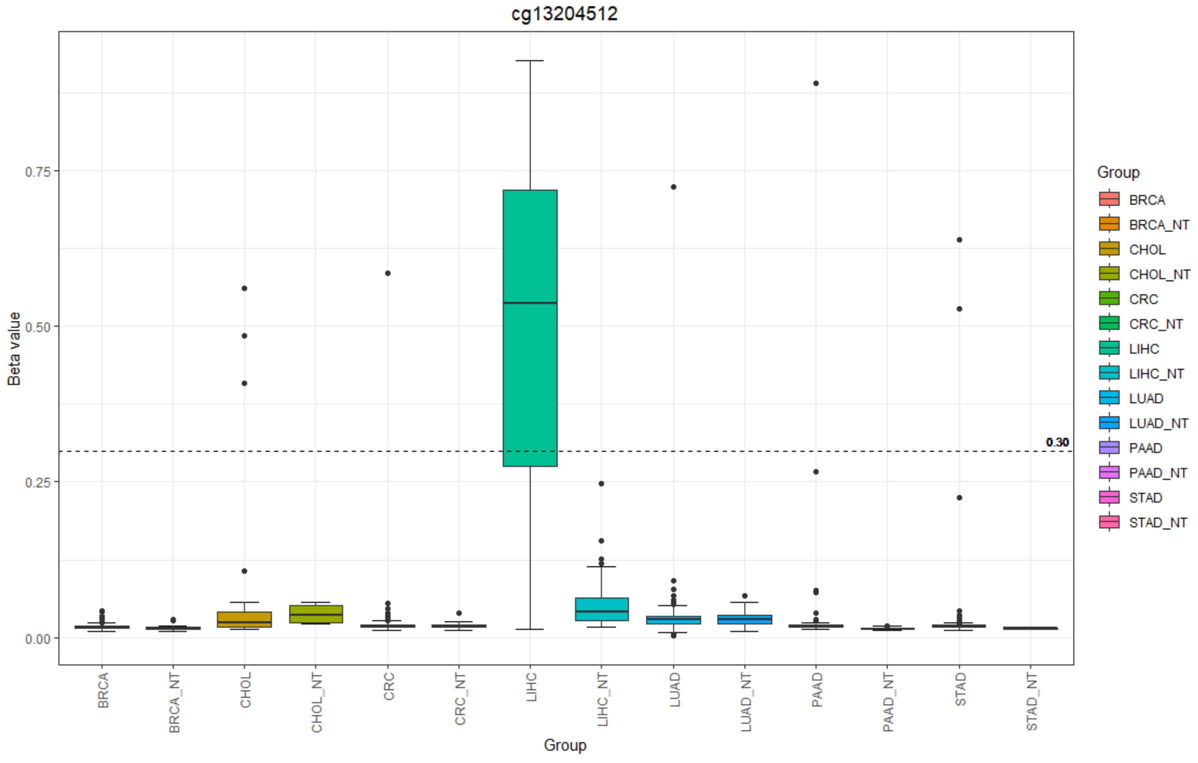
cg13204512
Beta value
0.00
0.25
0.50
0.75
0.30
Group
BRCA
BRCA_NT
CHOL
CHOL_NT
CRC
CRC_NT
LIHC
LIHC_NT
LUAD
LUAD_NT
PAAD
PAAD_NT
STAD
STAD_NT
Group
BRCA
BRCA_NT
CHOL
CHOL_NT
CRC
CRC_NT
LIHC
LIHC_NT
LUAD
LUAD_NT
PAAD
PAAD_NT
STAD
STAD_NT

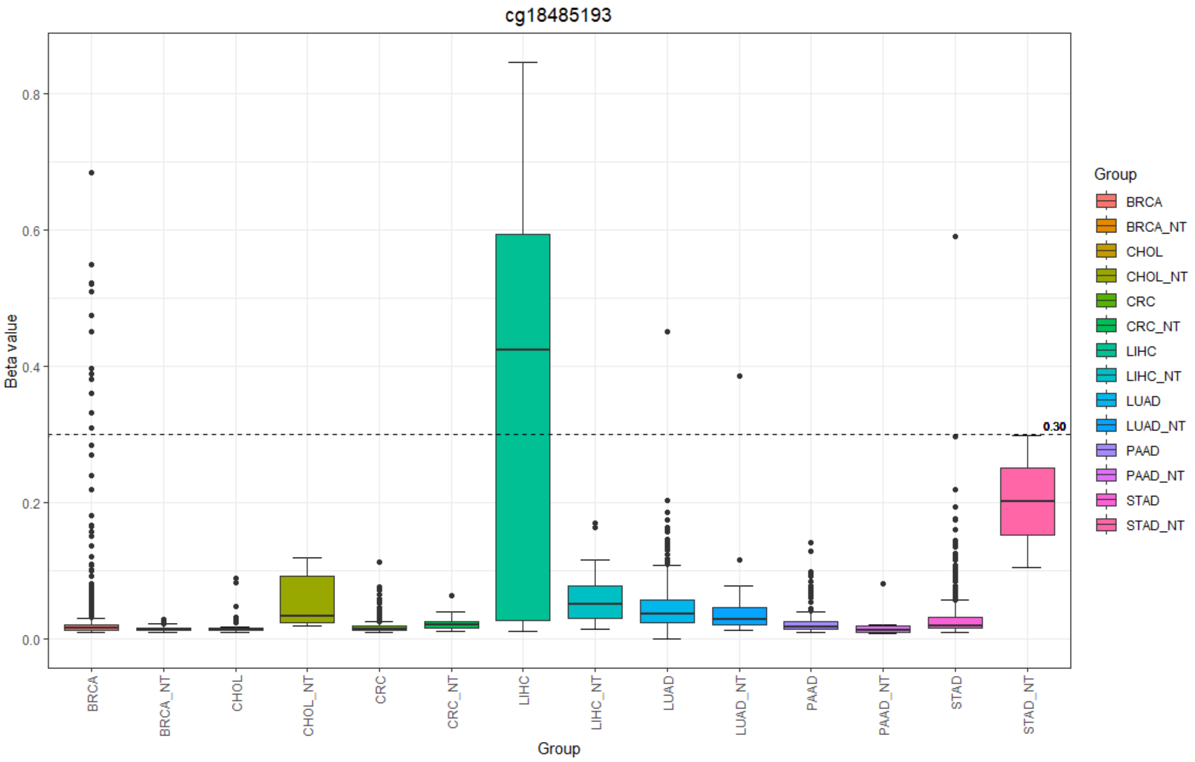
cg18485193
Beta value
0.0
0.2
0.4
0.6
0.8
0.30
BRCA
BRCA_NT
CHOL
CHOL_NT
CRC
CRC_NT
LIHC
LIHC_NT
LUAD
LUAD_NT
PAAD
PAAD_NT
STAD
STAD_NT
Group

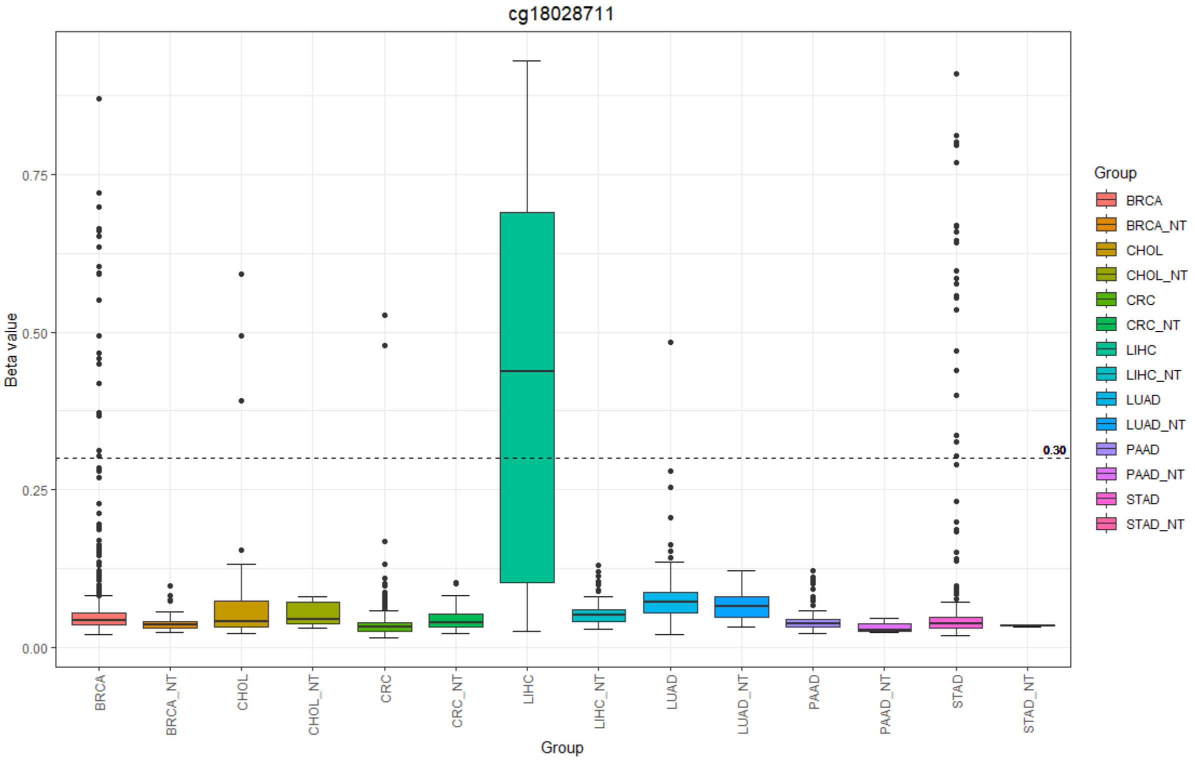
cg18028711
Beta value
0.00
0.25
0.50
0.75
0.30
BRCA
BRCA_NT
CHOL
CHOL_NT
CRC
CRC_NT
LIHC
LIHC_NT
LUAD
LUAD_NT
PAAD
PAAD_NT
STAD
STAD_NT
Group

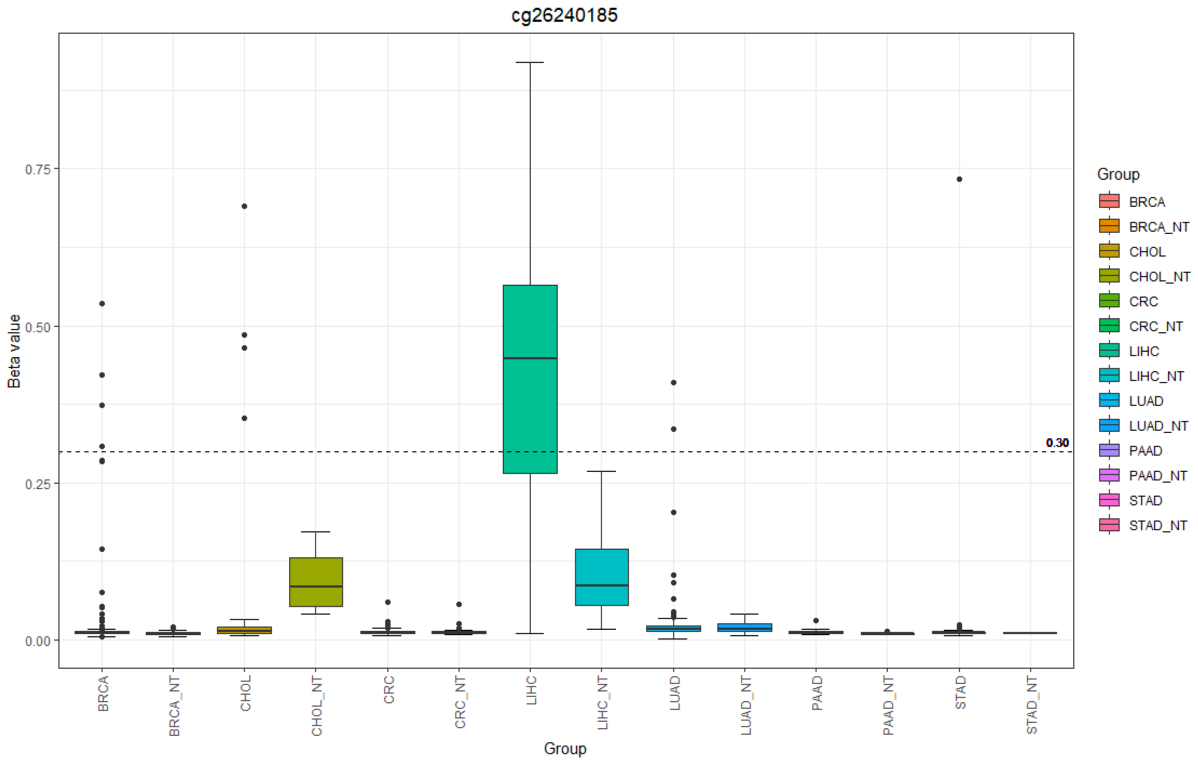
cg26240185
Beta value
0.00
0.25
0.50
0.75
0.30
Group
BRCA
BRCA_NT
CHOL
CHOL_NT
CRC
CRC_NT
LIHC
LIHC_NT
LUAD
LUAD_NT
PAAD
PAAD_NT
STAD
STAD_NT

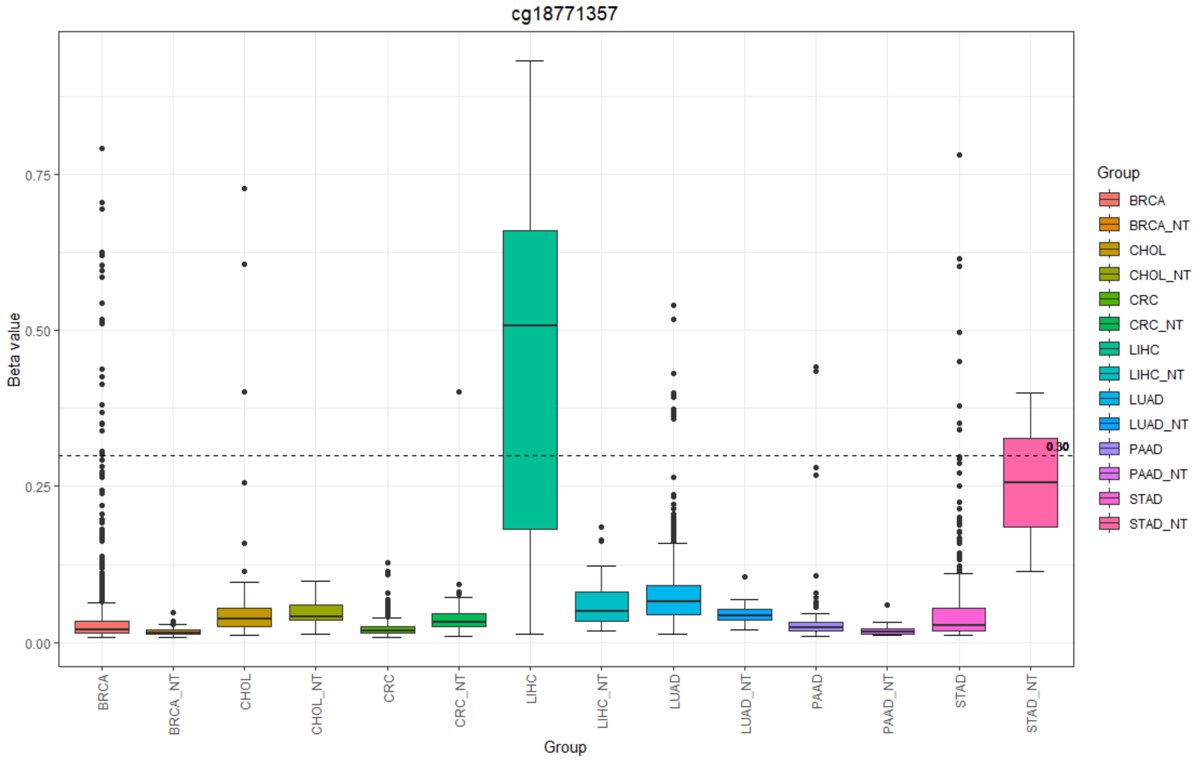
cg18771357
Beta value
0.00
0.25
0.50
0.75
0.30
Group
BRCA
BRCA_NT
CHOL
CHOL_NT
CRC
CRC_NT
LIHC
LIHC_NT
LUAD
LUAD_NT
PAAD
PAAD_NT
STAD
STAD_NT

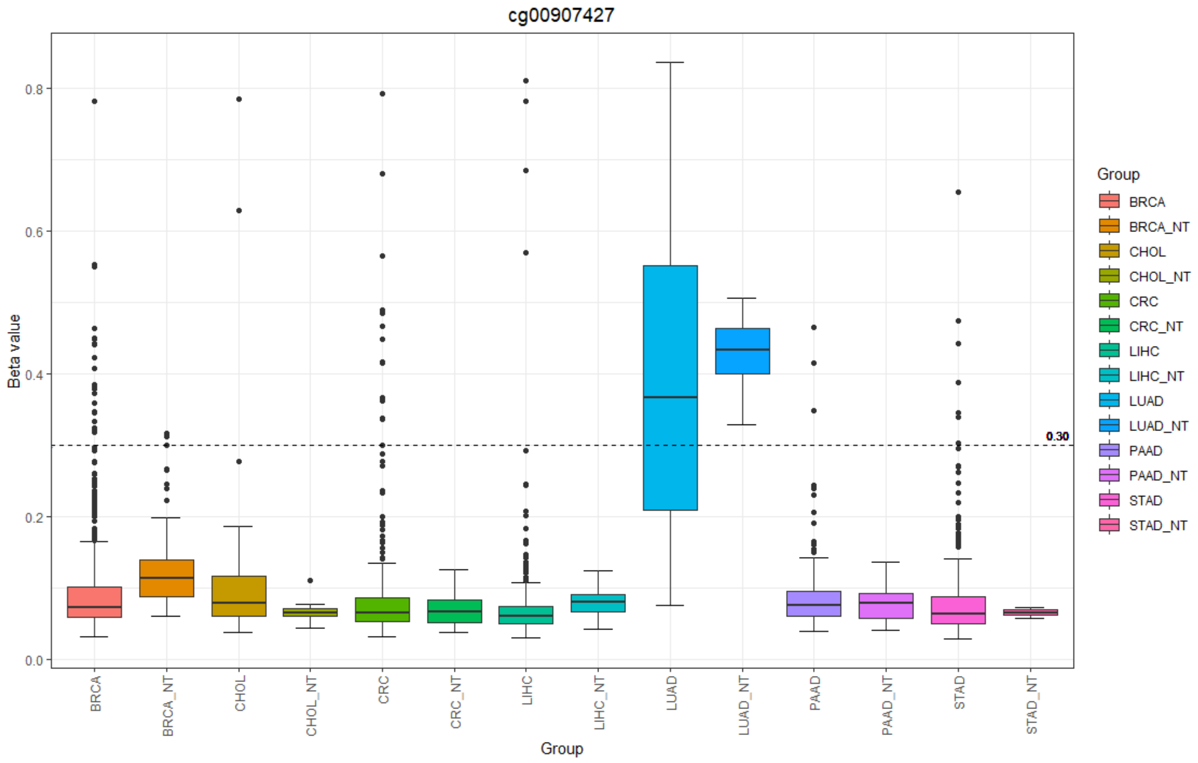
cg00907427
Beta value
0.0
0.2
0.4
0.6
0.8
0.30
Group
BRCA
BRCA_NT
CHOL
CHOL_NT
CRC
CRC_NT
LIHC
LIHC_NT
LUAD
LUAD_NT
PAAD
PAAD_NT
STAD
STAD_NT

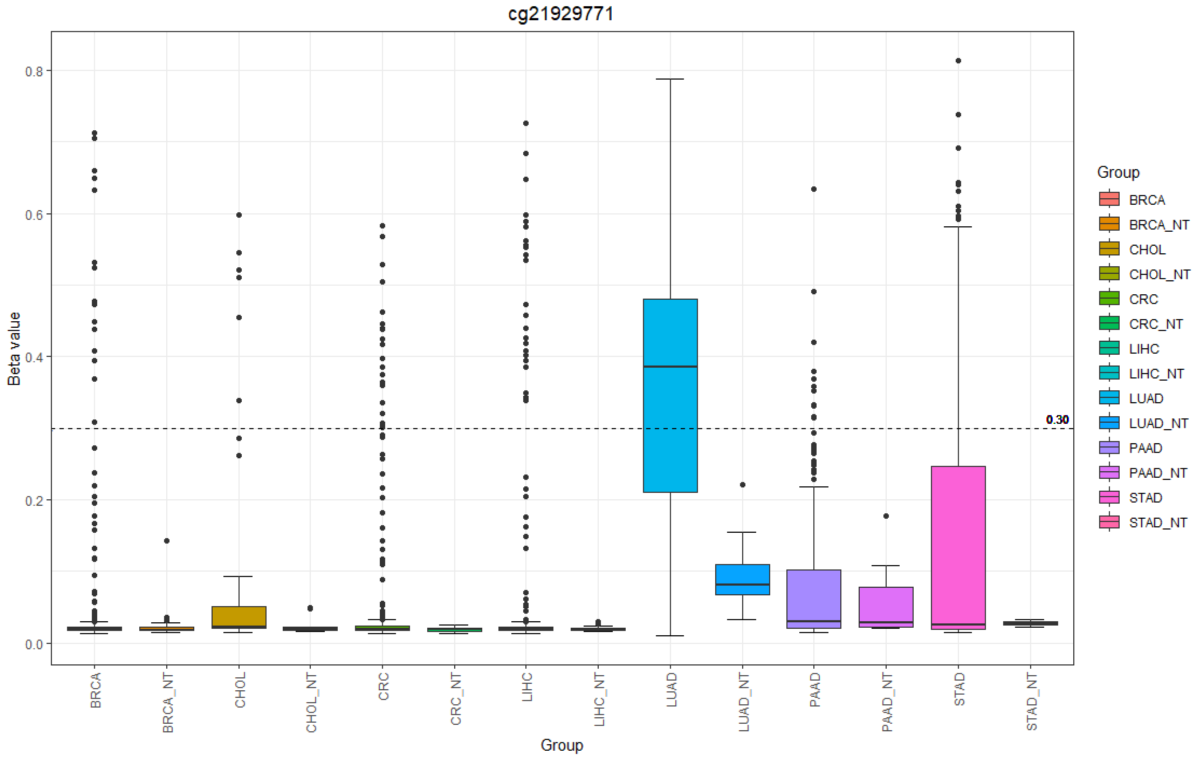
cg21929771
Beta value
0.0
0.2
0.4
0.6
0.8
0.30
Group
BRCA
BRCA_NT
CHOL
CHOL_NT
CRC
CRC_NT
LIHC
LIHC_NT
LUAD
LUAD_NT
PAAD
PAAD_NT
STAD
STAD_NT

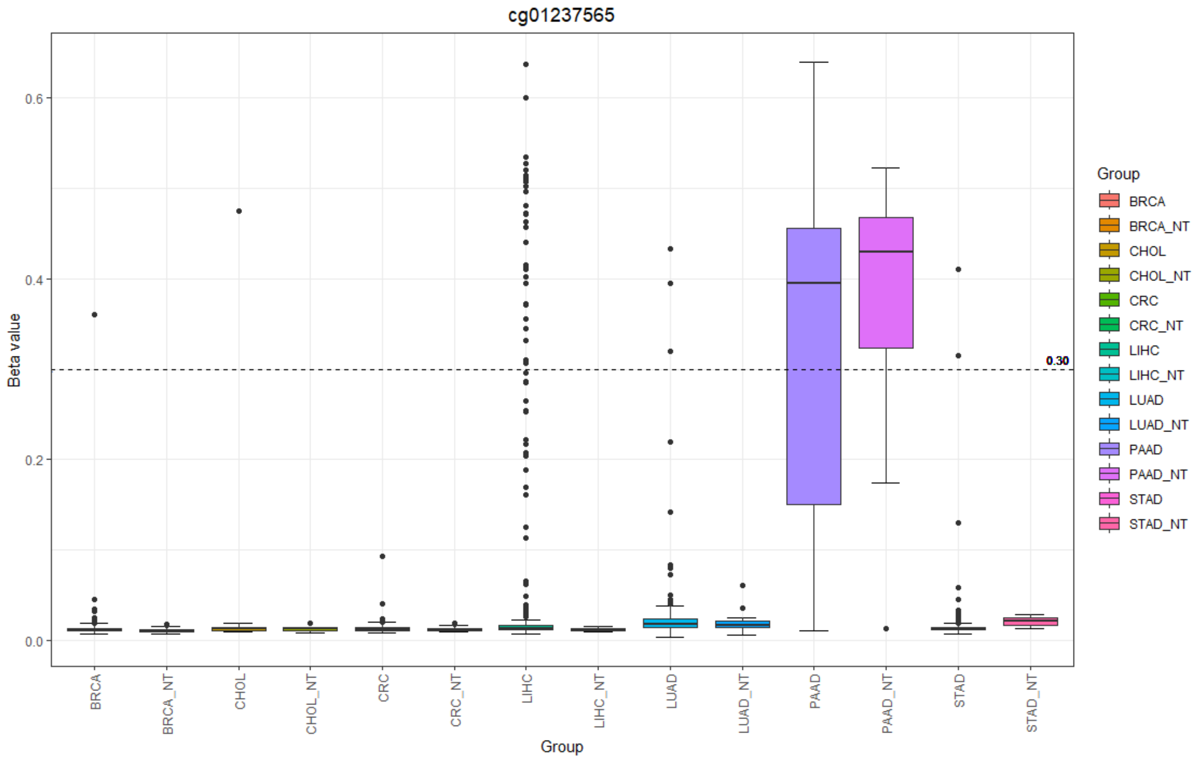
cg01237565
Beta value
0.0
0.2
0.4
0.6
0.30
Group
BRCA
BRCA_NT
CHOL
CHOL_NT
CRC
CRC_NT
LIHC
LIHC_NT
LUAD
LUAD_NT
PAAD
PAAD_NT
STAD
STAD_NT

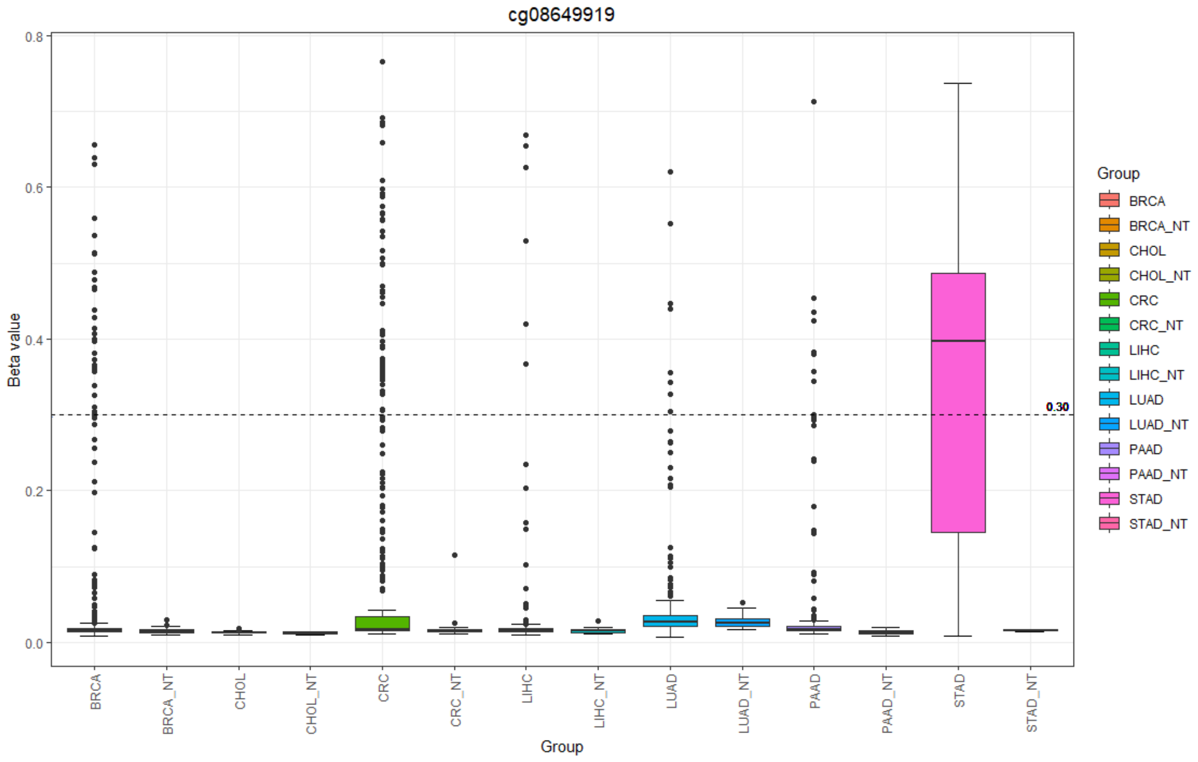
cg08649919
Beta value
0.0
0.2
0.4
0.6
0.8
0.30
Group
BRCA
BRCA_NT
CHOL
CHOL_NT
CRC
CRC_NT
LIHC
LIHC_NT
LUAD
LUAD_NT
PAAD
PAAD_NT
STAD
STAD_NT

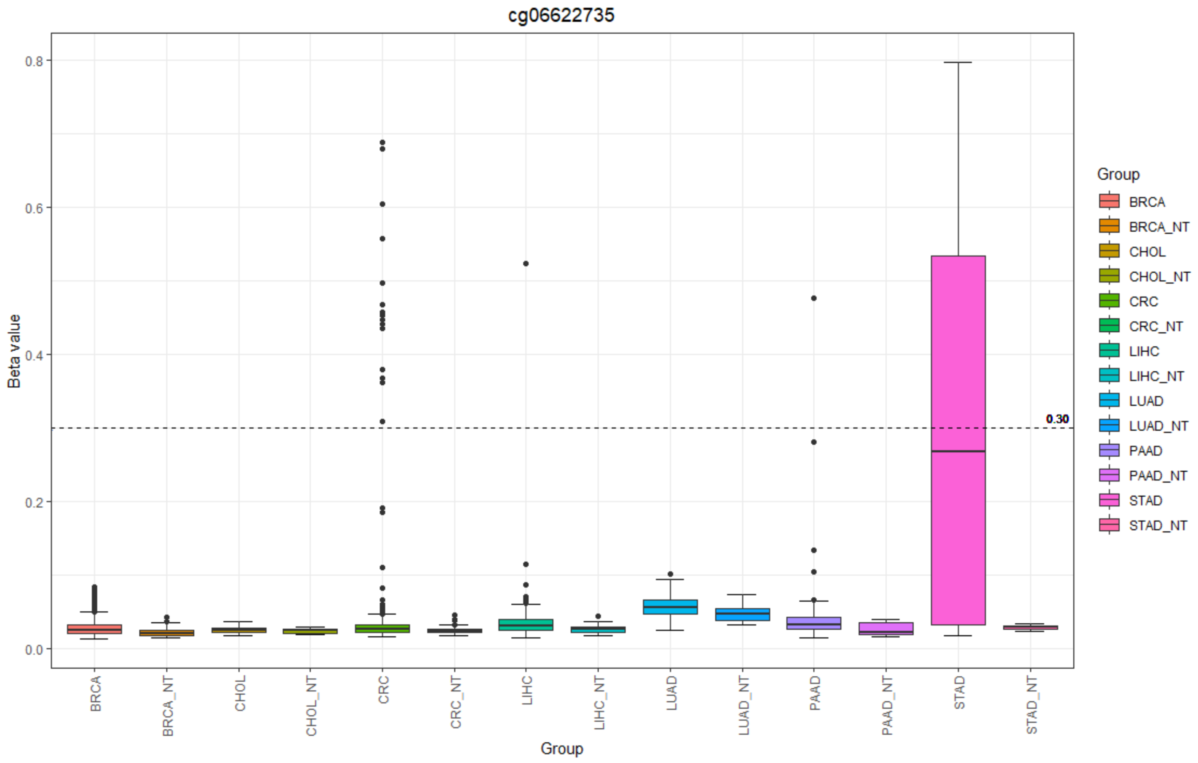
cg06622735
Beta value
0.0
0.2
0.4
0.6
0.8
0.30
Group
BRCA
BRCA_NT
CHOL
CHOL_NT
CRC
CRC_NT
LIHC
LIHC_NT
LUAD
LUAD_NT
PAAD
PAAD_NT
STAD
STAD_NT

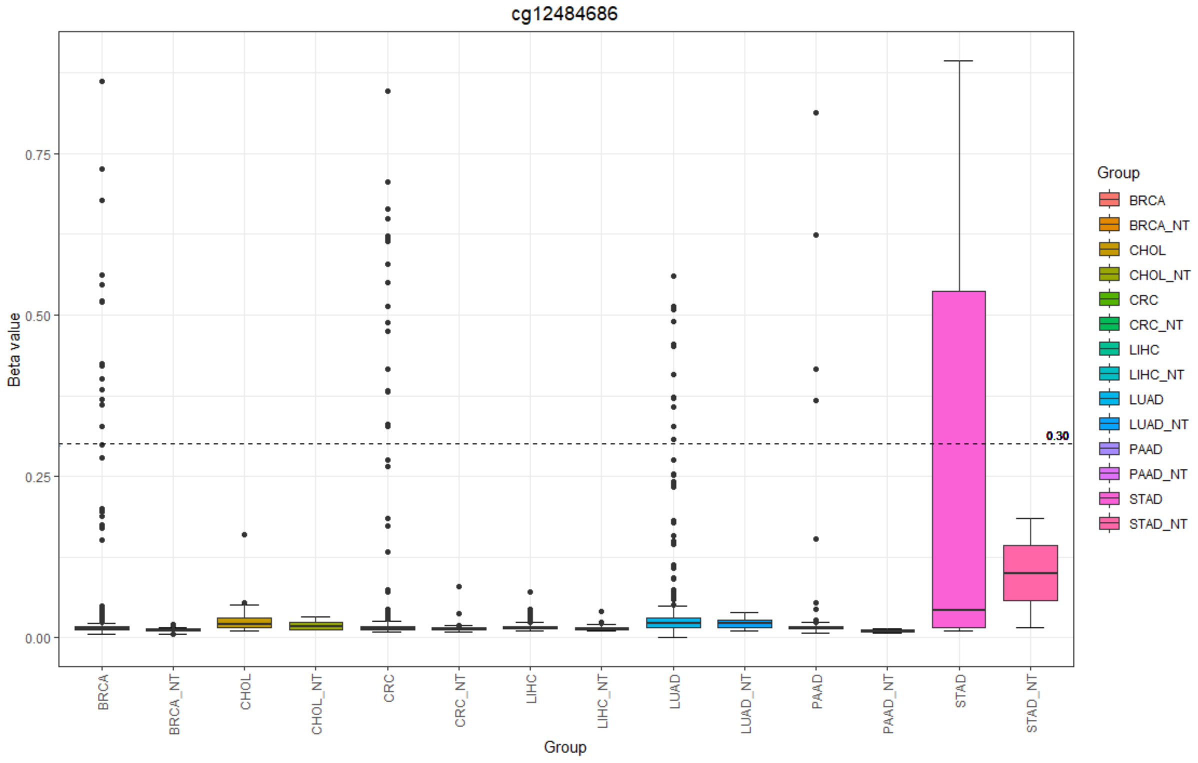
cg12484686
Beta value
0.00
0.25
0.50
0.75
0.30
Group
BRCA
BRCA_NT
CHOL
CHOL_NT
CRC
CRC_NT
LIHC
LIHC_NT
LUAD
LUAD_NT
PAAD
PAAD_NT
STAD
STAD_NT

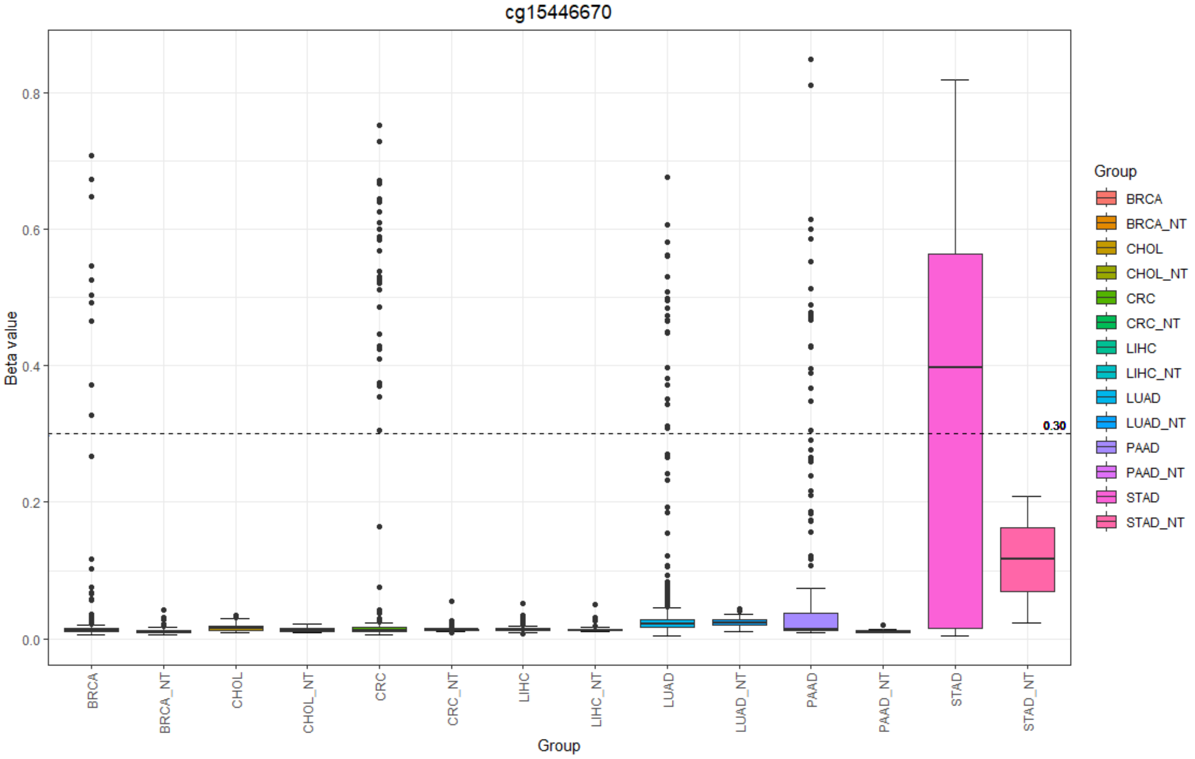

cg15446670
Beta value
0.0
0.2
0.4
0.6
0.8
0.30
BRCA
BRCA_NT
CHOL
CHOL_NT
CRC
CRC_NT
LIHC
LIHC_NT
LUAD
LUAD_NT
PAAD
PAAD_NT
STAD
STAD_NT
Group

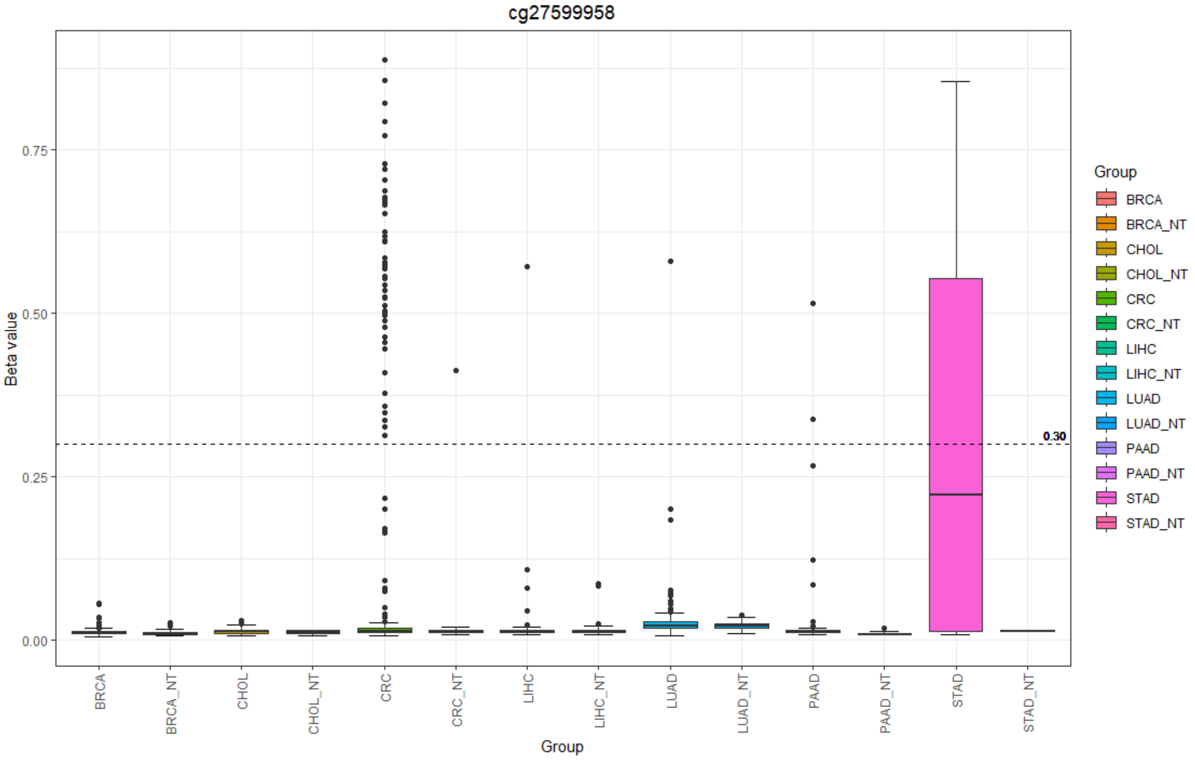

cg27599958
Beta value
0.00
0.25
0.50
0.75
0.30
BRCA
BRCA_NT
CHOL
CHOL_NT
CRC
CRC_NT
LIHC
LIHC_NT
LUAD
LUAD_NT
PAAD
PAAD_NT
STAD
STAD_NT
Group

Fig. S3: Boxplots showing the distribution of beta values of selected probes from the clustered approach across the included tumor samples and the normal tissue samples.

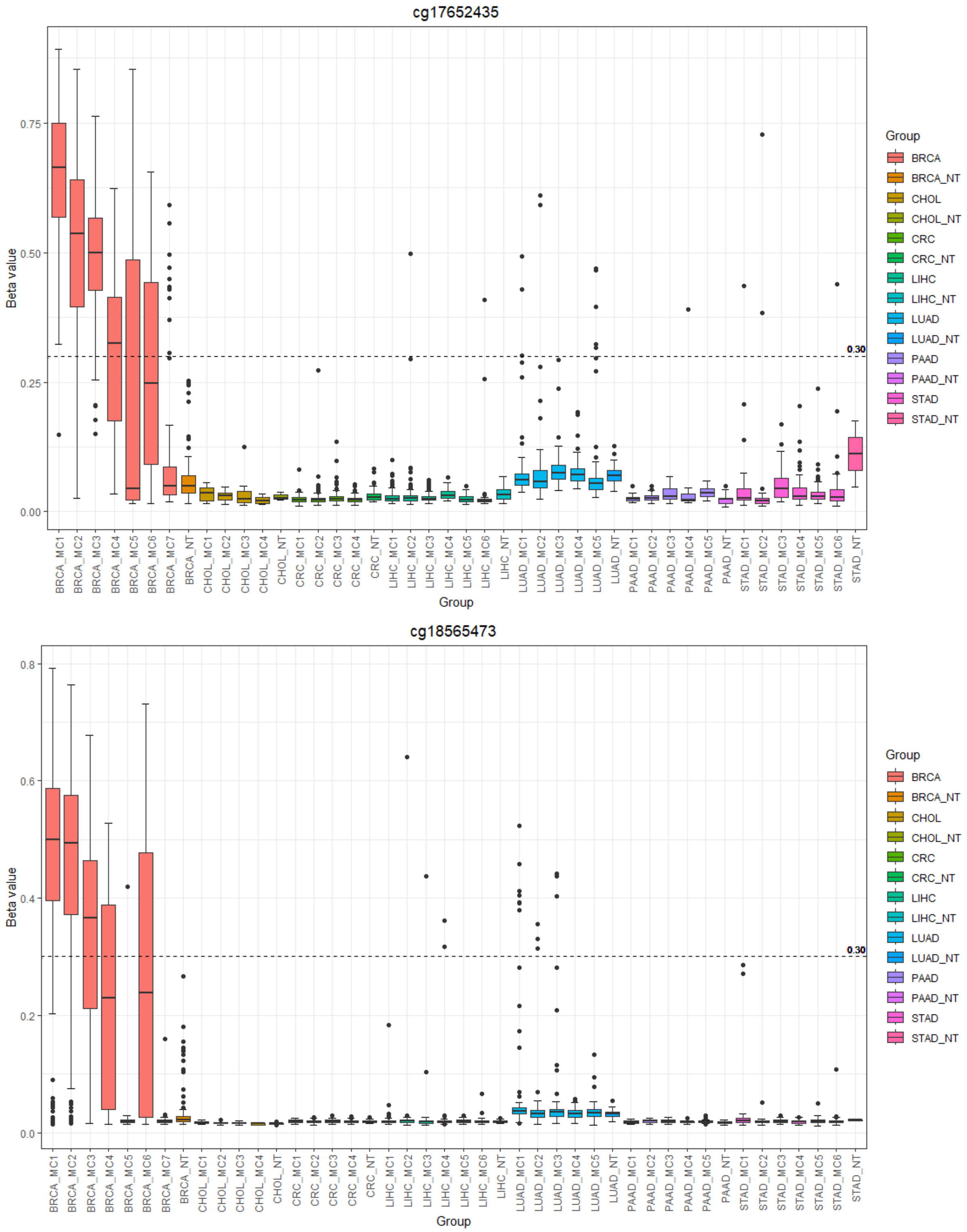

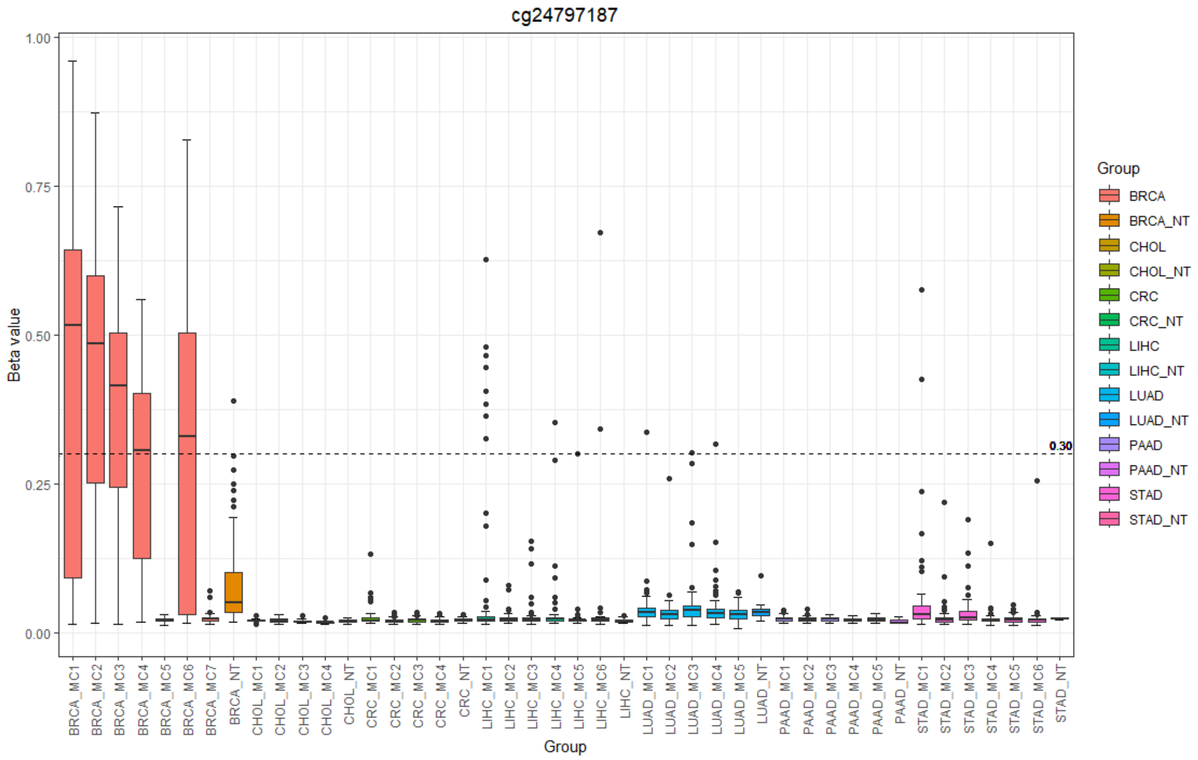
cg24797187
Beta value
Group
0.30
Group
BRCA
BRCA_NT
CHOL
CHOL_NT
CRC
CRC_NT
LIHC
LIHC_NT
LUAD
LUAD_NT
PAAD
PAAD_NT
STAD
STAD_NT

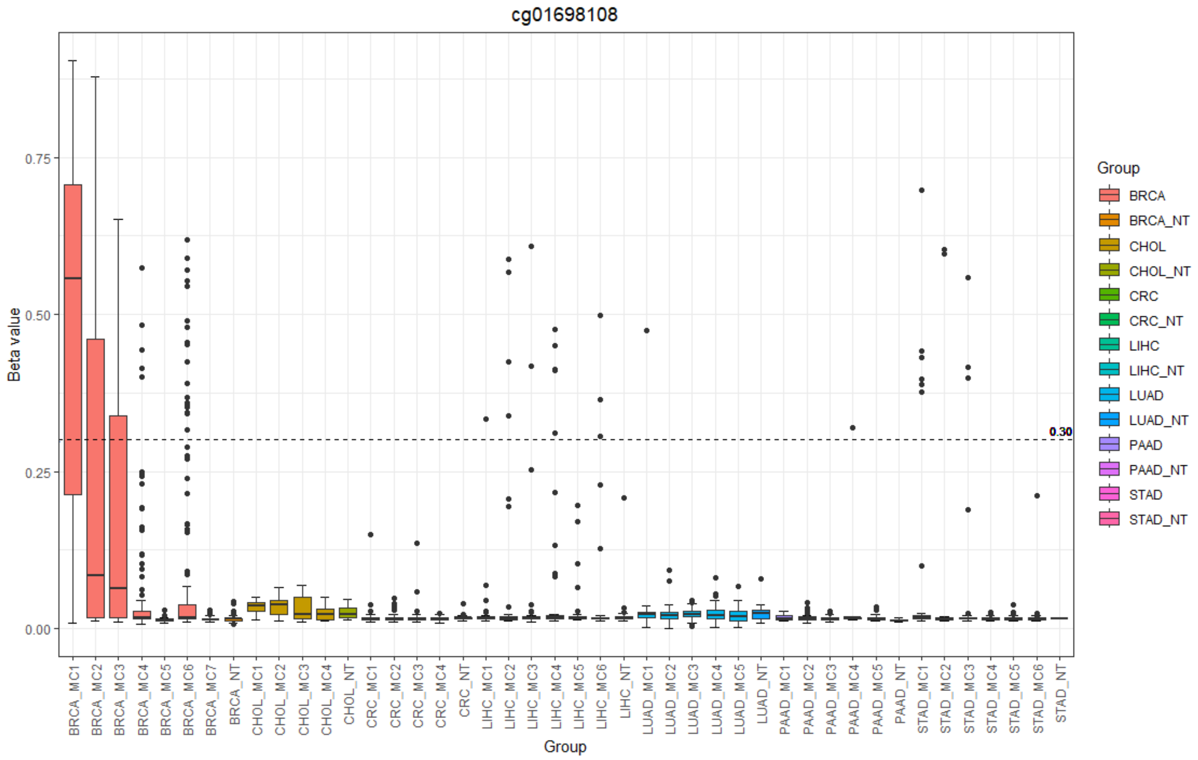
cg01698108
Beta value
Group
0.30
Group
BRCA
BRCA_NT
CHOL
CHOL_NT
CRC
CRC_NT
LIHC
LIHC_NT
LUAD
LUAD_NT
PAAD
PAAD_NT
STAD
STAD_NT

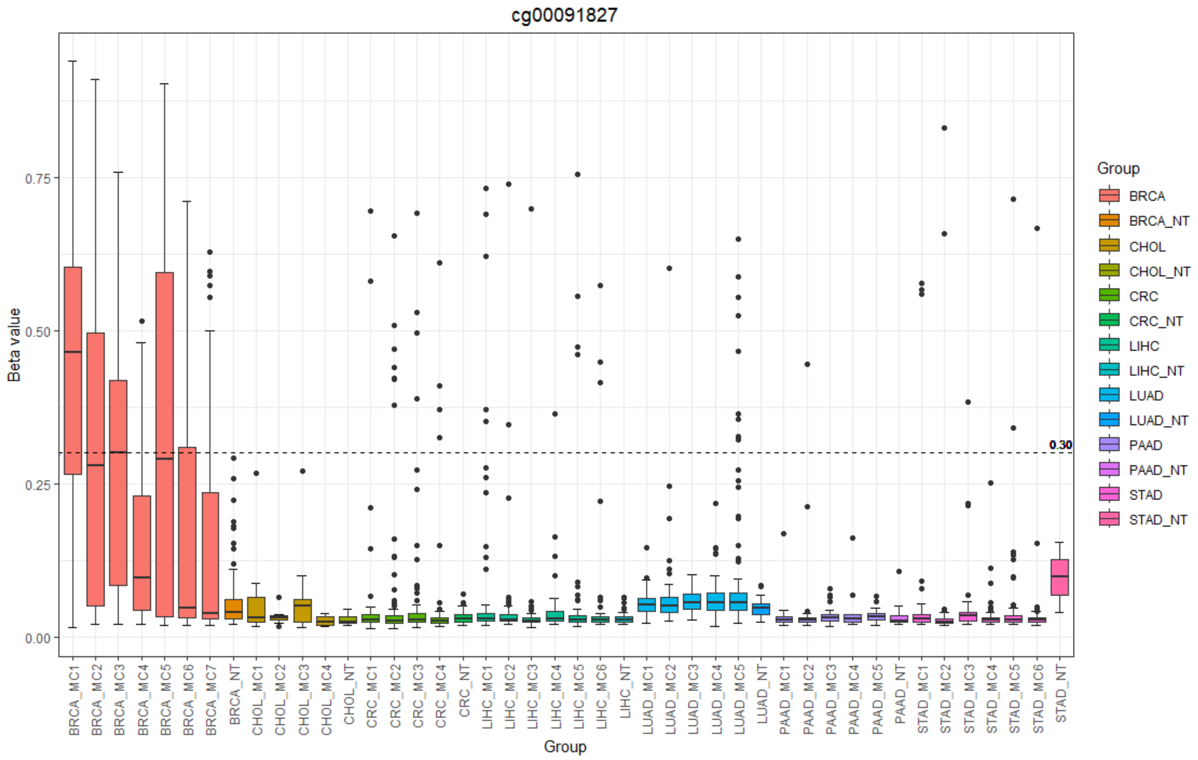
cg00091827
Beta value
0.00
0.25
0.50
0.75
0.30
Group
BRCA_MC1
BRCA_MC2
BRCA_MC3
BRCA_MC4
BRCA_MC5
BRCA_MC6
BRCA_MC7
BRCA_NT
CHOL_MC1
CHOL_MC2
CHOL_MC3
CHOL_MC4
CHOL_NT
CRC_MC1
CRC_MC2
CRC_MC3
CRC_MC4
CRC_NT
LIHC_MC1
LIHC_MC2
LIHC_MC3
LIHC_MC4
LIHC_MC5
LIHC_MC6
LIHC_NT
LUAD_MC1
LUAD_MC2
LUAD_MC3
LUAD_MC4
LUAD_MC5
LUAD_NT
PAAD_MC1
PAAD_MC2
PAAD_MC3
PAAD_MC4
PAAD_MC5
PAAD_NT
STAD_MC1
STAD_MC2
STAD_MC3
STAD_MC4
STAD_MC5
STAD_MC6
STAD_NT
Group
BRCA
BRCA_NT
CHOL
CHOL_NT
CRC
CRC_NT
LIHC
LIHC_NT
LUAD
LUAD_NT
PAAD
PAAD_NT
STAD
STAD_NT

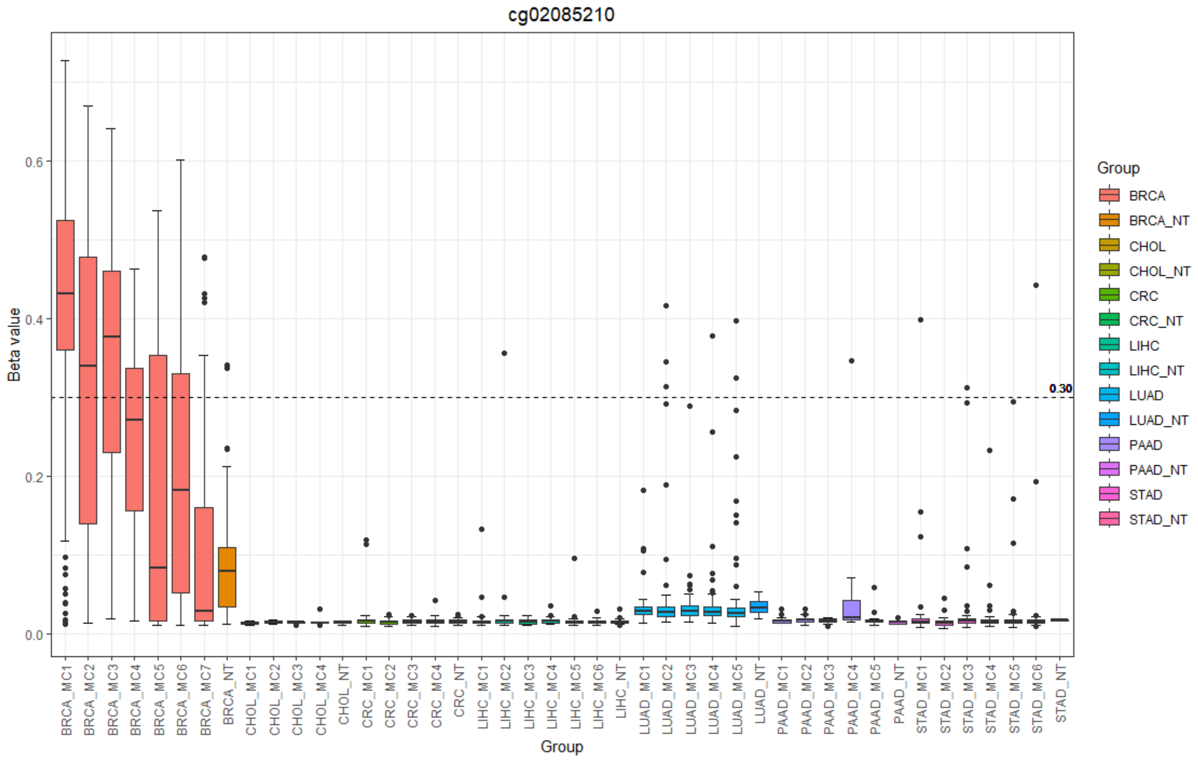
cg02085210
Beta value
0.0
0.2
0.4
0.6
0.30
Group
BRCA_MC1
BRCA_MC2
BRCA_MC3
BRCA_MC4
BRCA_MC5
BRCA_MC6
BRCA_MC7
BRCA_NT
CHOL_MC1
CHOL_MC2
CHOL_MC3
CHOL_MC4
CHOL_NT
CRC_MC1
CRC_MC2
CRC_MC3
CRC_MC4
CRC_NT
LIHC_MC1
LIHC_MC2
LIHC_MC3
LIHC_MC4
LIHC_MC5
LIHC_MC6
LIHC_NT
LUAD_MC1
LUAD_MC2
LUAD_MC3
LUAD_MC4
LUAD_MC5
LUAD_NT
PAAD_MC1
PAAD_MC2
PAAD_MC3
PAAD_MC4
PAAD_MC5
PAAD_NT
STAD_MC1
STAD_MC2
STAD_MC3
STAD_MC4
STAD_MC5
STAD_MC6
STAD_NT
Group
BRCA
BRCA_NT
CHOL
CHOL_NT
CRC
CRC_NT
LIHC
LIHC_NT
LUAD
LUAD_NT
PAAD
PAAD_NT
STAD
STAD_NT

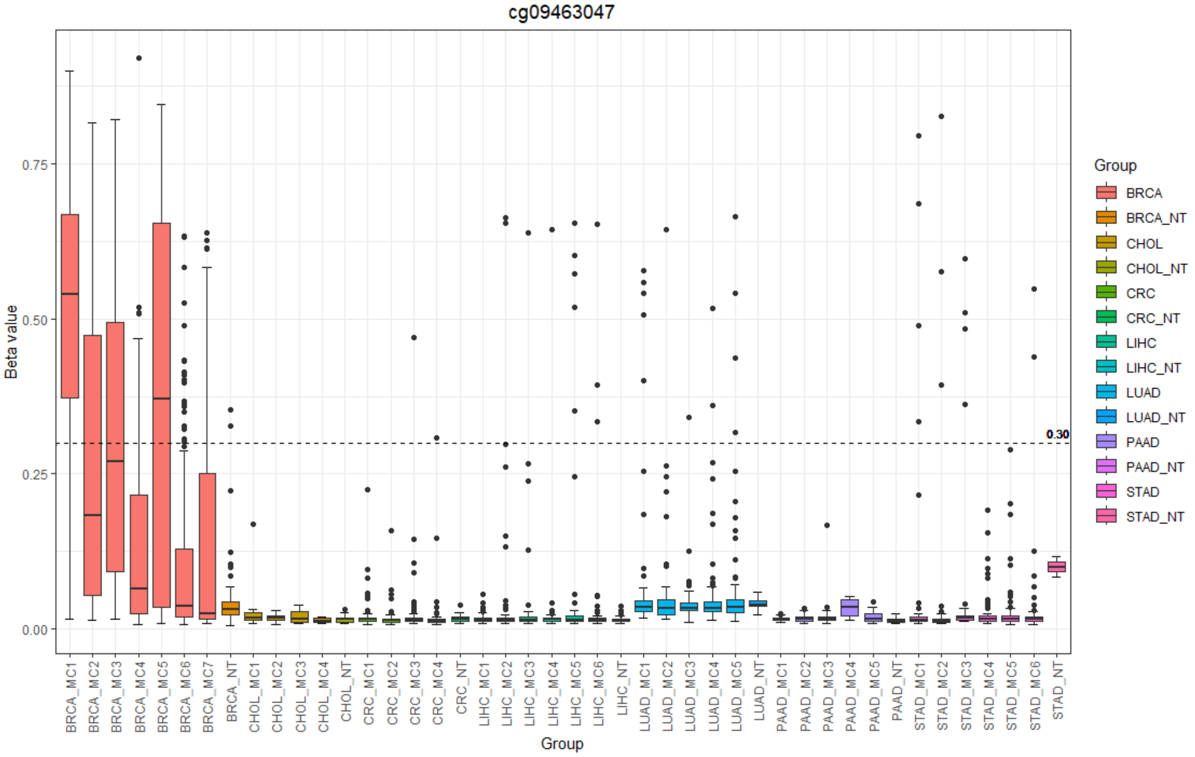

cg09463047
Beta value
0.00
0.25
0.50
0.75
0.30
Group
BRCA
BRCA_NT
CHOL
CHOL_NT
CRC
CRC_NT
LIHC
LIHC_NT
LUAD
LUAD_NT
PAAD
PAAD_NT
STAD
STAD_NT
BRCA_MC1
BRCA_MC2
BRCA_MC3
BRCA_MC4
BRCA_MC5
BRCA_MC6
BRCA_MC7
BRCA_NT
CHOL_MC1
CHOL_MC2
CHOL_MC3
CHOL_MC4
CHOL_NT
CRC_MC1
CRC_MC2
CRC_MC3
CRC_MC4
CRC_NT
LIHC_MC1
LIHC_MC2
LIHC_MC3
LIHC_MC4
LIHC_MC5
LIHC_MC6
LIHC_NT
LUAD_MC1
LUAD_MC2
LUAD_MC3
LUAD_MC4
LUAD_MC5
LUAD_NT
PAAD_MC1
PAAD_MC2
PAAD_MC3
PAAD_MC4
PAAD_MC5
PAAD_NT
STAD_MC1
STAD_MC2
STAD_MC3
STAD_MC4
STAD_MC5
STAD_MC6
STAD_NT
Group

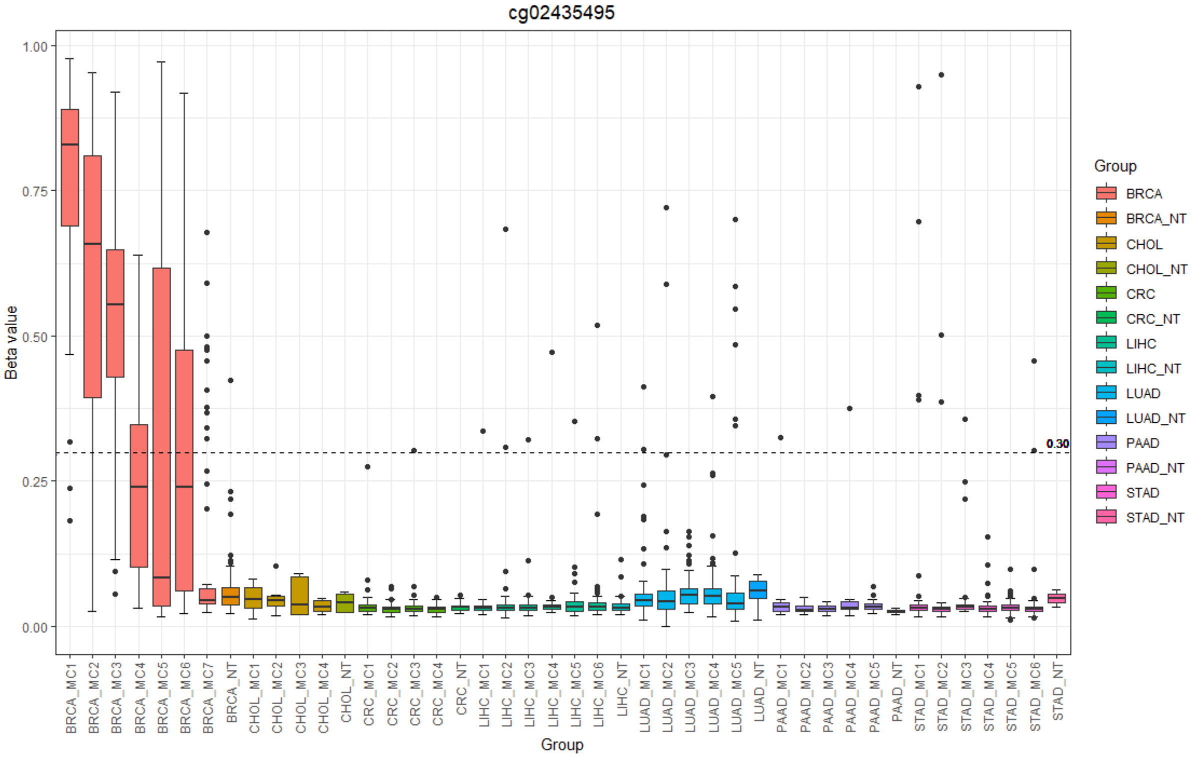

cg02435495
Beta value
0.00
0.25
0.50
0.75
1.00
0.30
Group
BRCA
BRCA_NT
CHOL
CHOL_NT
CRC
CRC_NT
LIHC
LIHC_NT
LUAD
LUAD_NT
PAAD
PAAD_NT
STAD
STAD_NT
BRCA_MC1
BRCA_MC2
BRCA_MC3
BRCA_MC4
BRCA_MC5
BRCA_MC6
BRCA_MC7
BRCA_NT
CHOL_MC1
CHOL_MC2
CHOL_MC3
CHOL_MC4
CHOL_NT
CRC_MC1
CRC_MC2
CRC_MC3
CRC_MC4
CRC_NT
LIHC_MC1
LIHC_MC2
LIHC_MC3
LIHC_MC4
LIHC_MC5
LIHC_MC6
LIHC_NT
LUAD_MC1
LUAD_MC2
LUAD_MC3
LUAD_MC4
LUAD_MC5
LUAD_NT
PAAD_MC1
PAAD_MC2
PAAD_MC3
PAAD_MC4
PAAD_MC5
PAAD_NT
STAD_MC1
STAD_MC2
STAD_MC3
STAD_MC4
STAD_MC5
STAD_MC6
STAD_NT
Group

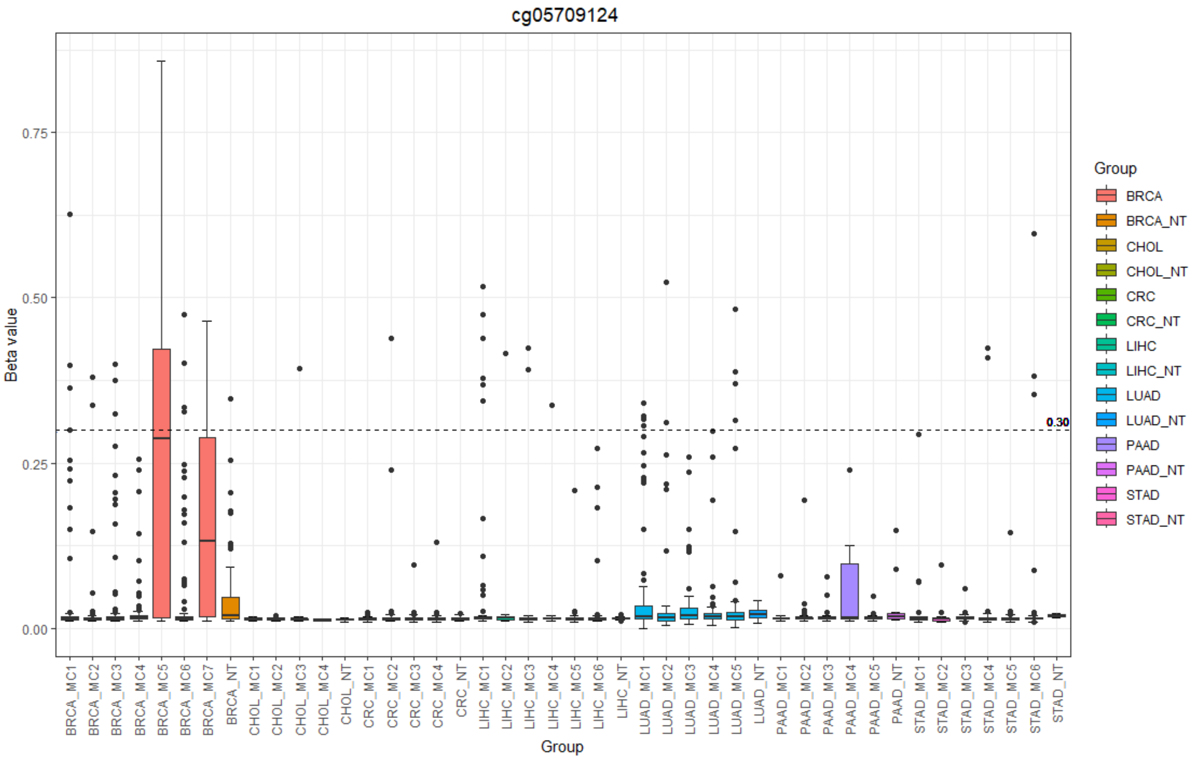
cg05709124
Beta value
0.00
0.25
0.50
0.75
0.30
BRCA_MC1
BRCA_MC2
BRCA_MC3
BRCA_MC4
BRCA_MC5
BRCA_MC6
BRCA_MC7
BRCA_NT
CHOL_MC1
CHOL_MC2
CHOL_MC3
CHOL_MC4
CHOL_NT
CRC_MC1
CRC_MC2
CRC_MC3
CRC_MC4
CRC_NT
LIHC_MC1
LIHC_MC2
LIHC_MC3
LIHC_MC4
LIHC_MC5
LIHC_MC6
LIHC_NT
LUAD_MC1
LUAD_MC2
LUAD_MC3
LUAD_MC4
LUAD_MC5
LUAD_NT
PAAD_MC1
PAAD_MC2
PAAD_MC3
PAAD_MC4
PAAD_MC5
PAAD_NT
STAD_MC1
STAD_MC2
STAD_MC3
STAD_MC4
STAD_MC5
STAD_MC6
STAD_NT
Group
Group
BRCA
BRCA_NT
CHOL
CHOL_NT
CRC
CRC_NT
LIHC
LIHC_NT
LUAD
LUAD_NT
PAAD
PAAD_NT
STAD
STAD_NT

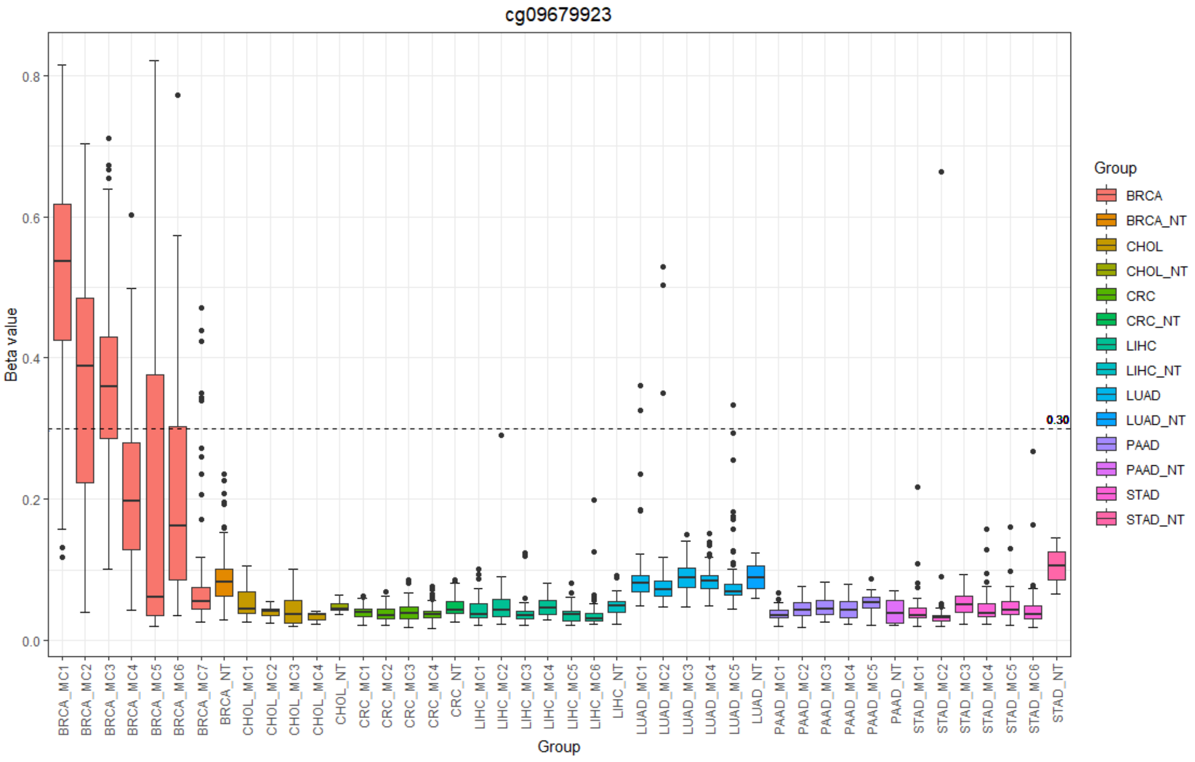
cg09679923
Beta value
0.0
0.2
0.4
0.6
0.8
0.30
BRCA_MC1
BRCA_MC2
BRCA_MC3
BRCA_MC4
BRCA_MC5
BRCA_MC6
BRCA_MC7
BRCA_NT
CHOL_MC1
CHOL_MC2
CHOL_MC3
CHOL_MC4
CHOL_NT
CRC_MC1
CRC_MC2
CRC_MC3
CRC_MC4
CRC_NT
LIHC_MC1
LIHC_MC2
LIHC_MC3
LIHC_MC4
LIHC_MC5
LIHC_MC6
LIHC_NT
LUAD_MC1
LUAD_MC2
LUAD_MC3
LUAD_MC4
LUAD_MC5
LUAD_NT
PAAD_MC1
PAAD_MC2
PAAD_MC3
PAAD_MC4
PAAD_MC5
PAAD_NT
STAD_MC1
STAD_MC2
STAD_MC3
STAD_MC4
STAD_MC5
STAD_MC6
STAD_NT
Group
Group
BRCA
BRCA_NT
CHOL
CHOL_NT
CRC
CRC_NT
LIHC
LIHC_NT
LUAD
LUAD_NT
PAAD
PAAD_NT
STAD
STAD_NT

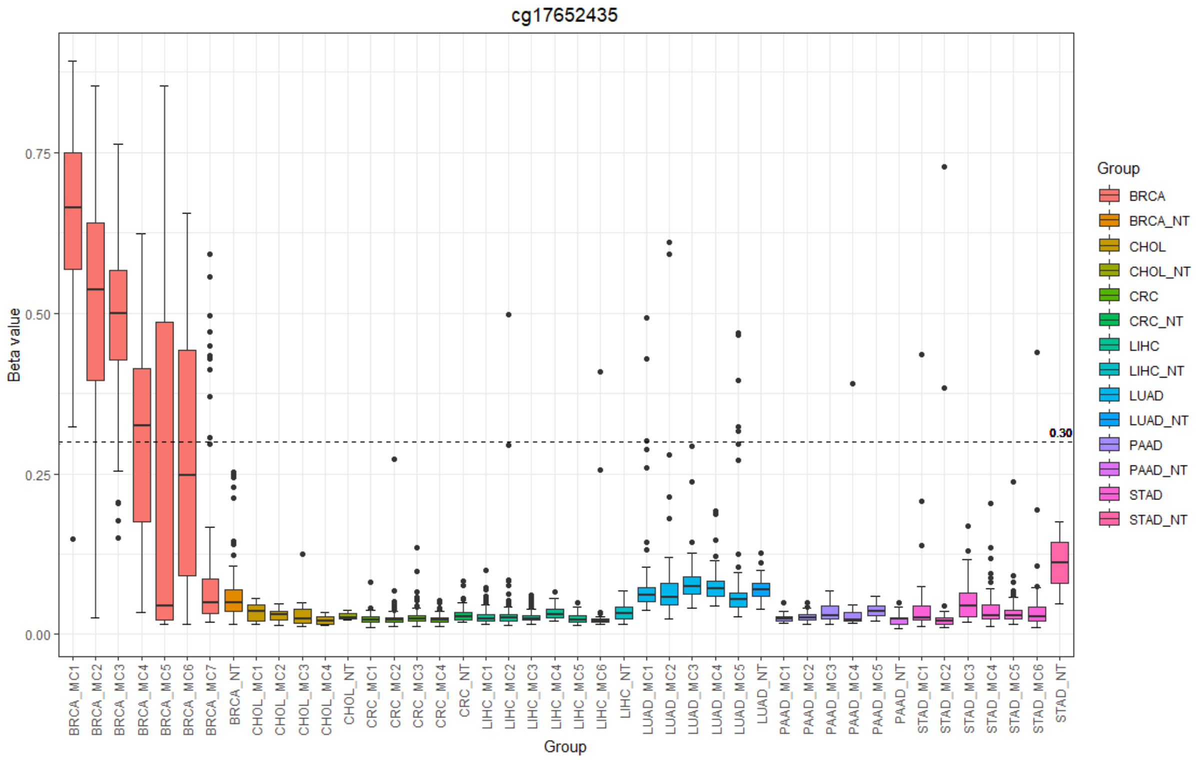
cg17652435
Beta value
0.00
0.25
0.50
0.75
0.30
Group
BRCA_MC1
BRCA_MC2
BRCA_MC3
BRCA_MC4
BRCA_MC5
BRCA_MC6
BRCA_MC7
BRCA_NT
CHOL_MC1
CHOL_MC2
CHOL_MC3
CHOL_MC4
CHOL_NT
CRC_MC1
CRC_MC2
CRC_MC3
CRC_MC4
CRC_NT
LIHC_MC1
LIHC_MC2
LIHC_MC3
LIHC_MC4
LIHC_MC5
LIHC_MC6
LIHC_NT
LUAD_MC1
LUAD_MC2
LUAD_MC3
LUAD_MC4
LUAD_MC5
LUAD_NT
PAAD_MC1
PAAD_MC2
PAAD_MC3
PAAD_MC4
PAAD_MC5
PAAD_NT
STAD_MC1
STAD_MC2
STAD_MC3
STAD_MC4
STAD_MC5
STAD_MC6
STAD_NT
Group
BRCA
BRCA_NT
CHOL
CHOL_NT
CRC
CRC_NT
LIHC
LIHC_NT
LUAD
LUAD_NT
PAAD
PAAD_NT
STAD
STAD_NT

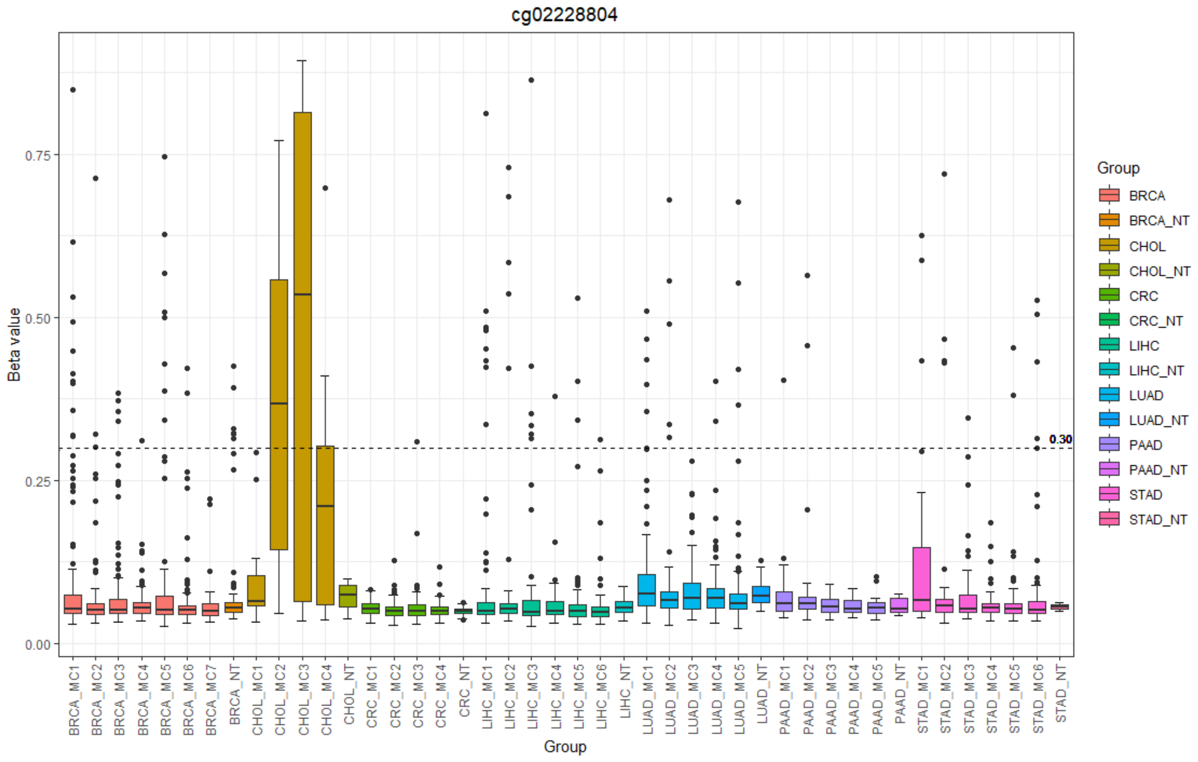
cg02228804
Beta value
0.00
0.25
0.50
0.75
0.30
Group
BRCA_MC1
BRCA_MC2
BRCA_MC3
BRCA_MC4
BRCA_MC5
BRCA_MC6
BRCA_MC7
BRCA_NT
CHOL_MC1
CHOL_MC2
CHOL_MC3
CHOL_MC4
CHOL_NT
CRC_MC1
CRC_MC2
CRC_MC3
CRC_MC4
CRC_NT
LIHC_MC1
LIHC_MC2
LIHC_MC3
LIHC_MC4
LIHC_MC5
LIHC_MC6
LIHC_NT
LUAD_MC1
LUAD_MC2
LUAD_MC3
LUAD_MC4
LUAD_MC5
LUAD_NT
PAAD_MC1
PAAD_MC2
PAAD_MC3
PAAD_MC4
PAAD_MC5
PAAD_NT
STAD_MC1
STAD_MC2
STAD_MC3
STAD_MC4
STAD_MC5
STAD_MC6
STAD_NT
Group
BRCA
BRCA_NT
CHOL
CHOL_NT
CRC
CRC_NT
LIHC
LIHC_NT
LUAD
LUAD_NT
PAAD
PAAD_NT
STAD
STAD_NT

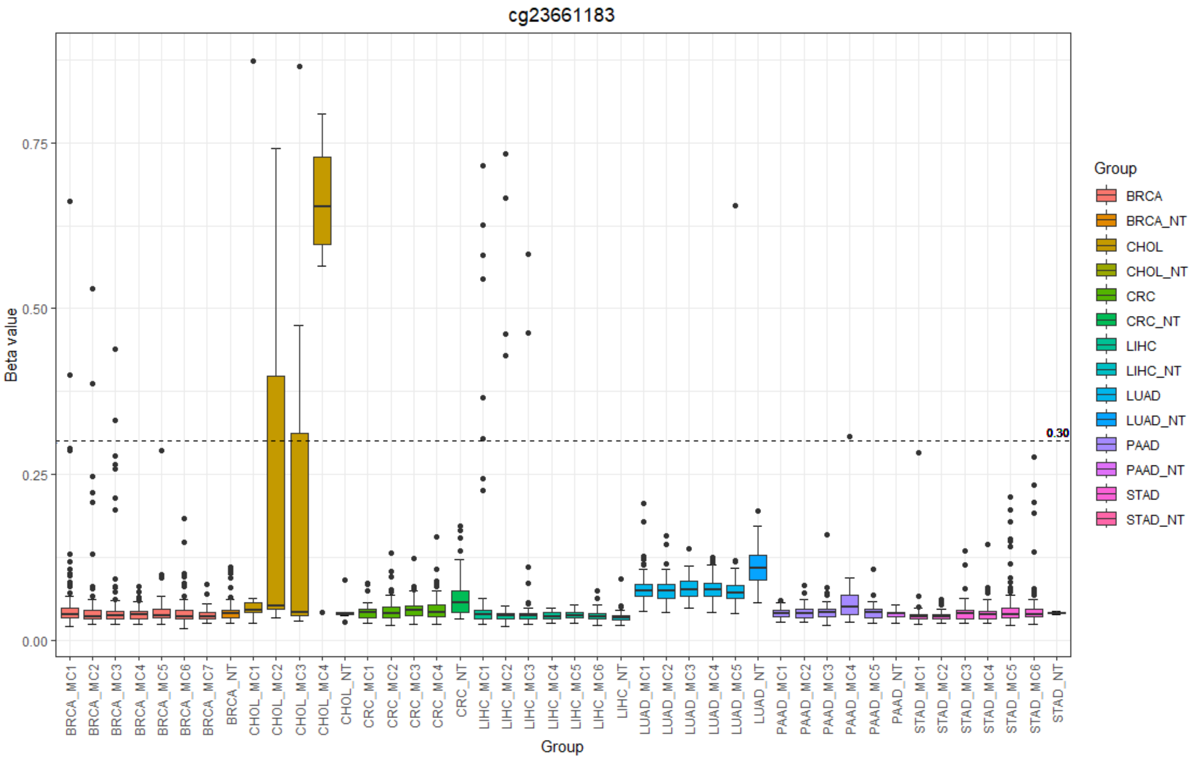

cg23661183
Beta value
Group
0.30
Group
BRCA
BRCA_NT
CHOL
CHOL_NT
CRC
CRC_NT
LIHC
LIHC_NT
LUAD
LUAD_NT
PAAD
PAAD_NT
STAD
STAD_NT

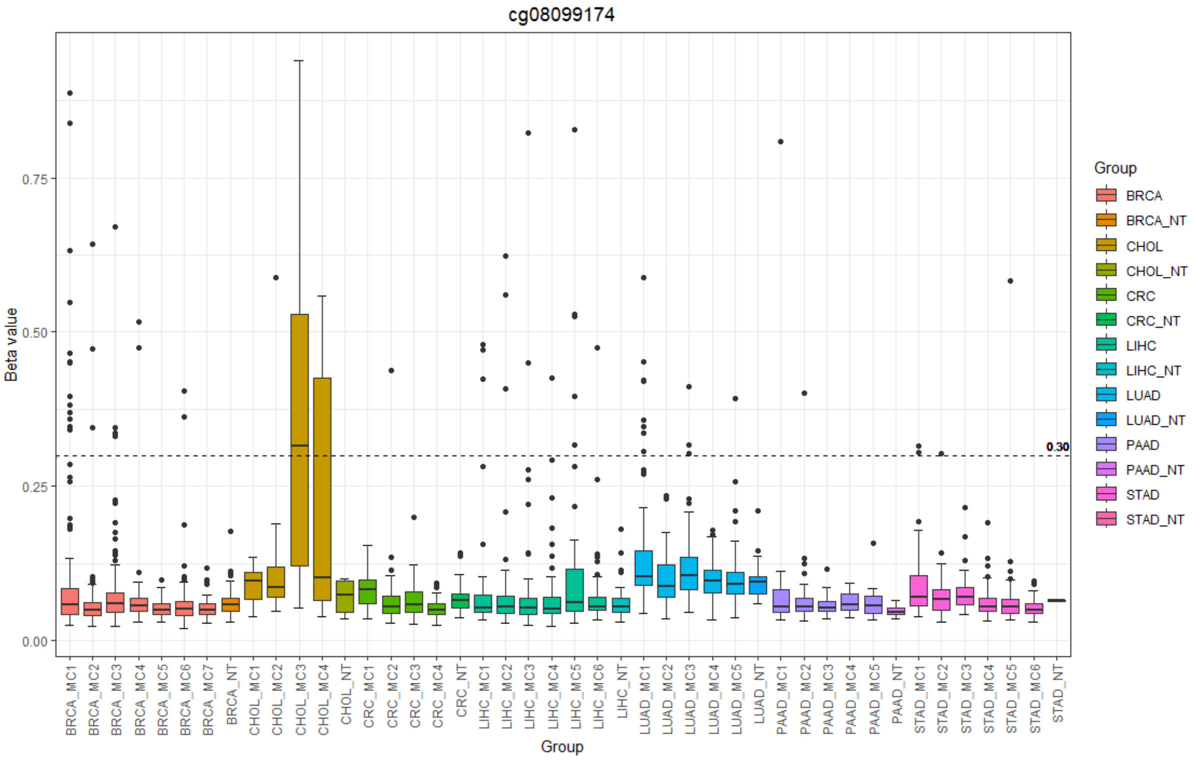

cg08099174
Beta value
Group
0.30
Group
BRCA
BRCA_NT
CHOL
CHOL_NT
CRC
CRC_NT
LIHC
LIHC_NT
LUAD
LUAD_NT
PAAD
PAAD_NT
STAD
STAD_NT

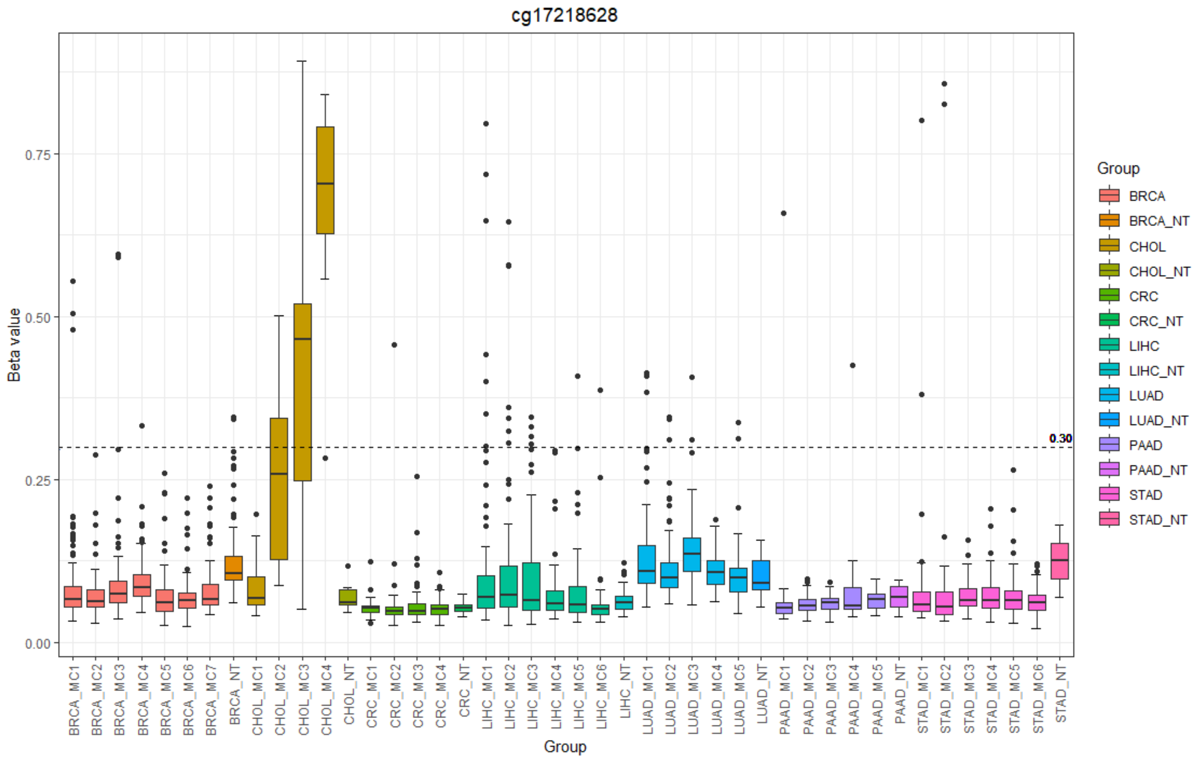
cg17218628
Beta value
0.00
0.25
0.50
0.75
0.30
Group
BRCA_MC1
BRCA_MC2
BRCA_MC3
BRCA_MC4
BRCA_MC5
BRCA_MC6
BRCA_MC7
BRCA_NT
CHOL_MC1
CHOL_MC2
CHOL_MC3
CHOL_MC4
CHOL_NT
CRC_MC1
CRC_MC2
CRC_MC3
CRC_MC4
CRC_NT
LIHC_MC1
LIHC_MC2
LIHC_MC3
LIHC_MC4
LIHC_MC5
LIHC_MC6
LIHC_NT
LUAD_MC1
LUAD_MC2
LUAD_MC3
LUAD_MC4
LUAD_MC5
LUAD_NT
PAAD_MC1
PAAD_MC2
PAAD_MC3
PAAD_MC4
PAAD_MC5
PAAD_NT
STAD_MC1
STAD_MC2
STAD_MC3
STAD_MC4
STAD_MC5
STAD_MC6
STAD_NT
Group
BRCA
BRCA_NT
CHOL
CHOL_NT
CRC
CRC_NT
LIHC
LIHC_NT
LUAD
LUAD_NT
PAAD
PAAD_NT
STAD
STAD_NT

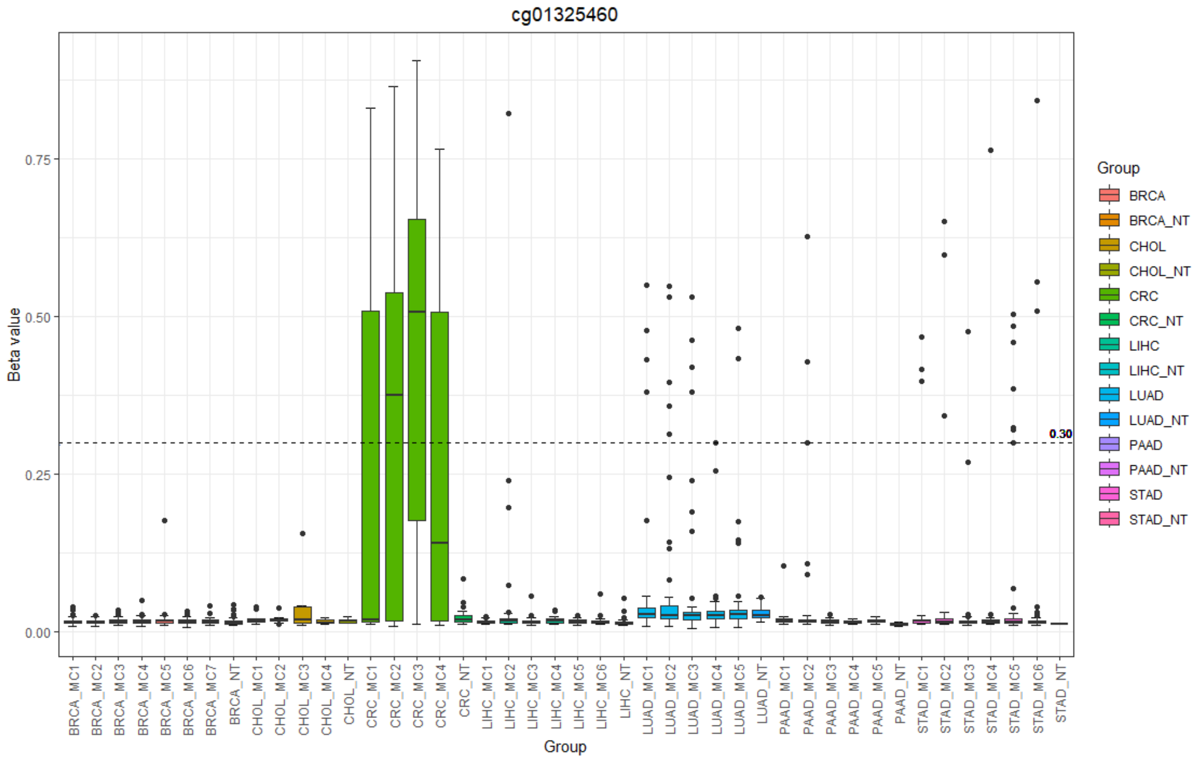
cg01325460
Beta value
0.00
0.25
0.50
0.75
0.30
Group
BRCA_MC1
BRCA_MC2
BRCA_MC3
BRCA_MC4
BRCA_MC5
BRCA_MC6
BRCA_MC7
BRCA_NT
CHOL_MC1
CHOL_MC2
CHOL_MC3
CHOL_MC4
CHOL_NT
CRC_MC1
CRC_MC2
CRC_MC3
CRC_MC4
CRC_NT
LIHC_MC1
LIHC_MC2
LIHC_MC3
LIHC_MC4
LIHC_MC5
LIHC_MC6
LIHC_NT
LUAD_MC1
LUAD_MC2
LUAD_MC3
LUAD_MC4
LUAD_MC5
LUAD_NT
PAAD_MC1
PAAD_MC2
PAAD_MC3
PAAD_MC4
PAAD_MC5
PAAD_NT
STAD_MC1
STAD_MC2
STAD_MC3
STAD_MC4
STAD_MC5
STAD_MC6
STAD_NT
Group
BRCA
BRCA_NT
CHOL
CHOL_NT
CRC
CRC_NT
LIHC
LIHC_NT
LUAD
LUAD_NT
PAAD
PAAD_NT
STAD
STAD_NT

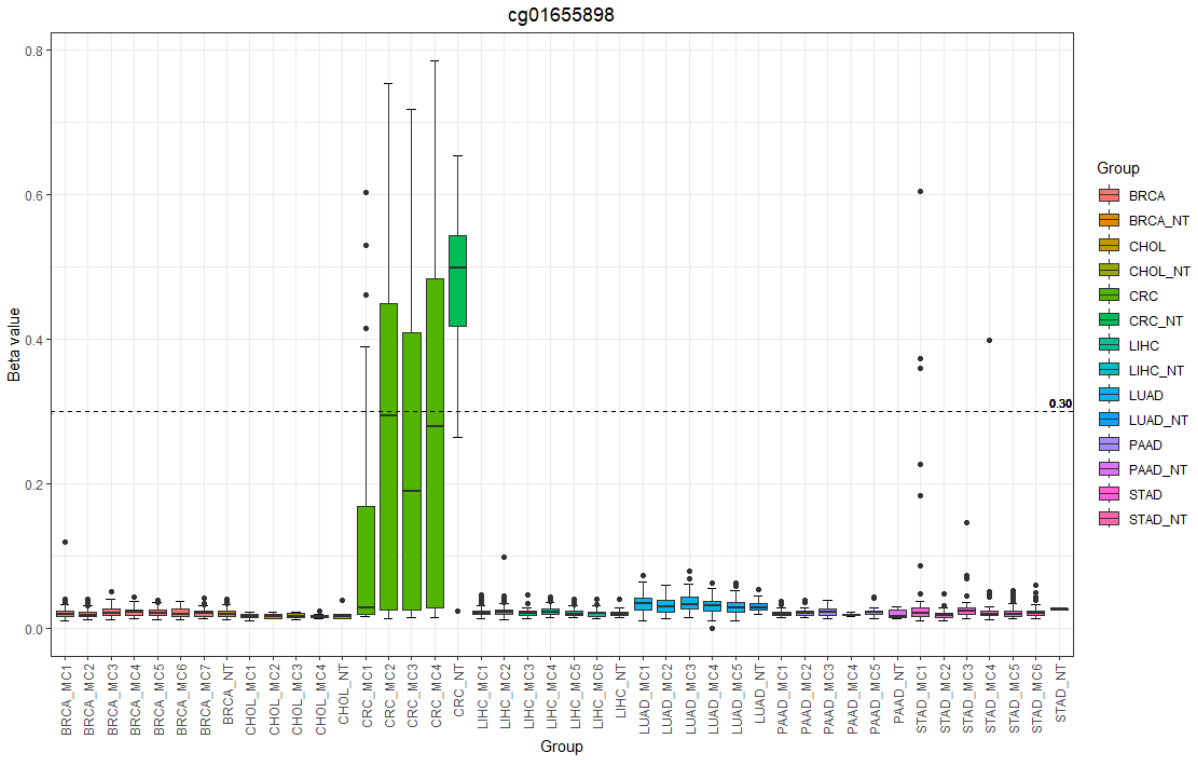

cg01655898
Beta value
Group
0.30
BRCA
BRCA_NT
CHOL
CHOL_NT
CRC
CRC_NT
LIHC
LIHC_NT
LUAD
LUAD_NT
PAAD
PAAD_NT
STAD
STAD_NT

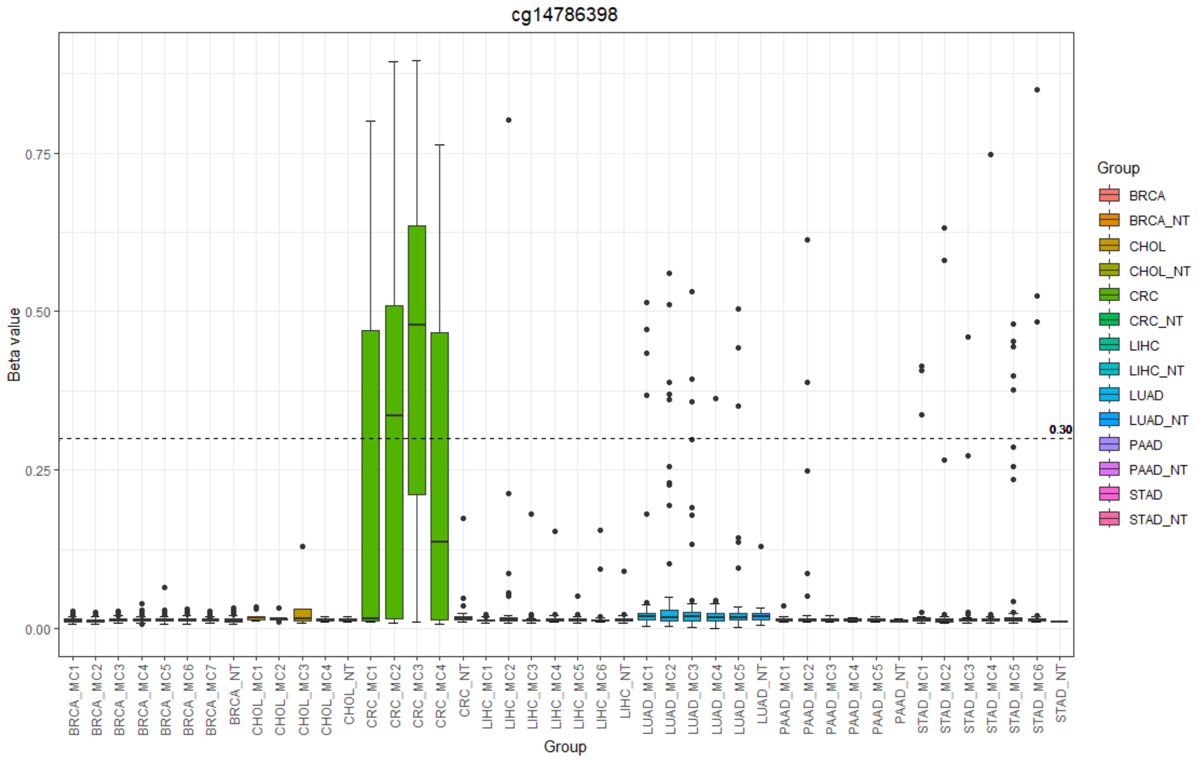

cg14786398
Beta value
Group
0.30
BRCA
BRCA_NT
CHOL
CHOL_NT
CRC
CRC_NT
LIHC
LIHC_NT
LUAD
LUAD_NT
PAAD
PAAD_NT
STAD
STAD_NT

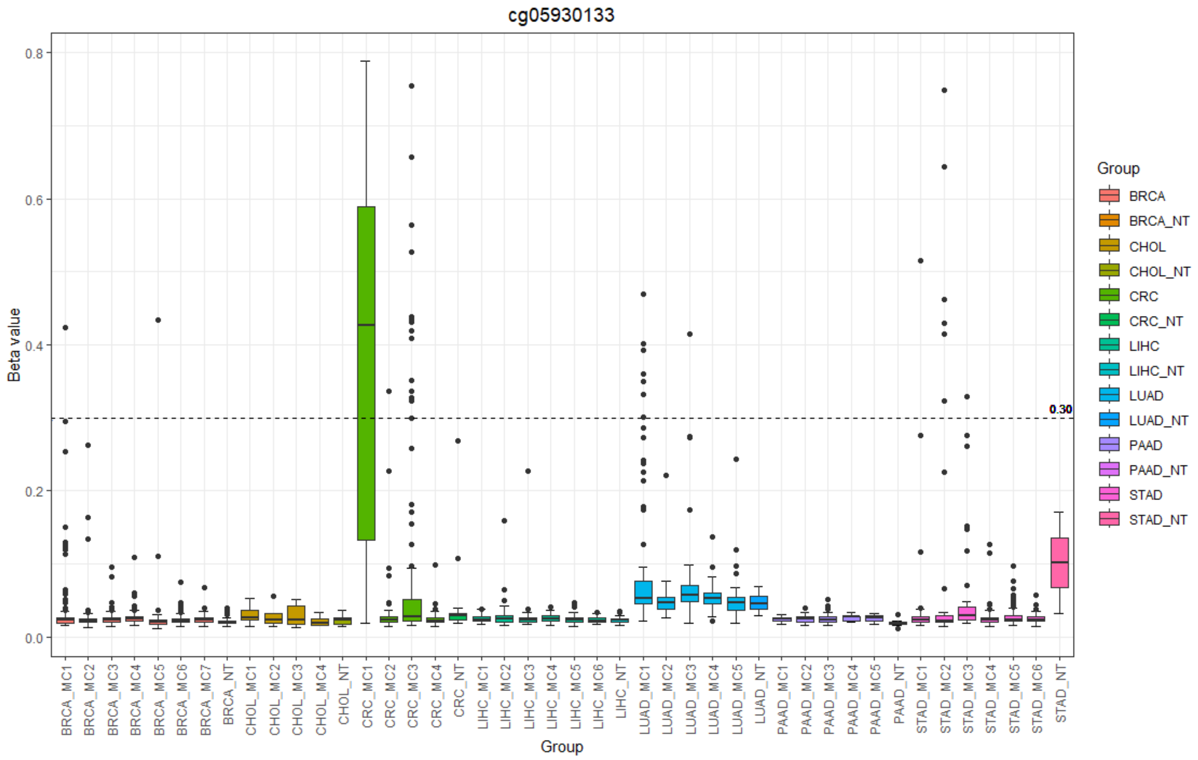

cg05930133
Beta value
Group
0.30
Group
BRCA
BRCA_NT
CHOL
CHOL_NT
CRC
CRC_NT
LIHC
LIHC_NT
LUAD
LUAD_NT
PAAD
PAAD_NT
STAD
STAD_NT

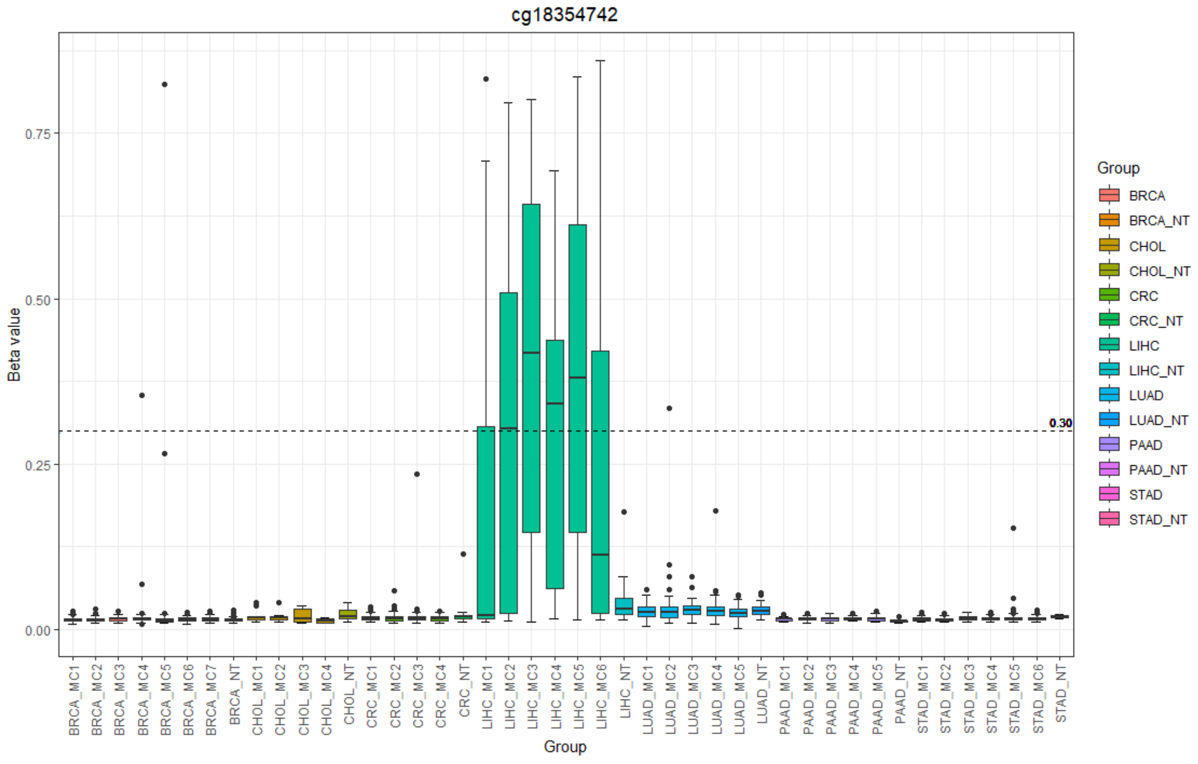

cg18354742
Beta value
Group
0.30
Group
BRCA
BRCA_NT
CHOL
CHOL_NT
CRC
CRC_NT
LIHC
LIHC_NT
LUAD
LUAD_NT
PAAD
PAAD_NT
STAD
STAD_NT

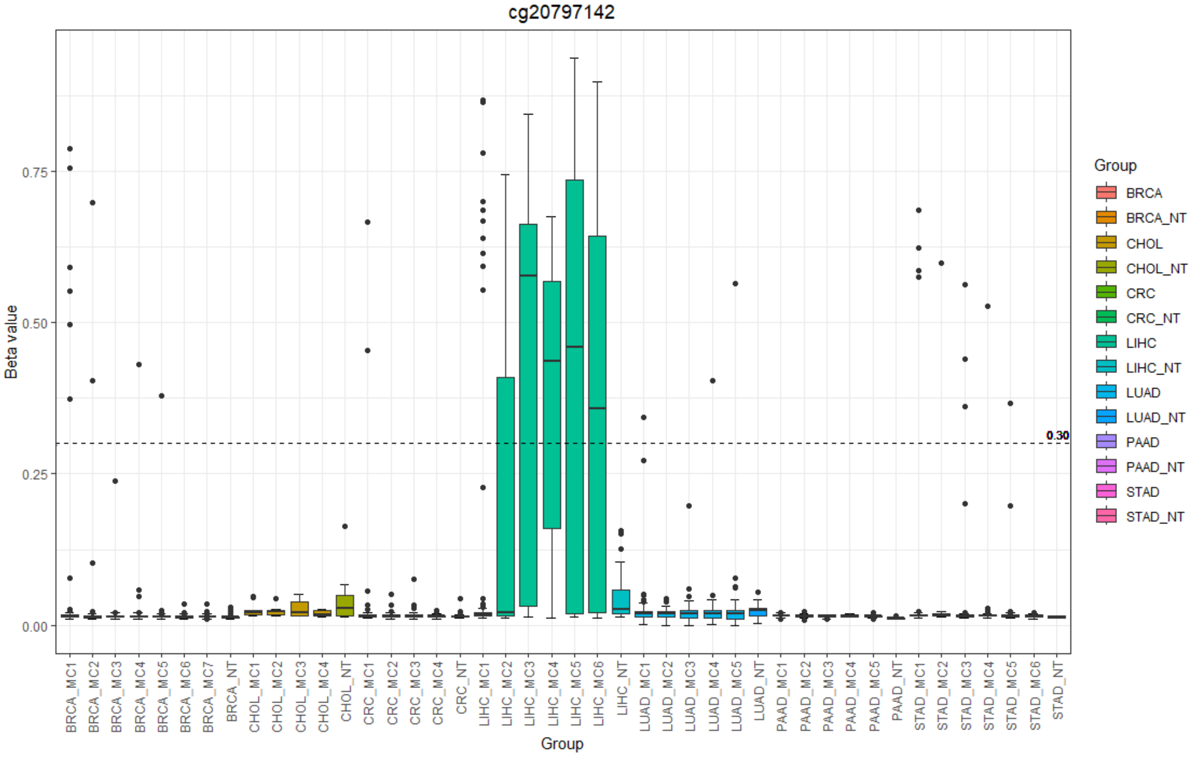

cg20797142
Beta value
0.00
0.25
0.50
0.75
0.30
Group
BRCA_MC1
BRCA_MC2
BRCA_MC3
BRCA_MC4
BRCA_MC5
BRCA_MC6
BRCA_MC7
BRCA_NT
CHOL_MC1
CHOL_MC2
CHOL_MC3
CHOL_MC4
CHOL_NT
CRC_MC1
CRC_MC2
CRC_MC3
CRC_MC4
CRC_NT
LIHC_MC1
LIHC_MC2
LIHC_MC3
LIHC_MC4
LIHC_MC5
LIHC_MC6
LIHC_NT
LUAD_MC1
LUAD_MC2
LUAD_MC3
LUAD_MC4
LUAD_MC5
LUAD_NT
PAAD_MC1
PAAD_MC2
PAAD_MC3
PAAD_MC4
PAAD_MC5
PAAD_NT
STAD_MC1
STAD_MC2
STAD_MC3
STAD_MC4
STAD_MC5
STAD_MC6
STAD_NT
Group
BRCA
BRCA_NT
CHOL
CHOL_NT
CRC
CRC_NT
LIHC
LIHC_NT
LUAD
LUAD_NT
PAAD
PAAD_NT
STAD
STAD_NT

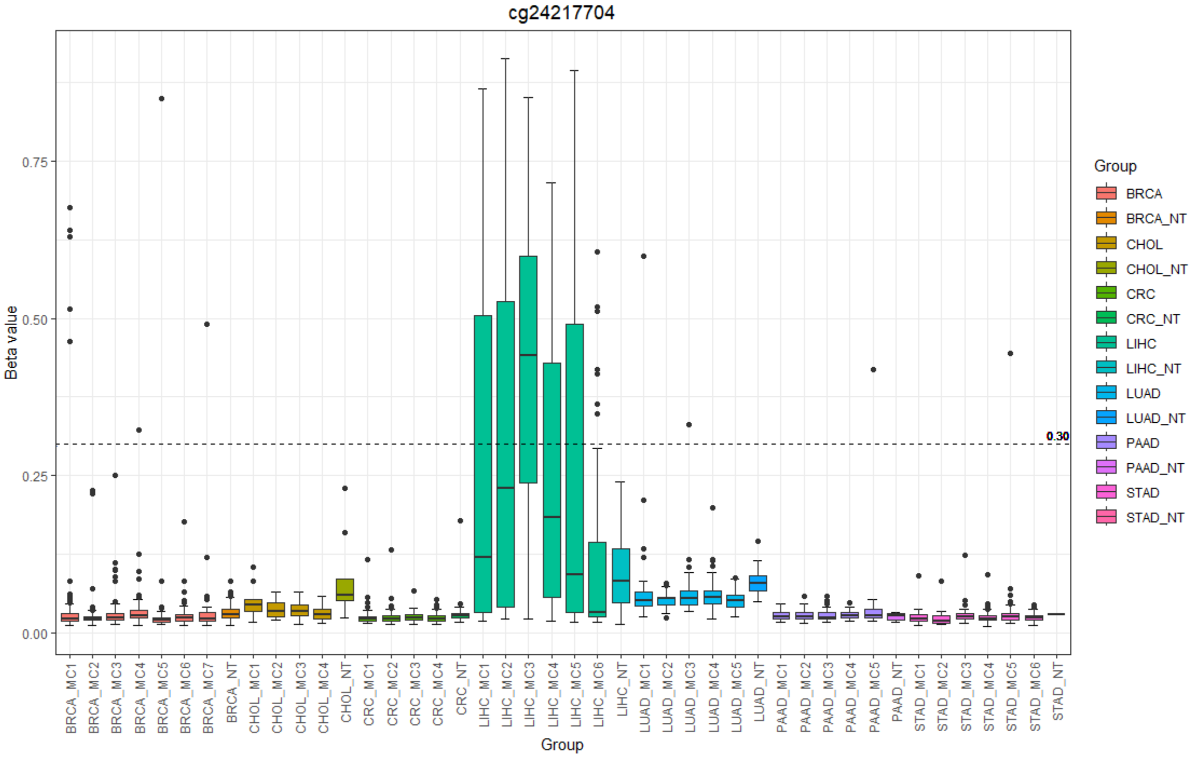

cg24217704
Beta value
0.00
0.25
0.50
0.75
0.30
Group
BRCA_MC1
BRCA_MC2
BRCA_MC3
BRCA_MC4
BRCA_MC5
BRCA_MC6
BRCA_MC7
BRCA_NT
CHOL_MC1
CHOL_MC2
CHOL_MC3
CHOL_MC4
CHOL_NT
CRC_MC1
CRC_MC2
CRC_MC3
CRC_MC4
CRC_NT
LIHC_MC1
LIHC_MC2
LIHC_MC3
LIHC_MC4
LIHC_MC5
LIHC_MC6
LIHC_NT
LUAD_MC1
LUAD_MC2
LUAD_MC3
LUAD_MC4
LUAD_MC5
LUAD_NT
PAAD_MC1
PAAD_MC2
PAAD_MC3
PAAD_MC4
PAAD_MC5
PAAD_NT
STAD_MC1
STAD_MC2
STAD_MC3
STAD_MC4
STAD_MC5
STAD_MC6
STAD_NT
Group
BRCA
BRCA_NT
CHOL
CHOL_NT
CRC
CRC_NT
LIHC
LIHC_NT
LUAD
LUAD_NT
PAAD
PAAD_NT
STAD
STAD_NT

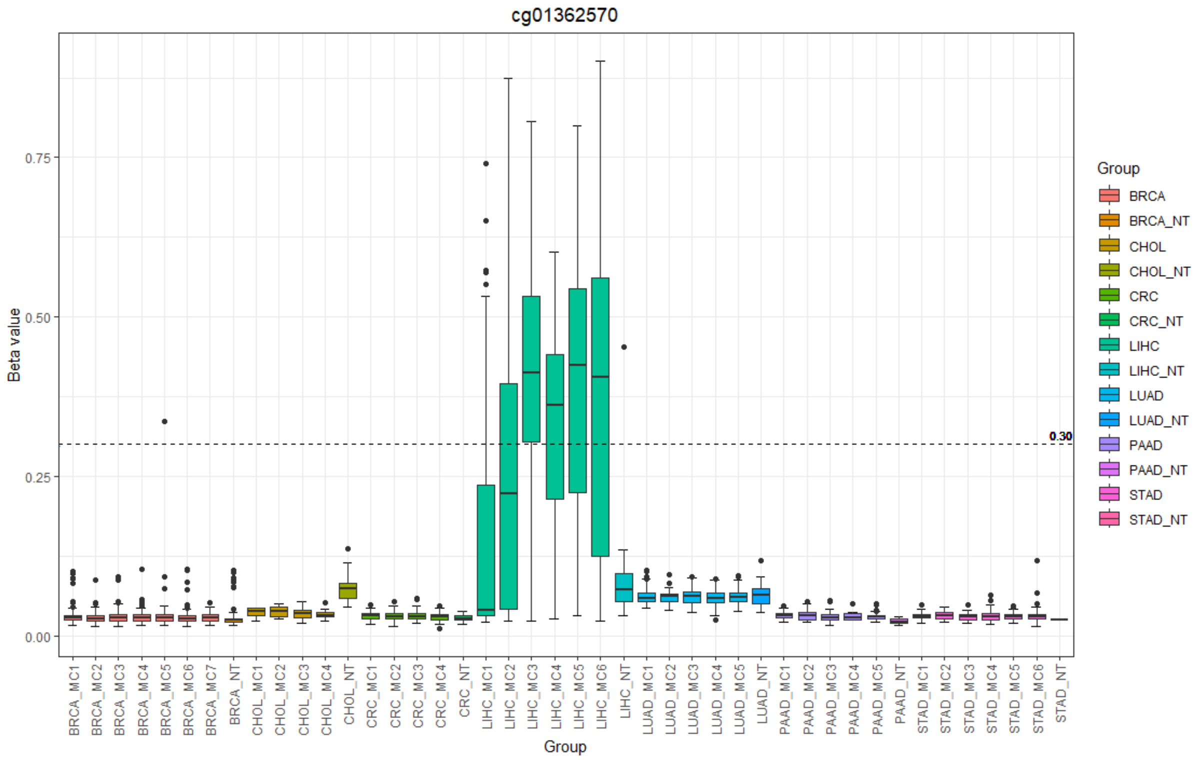
cg01362570
Beta value
0.75
0.50
0.25
0.00
0.30
Group
BRCA_MC1
BRCA_MC2
BRCA_MC3
BRCA_MC4
BRCA_MC5
BRCA_MC6
BRCA_MC7
BRCA_NT
CHOL_MC1
CHOL_MC2
CHOL_MC3
CHOL_MC4
CHOL_NT
CRC_MC1
CRC_MC2
CRC_MC3
CRC_MC4
CRC_NT
LIHC_MC1
LIHC_MC2
LIHC_MC3
LIHC_MC4
LIHC_MC5
LIHC_MC6
LIHC_NT
LUAD_MC1
LUAD_MC2
LUAD_MC3
LUAD_MC4
LUAD_MC5
LUAD_NT
PAAD_MC1
PAAD_MC2
PAAD_MC3
PAAD_MC4
PAAD_MC5
PAAD_NT
STAD_MC1
STAD_MC2
STAD_MC3
STAD_MC4
STAD_MC5
STAD_MC6
STAD_NT
Group
BRCA
BRCA_NT
CHOL
CHOL_NT
CRC
CRC_NT
LIHC
LIHC_NT
LUAD
LUAD_NT
PAAD
PAAD_NT
STAD
STAD_NT

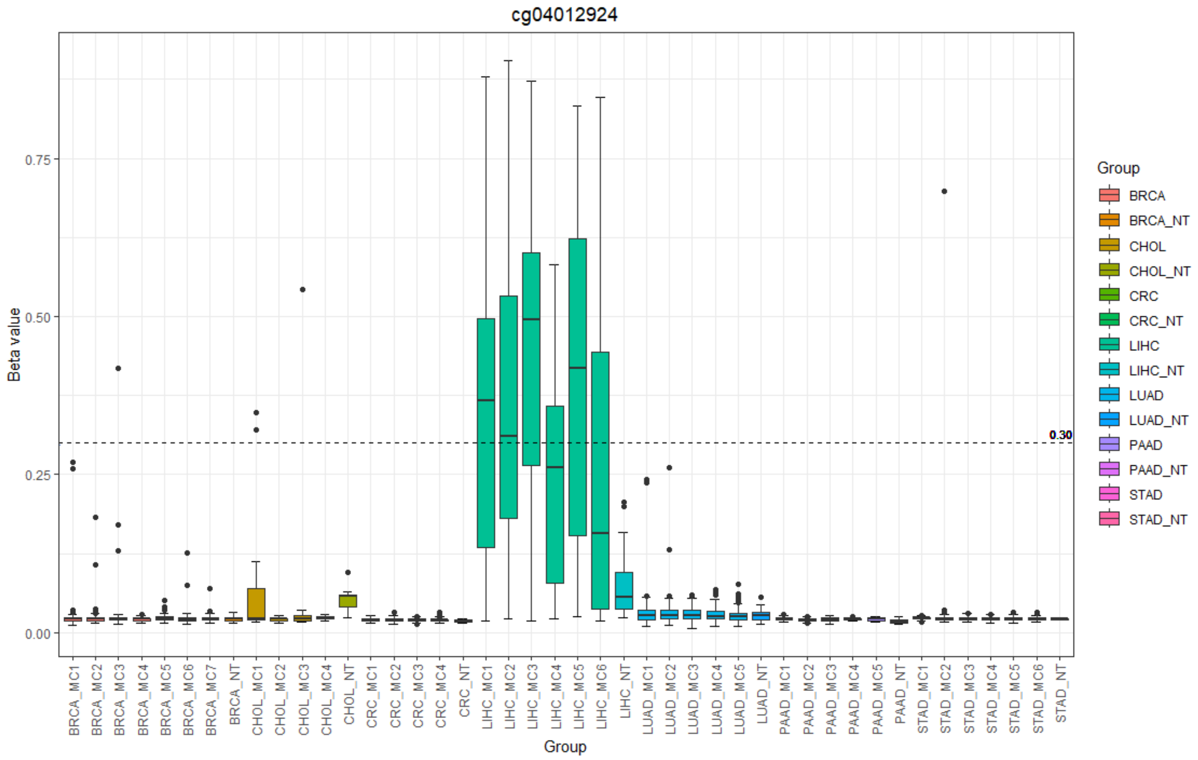
cg04012924
Beta value
0.75
0.50
0.25
0.00
0.30
Group
BRCA_MC1
BRCA_MC2
BRCA_MC3
BRCA_MC4
BRCA_MC5
BRCA_MC6
BRCA_MC7
BRCA_NT
CHOL_MC1
CHOL_MC2
CHOL_MC3
CHOL_MC4
CHOL_NT
CRC_MC1
CRC_MC2
CRC_MC3
CRC_MC4
CRC_NT
LIHC_MC1
LIHC_MC2
LIHC_MC3
LIHC_MC4
LIHC_MC5
LIHC_MC6
LIHC_NT
LUAD_MC1
LUAD_MC2
LUAD_MC3
LUAD_MC4
LUAD_MC5
LUAD_NT
PAAD_MC1
PAAD_MC2
PAAD_MC3
PAAD_MC4
PAAD_MC5
PAAD_NT
STAD_MC1
STAD_MC2
STAD_MC3
STAD_MC4
STAD_MC5
STAD_MC6
STAD_NT
Group
BRCA
BRCA_NT
CHOL
CHOL_NT
CRC
CRC_NT
LIHC
LIHC_NT
LUAD
LUAD_NT
PAAD
PAAD_NT
STAD
STAD_NT

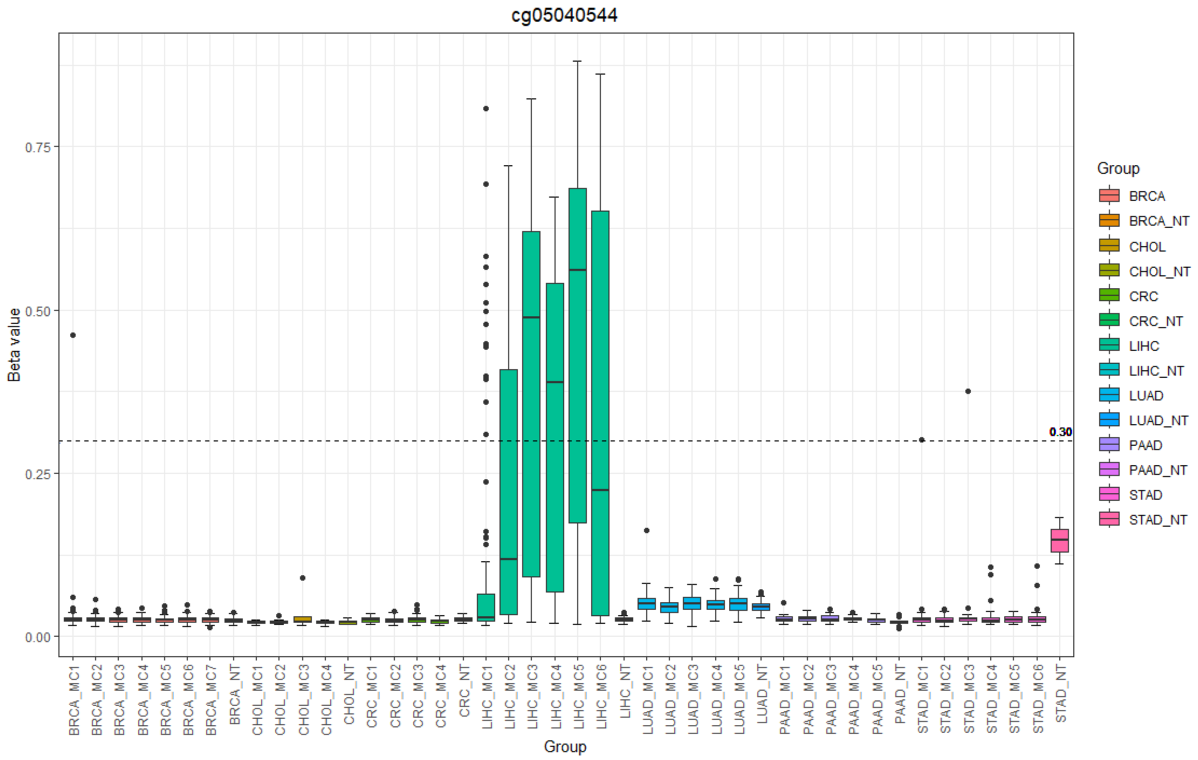
cg05040544
Beta value
0.00
0.25
0.50
0.75
0.30
Group
BRCA_MC1
BRCA_MC2
BRCA_MC3
BRCA_MC4
BRCA_MC5
BRCA_MC6
BRCA_MC7
BRCA_NT
CHOL_MC1
CHOL_MC2
CHOL_MC3
CHOL_MC4
CHOL_NT
CRC_MC1
CRC_MC2
CRC_MC3
CRC_MC4
CRC_NT
LIHC_MC1
LIHC_MC2
LIHC_MC3
LIHC_MC4
LIHC_MC5
LIHC_MC6
LIHC_NT
LUAD_MC1
LUAD_MC2
LUAD_MC3
LUAD_MC4
LUAD_MC5
LUAD_NT
PAAD_MC1
PAAD_MC2
PAAD_MC3
PAAD_MC4
PAAD_MC5
PAAD_NT
STAD_MC1
STAD_MC2
STAD_MC3
STAD_MC4
STAD_MC5
STAD_MC6
STAD_NT
Group
BRCA
BRCA_NT
CHOL
CHOL_NT
CRC
CRC_NT
LIHC
LIHC_NT
LUAD
LUAD_NT
PAAD
PAAD_NT
STAD
STAD_NT

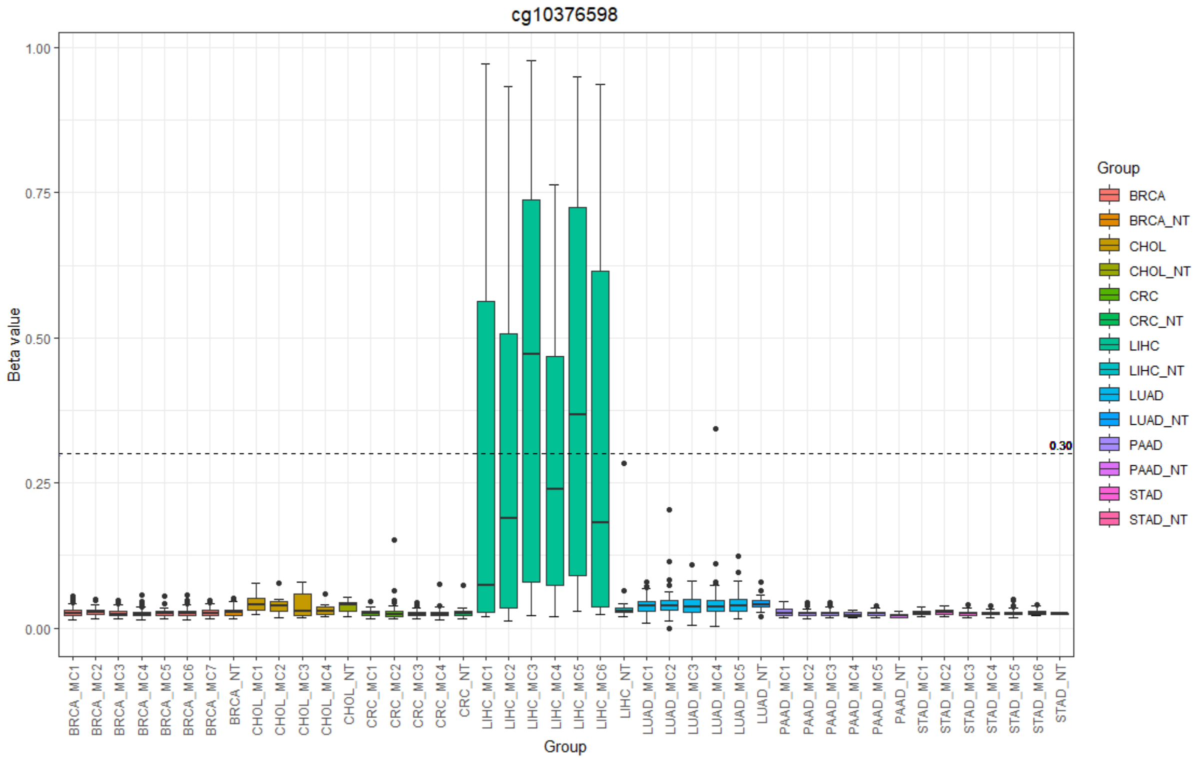
cg10376598
Beta value
0.00
0.25
0.50
0.75
1.00
0.30
Group
BRCA_MC1
BRCA_MC2
BRCA_MC3
BRCA_MC4
BRCA_MC5
BRCA_MC6
BRCA_MC7
BRCA_NT
CHOL_MC1
CHOL_MC2
CHOL_MC3
CHOL_MC4
CHOL_NT
CRC_MC1
CRC_MC2
CRC_MC3
CRC_MC4
CRC_NT
LIHC_MC1
LIHC_MC2
LIHC_MC3
LIHC_MC4
LIHC_MC5
LIHC_MC6
LIHC_NT
LUAD_MC1
LUAD_MC2
LUAD_MC3
LUAD_MC4
LUAD_MC5
LUAD_NT
PAAD_MC1
PAAD_MC2
PAAD_MC3
PAAD_MC4
PAAD_MC5
PAAD_NT
STAD_MC1
STAD_MC2
STAD_MC3
STAD_MC4
STAD_MC5
STAD_MC6
STAD_NT
Group
BRCA
BRCA_NT
CHOL
CHOL_NT
CRC
CRC_NT
LIHC
LIHC_NT
LUAD
LUAD_NT
PAAD
PAAD_NT
STAD
STAD_NT

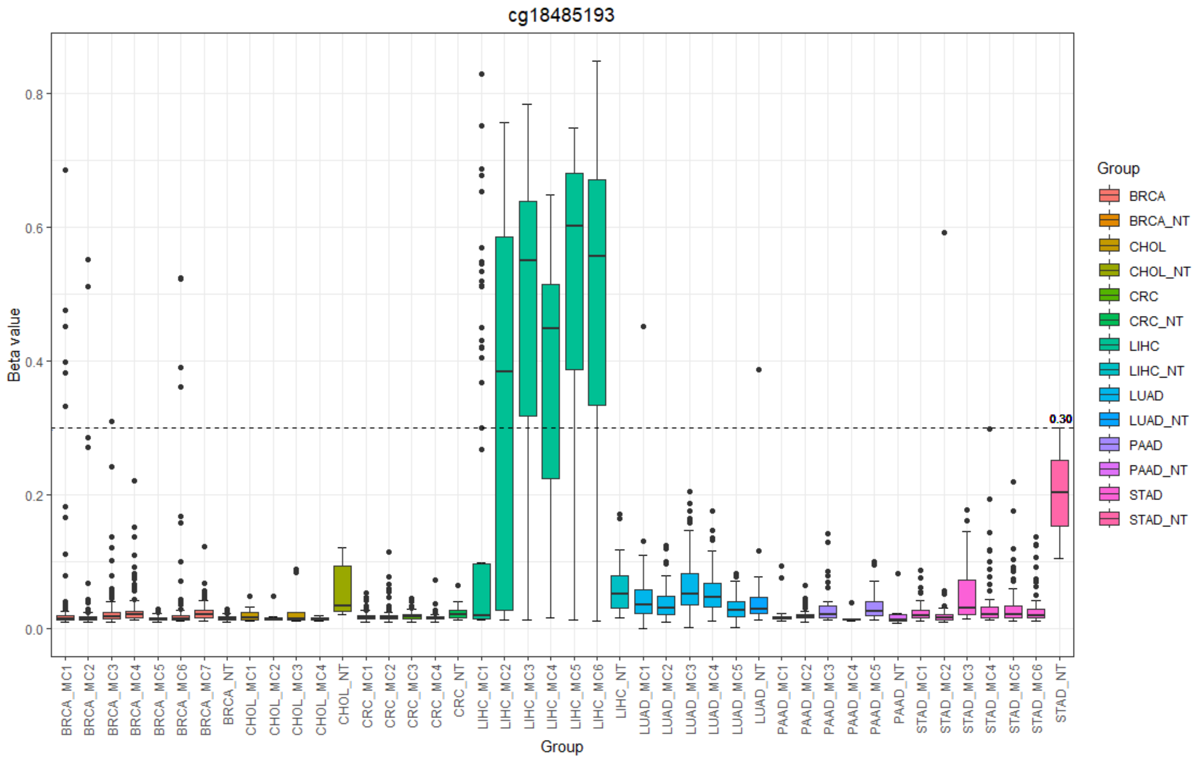
cg18485193
Beta value
0.0
0.2
0.4
0.6
0.8
0.30
Group
BRCA_MC1 BRCA_MC2 BRCA_MC3 BRCA_MC4 BRCA_MC5 BRCA_MC6 BRCA_MC7 BRCA_NT CHOL_MC1 CHOL_MC2 CHOL_MC3 CHOL_MC4 CHOL_NT CRC_MC1 CRC_MC2 CRC_MC3 CRC_MC4 CRC_NT LIHC_MC1 LIHC_MC2 LIHC_MC3 LIHC_MC4 LIHC_MC5 LIHC_MC6 LIHC_NT LUAD_MC1 LUAD_MC2 LUAD_MC3 LUAD_MC4 LUAD_MC5 LUAD_NT PAAD_MC1 PAAD_MC2 PAAD_MC3 PAAD_MC4 PAAD_MC5 PAAD_NT STAD_MC1 STAD_MC2 STAD_MC3 STAD_MC4 STAD_MC5 STAD_MC6 STAD_NT
Group
BRCA
BRCA_NT
CHOL
CHOL_NT
CRC
CRC_NT
LIHC
LIHC_NT
LUAD
LUAD_NT
PAAD
PAAD_NT
STAD
STAD_NT

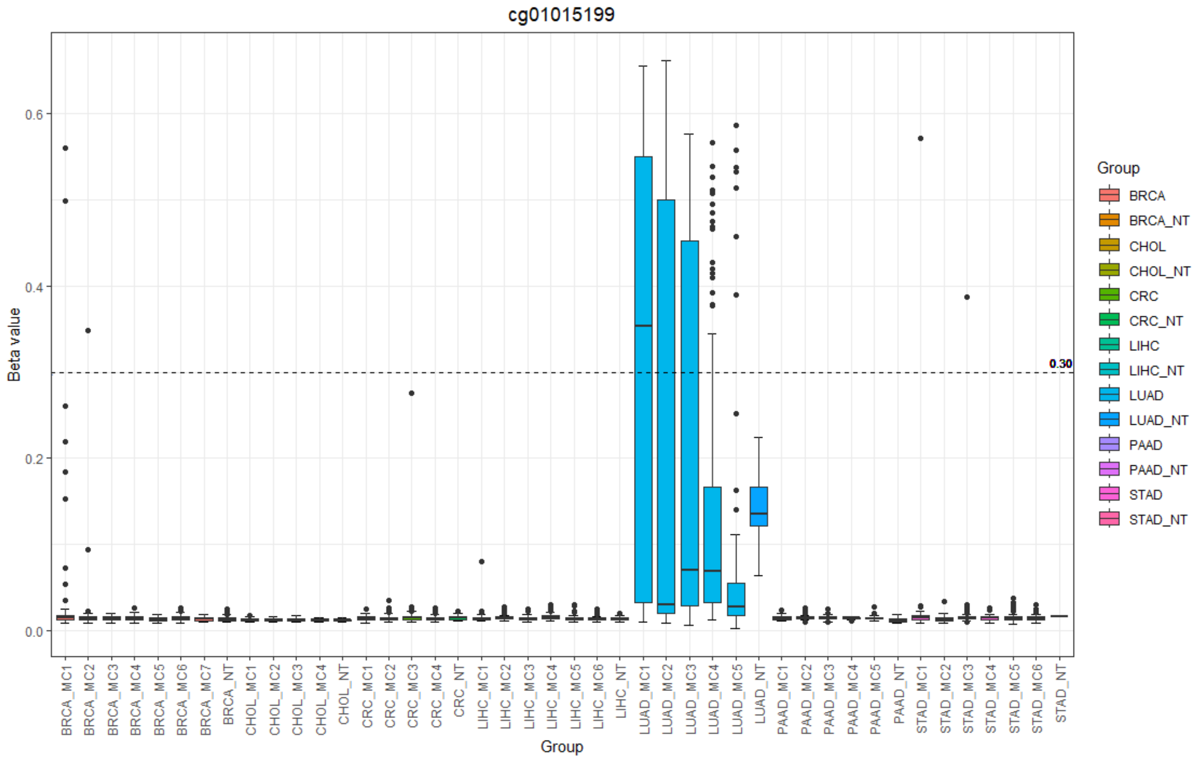
cg01015199
Beta value
0.0
0.2
0.4
0.6
0.30
Group
BRCA_MC1 BRCA_MC2 BRCA_MC3 BRCA_MC4 BRCA_MC5 BRCA_MC6 BRCA_MC7 BRCA_NT CHOL_MC1 CHOL_MC2 CHOL_MC3 CHOL_MC4 CHOL_NT CRC_MC1 CRC_MC2 CRC_MC3 CRC_MC4 CRC_NT LIHC_MC1 LIHC_MC2 LIHC_MC3 LIHC_MC4 LIHC_MC5 LIHC_MC6 LIHC_NT LUAD_MC1 LUAD_MC2 LUAD_MC3 LUAD_MC4 LUAD_MC5 LUAD_NT PAAD_MC1 PAAD_MC2 PAAD_MC3 PAAD_MC4 PAAD_MC5 PAAD_NT STAD_MC1 STAD_MC2 STAD_MC3 STAD_MC4 STAD_MC5 STAD_MC6 STAD_NT
Group
BRCA
BRCA_NT
CHOL
CHOL_NT
CRC
CRC_NT
LIHC
LIHC_NT
LUAD
LUAD_NT
PAAD
PAAD_NT
STAD
STAD_NT

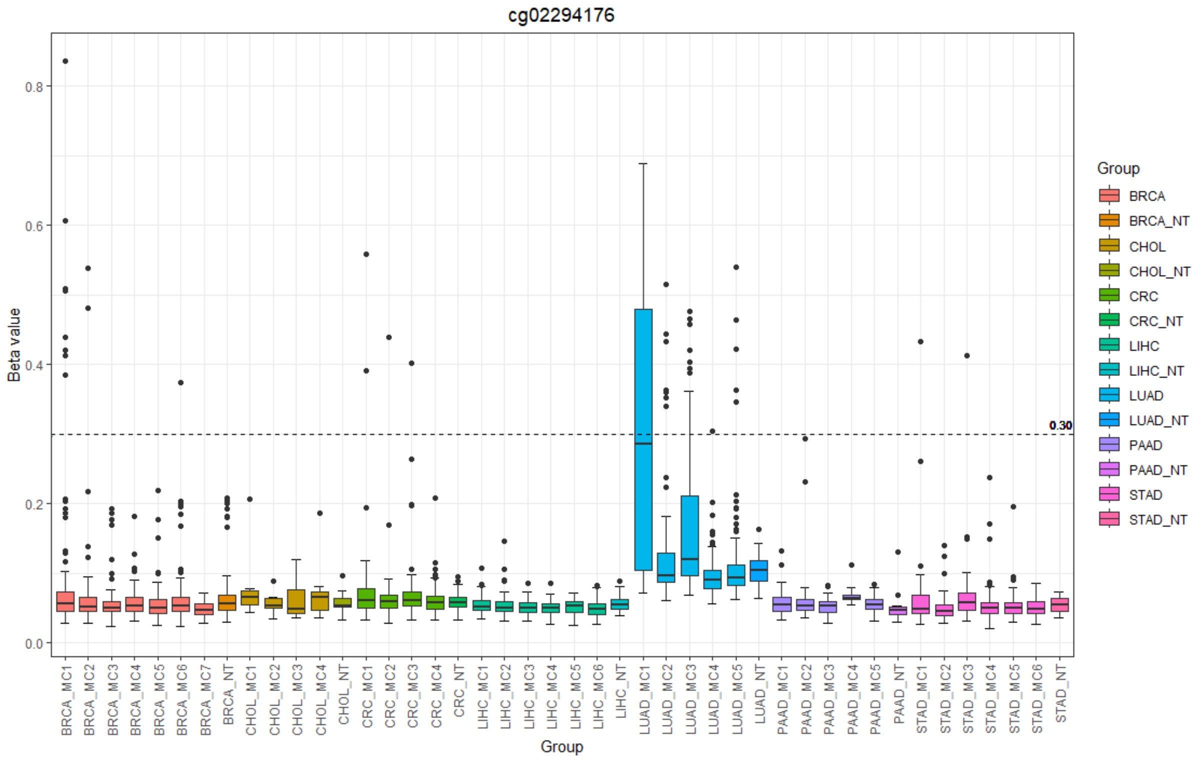
cg02294176
Beta value
Group
0.30
BRCA
BRCA_NT
CHOL
CHOL_NT
CRC
CRC_NT
LIHC
LIHC_NT
LUAD
LUAD_NT
PAAD
PAAD_NT
STAD
STAD_NT

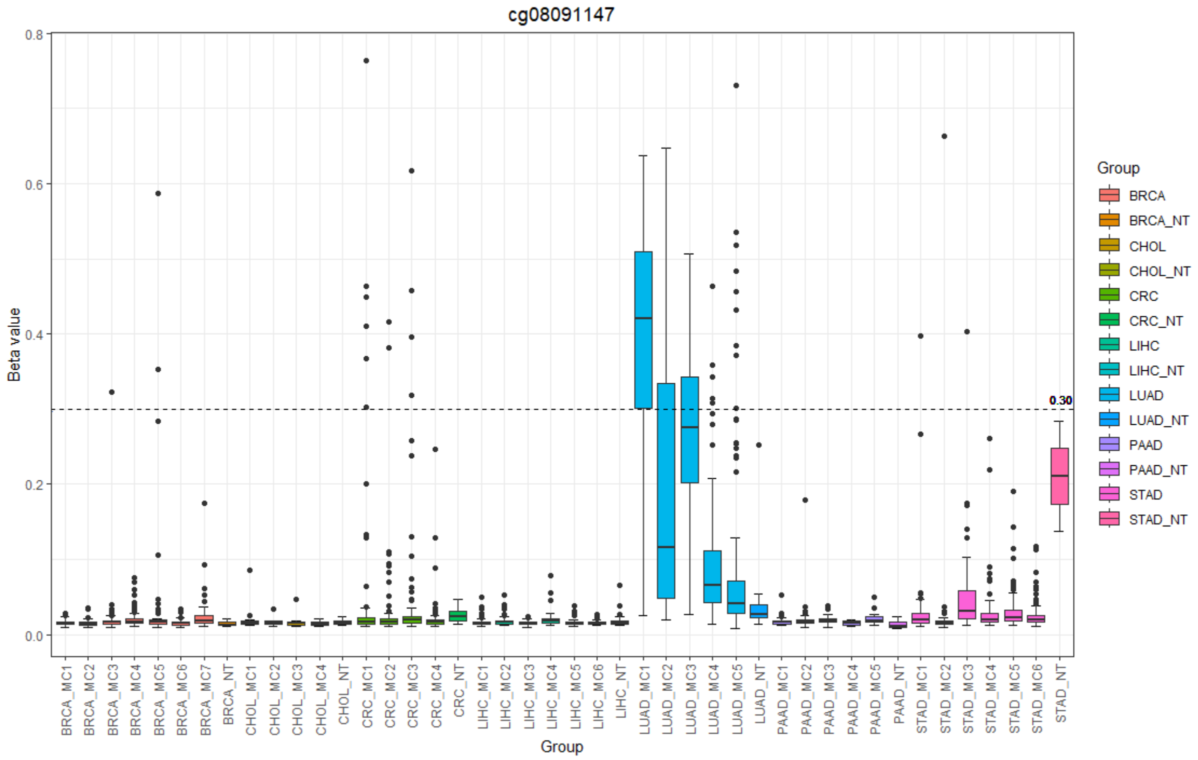
cg08091147
Beta value
Group
0.30
BRCA
BRCA_NT
CHOL
CHOL_NT
CRC
CRC_NT
LIHC
LIHC_NT
LUAD
LUAD_NT
PAAD
PAAD_NT
STAD
STAD_NT

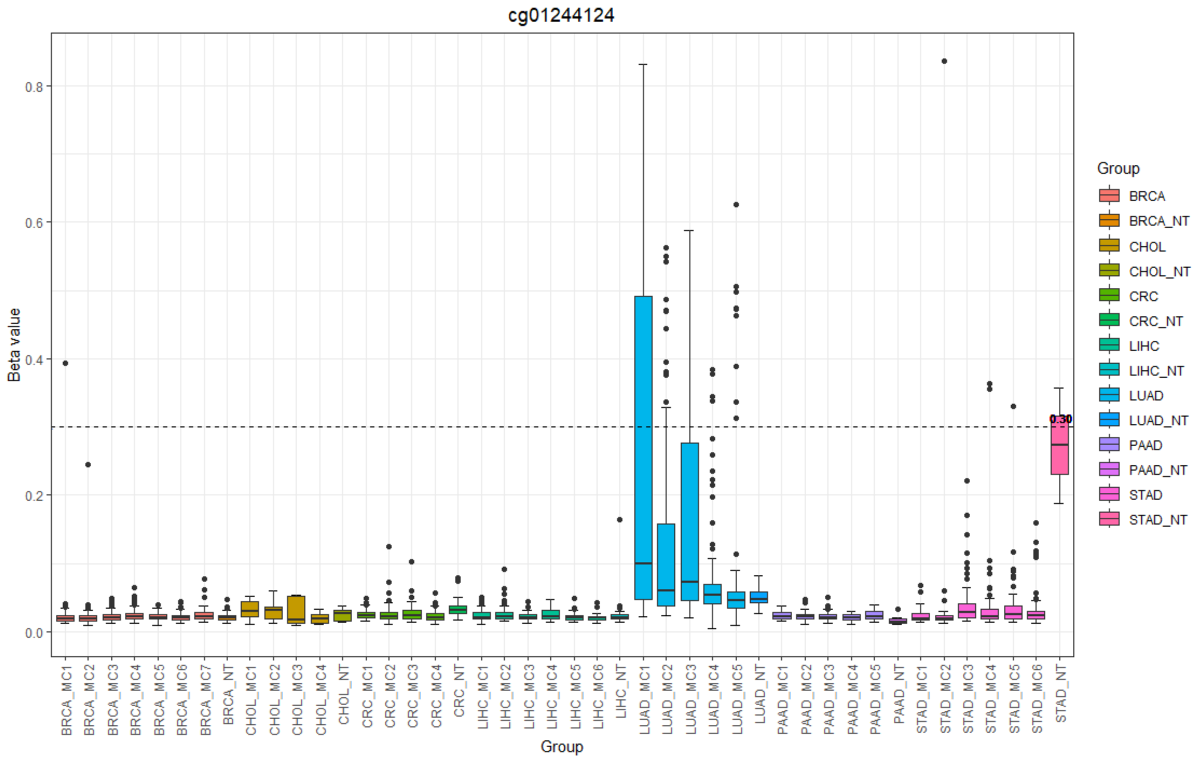

cg01244124
Beta value
0.0
0.2
0.4
0.6
0.8
0.30
Group
BRCA_MC1
BRCA_MC2
BRCA_MC3
BRCA_MC4
BRCA_MC5
BRCA_MC6
BRCA_MC7
BRCA_NT
CHOL_MC1
CHOL_MC2
CHOL_MC3
CHOL_MC4
CHOL_NT
CRC_MC1
CRC_MC2
CRC_MC3
CRC_MC4
CRC_NT
LIHC_MC1
LIHC_MC2
LIHC_MC3
LIHC_MC4
LIHC_MC5
LIHC_MC6
LIHC_NT
LUAD_MC1
LUAD_MC2
LUAD_MC3
LUAD_MC4
LUAD_MC5
LUAD_NT
PAAD_MC1
PAAD_MC2
PAAD_MC3
PAAD_MC4
PAAD_MC5
PAAD_NT
STAD_MC1
STAD_MC2
STAD_MC3
STAD_MC4
STAD_MC5
STAD_MC6
STAD_NT
Group
BRCA
BRCA_NT
CHOL
CHOL_NT
CRC
CRC_NT
LIHC
LIHC_NT
LUAD
LUAD_NT
PAAD
PAAD_NT
STAD
STAD_NT

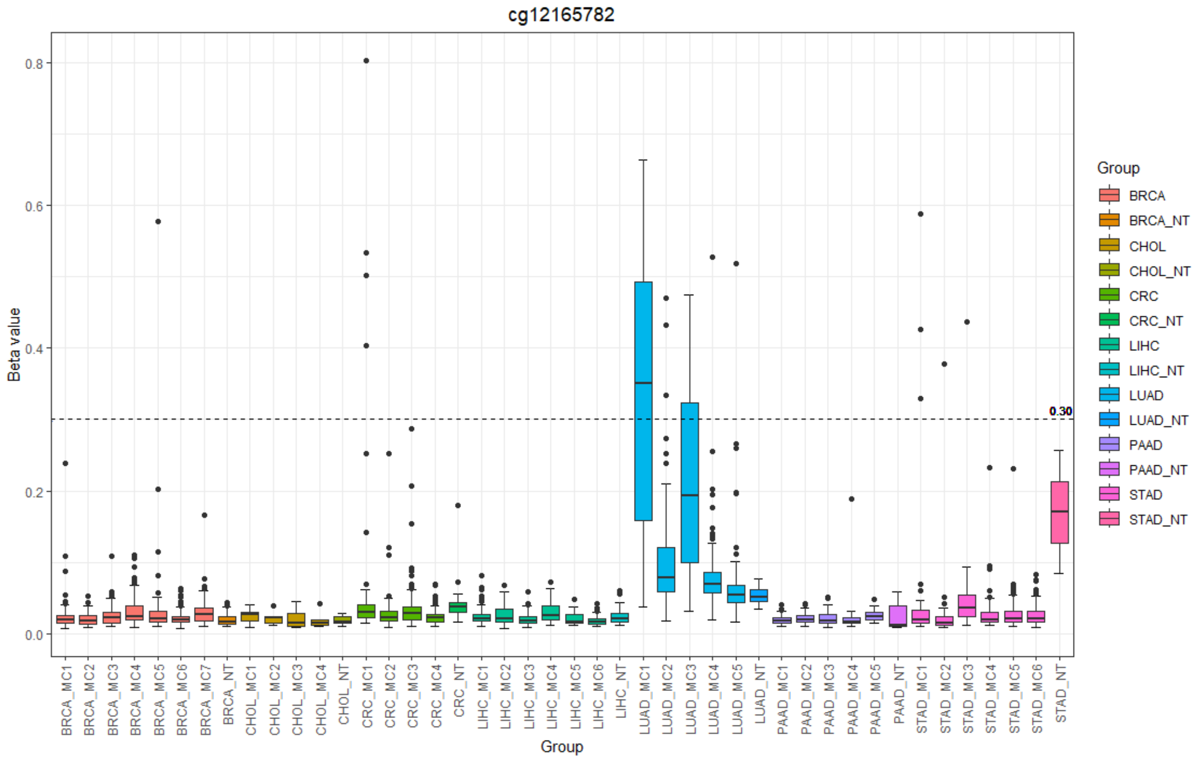

cg12165782
Beta value
0.0
0.2
0.4
0.6
0.8
0.30
Group
BRCA_MC1
BRCA_MC2
BRCA_MC3
BRCA_MC4
BRCA_MC5
BRCA_MC6
BRCA_MC7
BRCA_NT
CHOL_MC1
CHOL_MC2
CHOL_MC3
CHOL_MC4
CHOL_NT
CRC_MC1
CRC_MC2
CRC_MC3
CRC_MC4
CRC_NT
LIHC_MC1
LIHC_MC2
LIHC_MC3
LIHC_MC4
LIHC_MC5
LIHC_MC6
LIHC_NT
LUAD_MC1
LUAD_MC2
LUAD_MC3
LUAD_MC4
LUAD_MC5
LUAD_NT
PAAD_MC1
PAAD_MC2
PAAD_MC3
PAAD_MC4
PAAD_MC5
PAAD_NT
STAD_MC1
STAD_MC2
STAD_MC3
STAD_MC4
STAD_MC5
STAD_MC6
STAD_NT
Group
BRCA
BRCA_NT
CHOL
CHOL_NT
CRC
CRC_NT
LIHC
LIHC_NT
LUAD
LUAD_NT
PAAD
PAAD_NT
STAD
STAD_NT

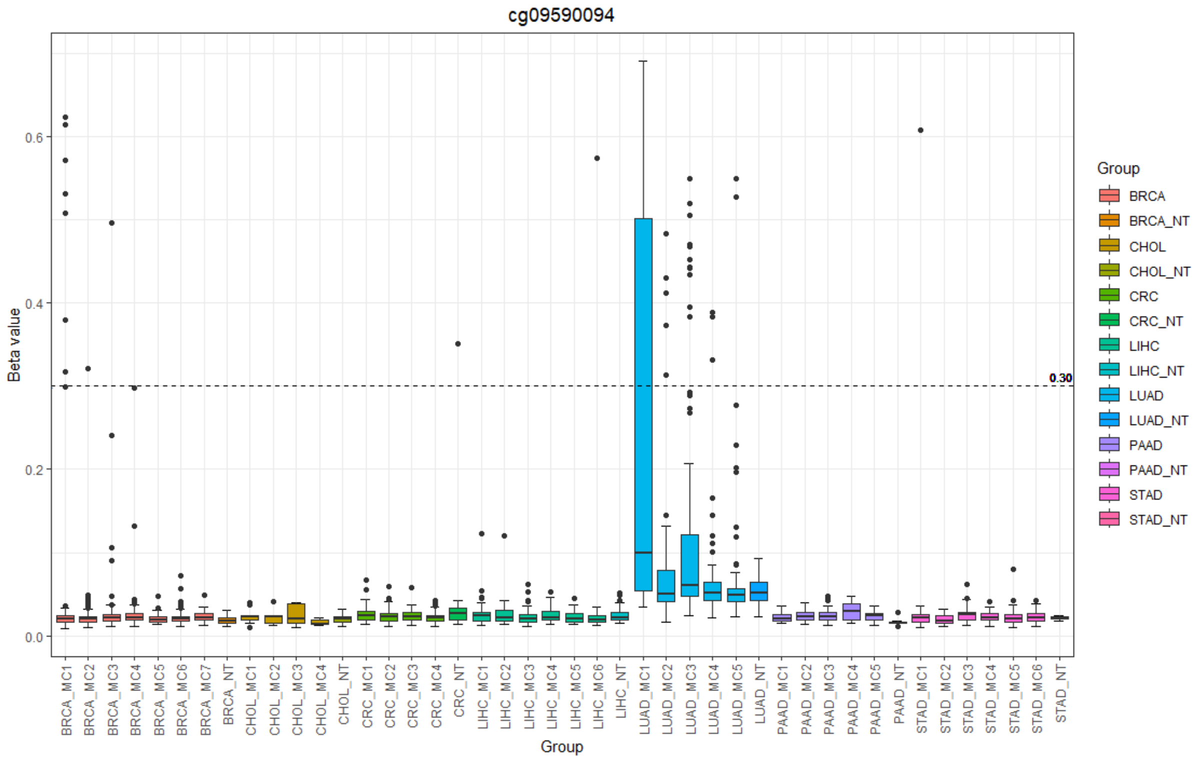

cg09590094
Beta value
0.30
Group
BRCA
BRCA_NT
CHOL
CHOL_NT
CRC
CRC_NT
LIHC
LIHC_NT
LUAD
LUAD_NT
PAAD
PAAD_NT
STAD
STAD_NT

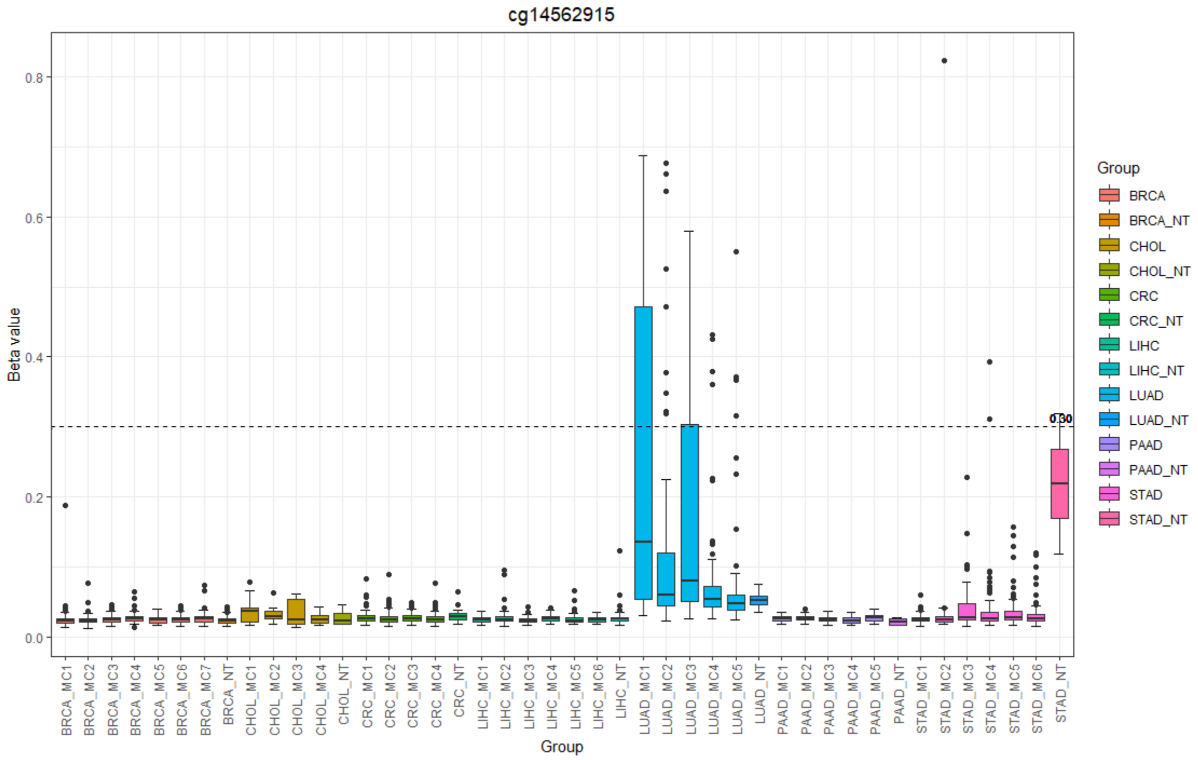

cg14562915
Beta value
0.30
Group
BRCA
BRCA_NT
CHOL
CHOL_NT
CRC
CRC_NT
LIHC
LIHC_NT
LUAD
LUAD_NT
PAAD
PAAD_NT
STAD
STAD_NT

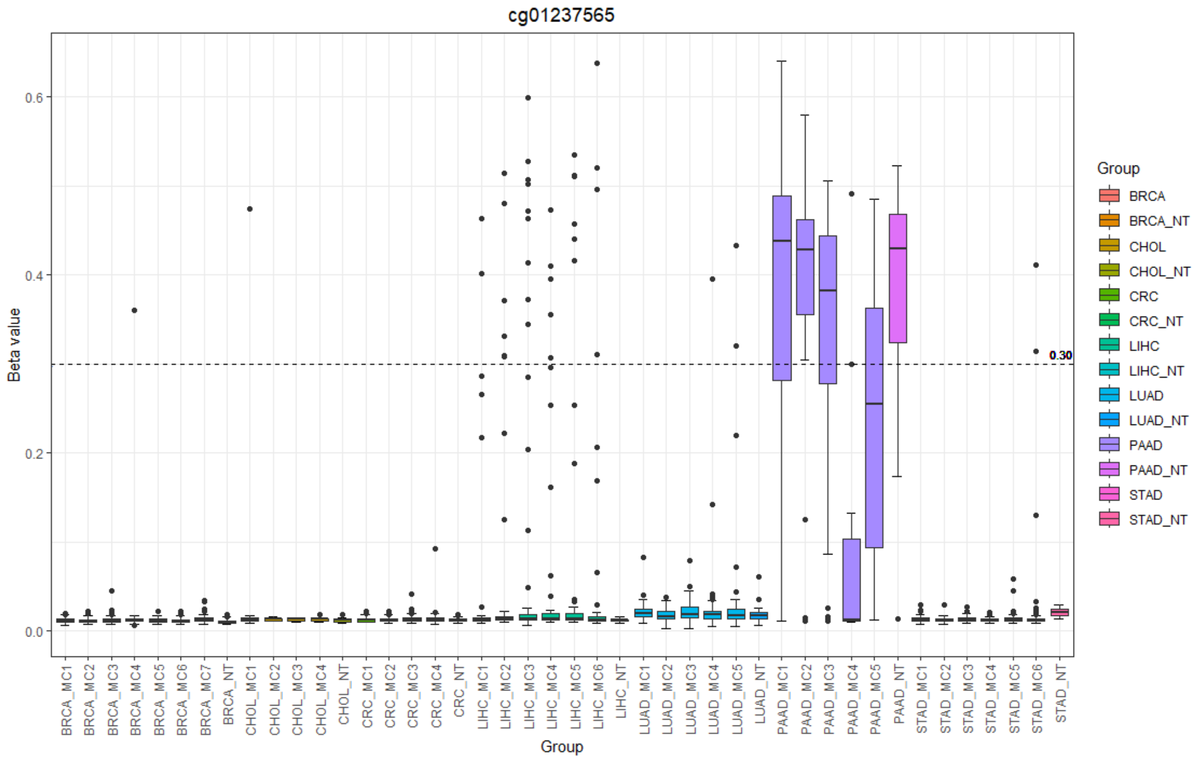
cg01237565
Beta value
Group
0.30
BRCA_MC1
BRCA_MC2
BRCA_MC3
BRCA_MC4
BRCA_MC5
BRCA_MC6
BRCA_MC7
BRCA_NT
CHOL_MC1
CHOL_MC2
CHOL_MC3
CHOL_MC4
CHOL_NT
CRC_MC1
CRC_MC2
CRC_MC3
CRC_MC4
CRC_NT
LIHC_MC1
LIHC_MC2
LIHC_MC3
LIHC_MC4
LIHC_MC5
LIHC_MC6
LIHC_NT
LUAD_MC1
LUAD_MC2
LUAD_MC3
LUAD_MC4
LUAD_MC5
LUAD_NT
PAAD_MC1
PAAD_MC2
PAAD_MC3
PAAD_MC4
PAAD_MC5
PAAD_NT
STAD_MC1
STAD_MC2
STAD_MC3
STAD_MC4
STAD_MC5
STAD_MC6
STAD_NT
Group
BRCA
BRCA_NT
CHOL
CHOL_NT
CRC
CRC_NT
LIHC
LIHC_NT
LUAD
LUAD_NT
PAAD
PAAD_NT
STAD
STAD_NT

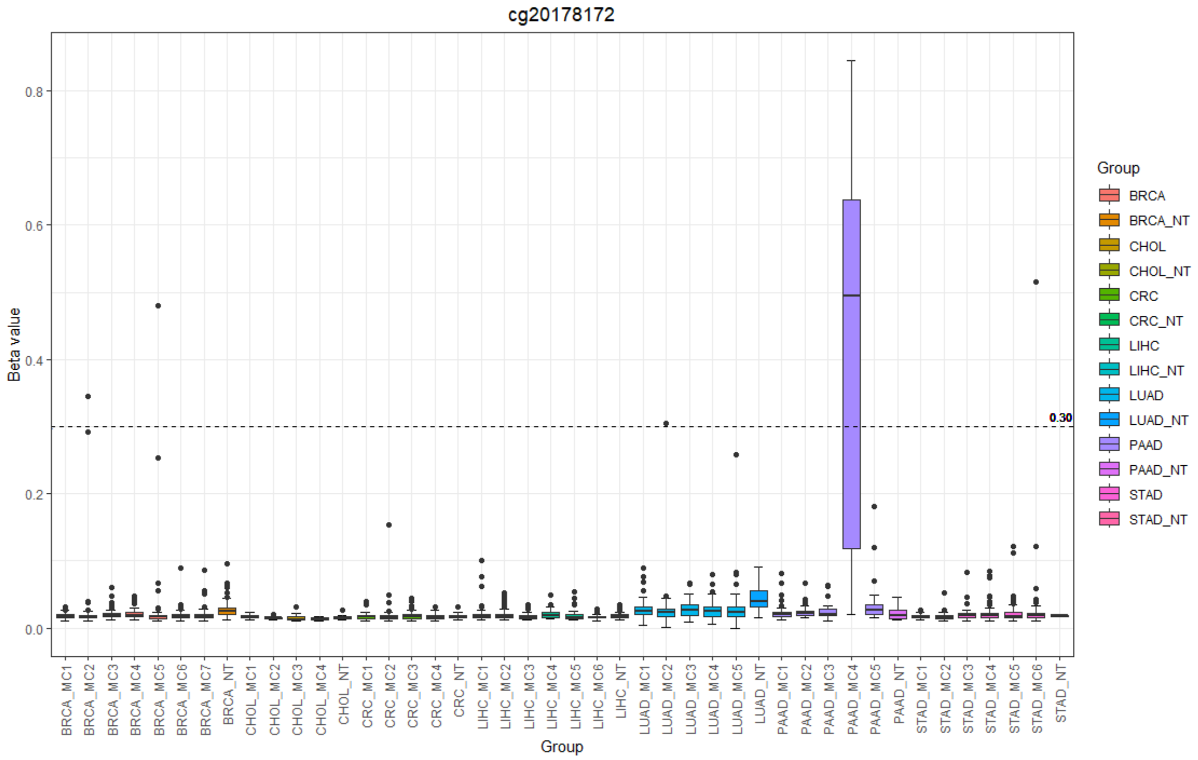
cg20178172
Beta value
Group
0.30
BRCA_MC1
BRCA_MC2
BRCA_MC3
BRCA_MC4
BRCA_MC5
BRCA_MC6
BRCA_MC7
BRCA_NT
CHOL_MC1
CHOL_MC2
CHOL_MC3
CHOL_MC4
CHOL_NT
CRC_MC1
CRC_MC2
CRC_MC3
CRC_MC4
CRC_NT
LIHC_MC1
LIHC_MC2
LIHC_MC3
LIHC_MC4
LIHC_MC5
LIHC_MC6
LIHC_NT
LUAD_MC1
LUAD_MC2
LUAD_MC3
LUAD_MC4
LUAD_MC5
LUAD_NT
PAAD_MC1
PAAD_MC2
PAAD_MC3
PAAD_MC4
PAAD_MC5
PAAD_NT
STAD_MC1
STAD_MC2
STAD_MC3
STAD_MC4
STAD_MC5
STAD_MC6
STAD_NT
Group
BRCA
BRCA_NT
CHOL
CHOL_NT
CRC
CRC_NT
LIHC
LIHC_NT
LUAD
LUAD_NT
PAAD
PAAD_NT
STAD
STAD_NT

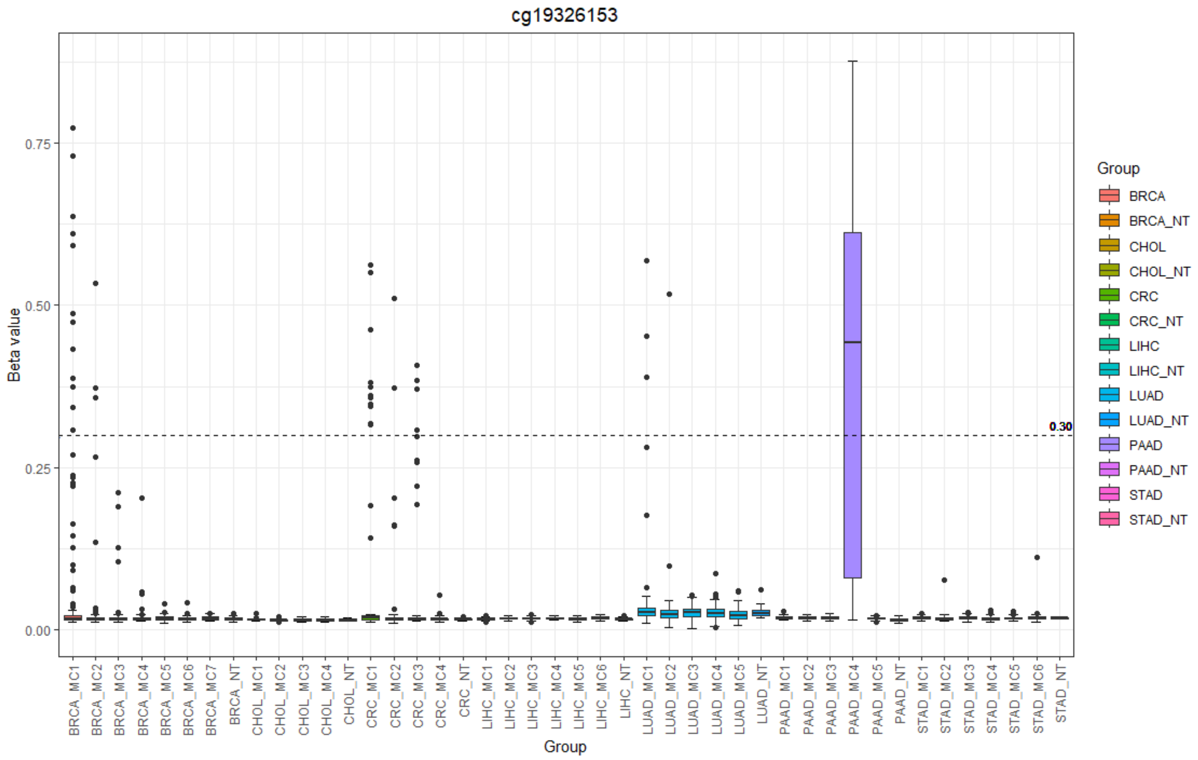
cg19326153
Beta value
0.00
0.25
0.50
0.75
0.30
Group
BRCA_MC1
BRCA_MC2
BRCA_MC3
BRCA_MC4
BRCA_MC5
BRCA_MC6
BRCA_MC7
BRCA_NT
CHOL_MC1
CHOL_MC2
CHOL_MC3
CHOL_MC4
CHOL_NT
CRC_MC1
CRC_MC2
CRC_MC3
CRC_MC4
CRC_NT
LIHC_MC1
LIHC_MC2
LIHC_MC3
LIHC_MC4
LIHC_MC5
LIHC_MC6
LIHC_NT
LUAD_MC1
LUAD_MC2
LUAD_MC3
LUAD_MC4
LUAD_MC5
LUAD_NT
PAAD_MC1
PAAD_MC2
PAAD_MC3
PAAD_MC4
PAAD_MC5
PAAD_NT
STAD_MC1
STAD_MC2
STAD_MC3
STAD_MC4
STAD_MC5
STAD_MC6
STAD_NT
Group
BRCA
BRCA_NT
CHOL
CHOL_NT
CRC
CRC_NT
LIHC
LIHC_NT
LUAD
LUAD_NT
PAAD
PAAD_NT
STAD
STAD_NT

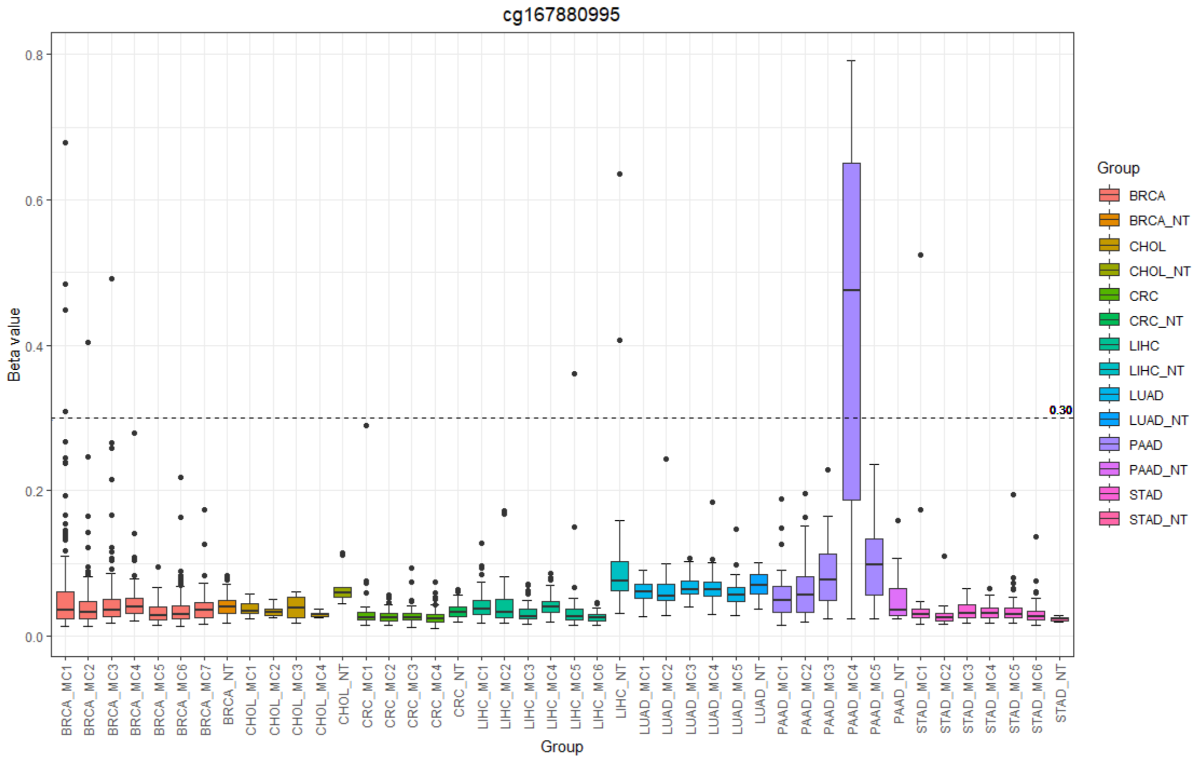
cg167880995
Beta value
0.0
0.2
0.4
0.6
0.8
0.30
Group
BRCA_MC1
BRCA_MC2
BRCA_MC3
BRCA_MC4
BRCA_MC5
BRCA_MC6
BRCA_MC7
BRCA_NT
CHOL_MC1
CHOL_MC2
CHOL_MC3
CHOL_MC4
CHOL_NT
CRC_MC1
CRC_MC2
CRC_MC3
CRC_MC4
CRC_NT
LIHC_MC1
LIHC_MC2
LIHC_MC3
LIHC_MC4
LIHC_MC5
LIHC_MC6
LIHC_NT
LUAD_MC1
LUAD_MC2
LUAD_MC3
LUAD_MC4
LUAD_MC5
LUAD_NT
PAAD_MC1
PAAD_MC2
PAAD_MC3
PAAD_MC4
PAAD_MC5
PAAD_NT
STAD_MC1
STAD_MC2
STAD_MC3
STAD_MC4
STAD_MC5
STAD_MC6
STAD_NT
Group
BRCA
BRCA_NT
CHOL
CHOL_NT
CRC
CRC_NT
LIHC
LIHC_NT
LUAD
LUAD_NT
PAAD
PAAD_NT
STAD
STAD_NT

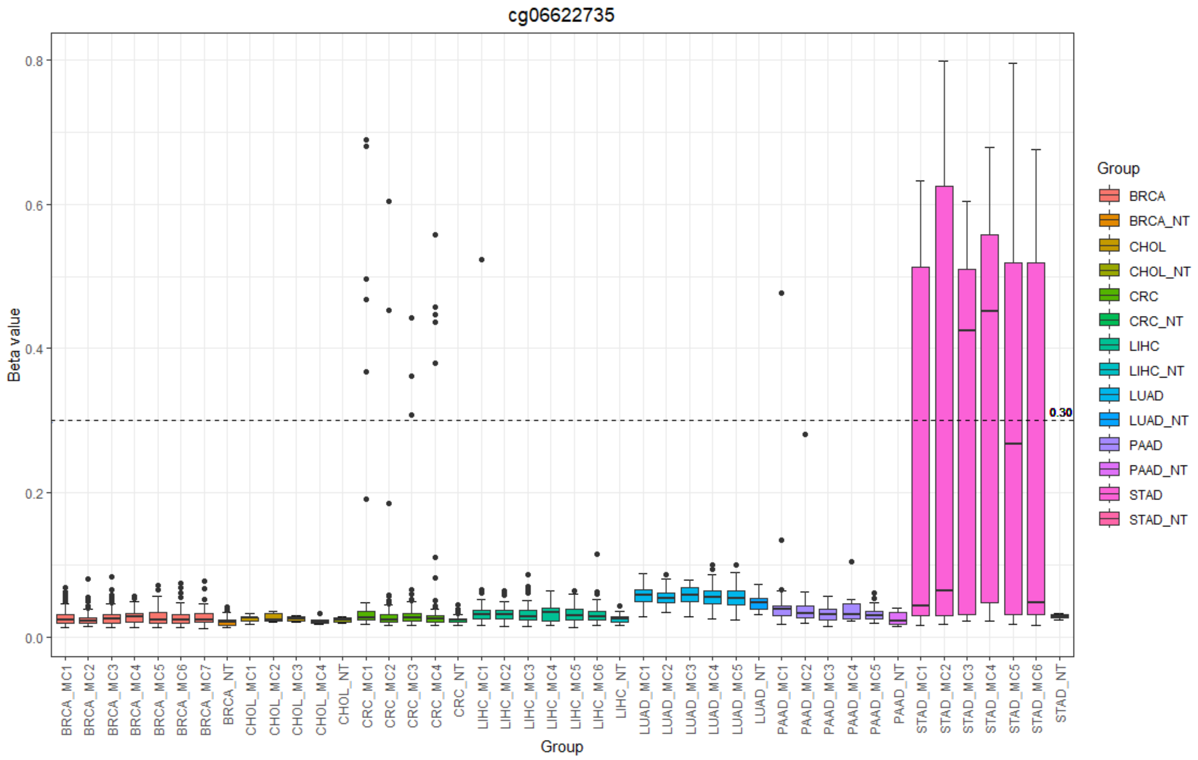

cg06622735
Beta value
0.0
0.2
0.4
0.6
0.8
0.30
Group
BRCA
BRCA_NT
CHOL
CHOL_NT
CRC
CRC_NT
LIHC
LIHC_NT
LUAD
LUAD_NT
PAAD
PAAD_NT
STAD
STAD_NT
BRCA_MC1 BRCA_MC2 BRCA_MC3 BRCA_MC4 BRCA_MC5 BRCA_MC6 BRCA_MC7 BRCA_NT CHOL_MC1 CHOL_MC2 CHOL_MC3 CHOL_MC4 CHOL_NT CRC_MC1 CRC_MC2 CRC_MC3 CRC_MC4 CRC_NT LIHC_MC1 LIHC_MC2 LIHC_MC3 LIHC_MC4 LIHC_MC5 LIHC_MC6 LIHC_NT LUAD_MC1 LUAD_MC2 LUAD_MC3 LUAD_MC4 LUAD_MC5 LUAD_NT PAAD_MC1 PAAD_MC2 PAAD_MC3 PAAD_MC4 PAAD_MC5 PAAD_NT STAD_MC1 STAD_MC2 STAD_MC3 STAD_MC4 STAD_MC5 STAD_MC6 STAD_NT

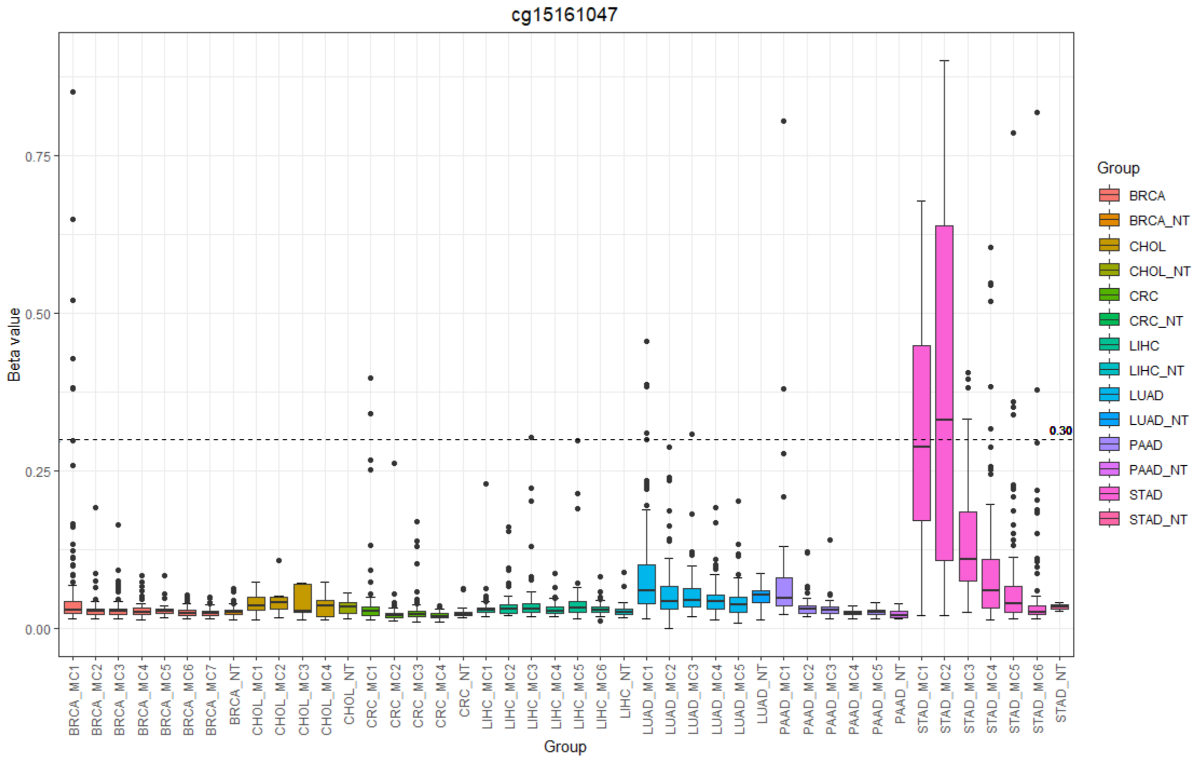

cg15161047
Beta value
0.00
0.25
0.50
0.75
0.30
Group
BRCA
BRCA_NT
CHOL
CHOL_NT
CRC
CRC_NT
LIHC
LIHC_NT
LUAD
LUAD_NT
PAAD
PAAD_NT
STAD
STAD_NT
BRCA_MC1 BRCA_MC2 BRCA_MC3 BRCA_MC4 BRCA_MC5 BRCA_MC6 BRCA_MC7 BRCA_NT CHOL_MC1 CHOL_MC2 CHOL_MC3 CHOL_MC4 CHOL_NT CRC_MC1 CRC_MC2 CRC_MC3 CRC_MC4 CRC_NT LIHC_MC1 LIHC_MC2 LIHC_MC3 LIHC_MC4 LIHC_MC5 LIHC_MC6 LIHC_NT LUAD_MC1 LUAD_MC2 LUAD_MC3 LUAD_MC4 LUAD_MC5 LUAD_NT PAAD_MC1 PAAD_MC2 PAAD_MC3 PAAD_MC4 PAAD_MC5 PAAD_NT STAD_MC1 STAD_MC2 STAD_MC3 STAD_MC4 STAD_MC5 STAD_MC6 STAD_NT

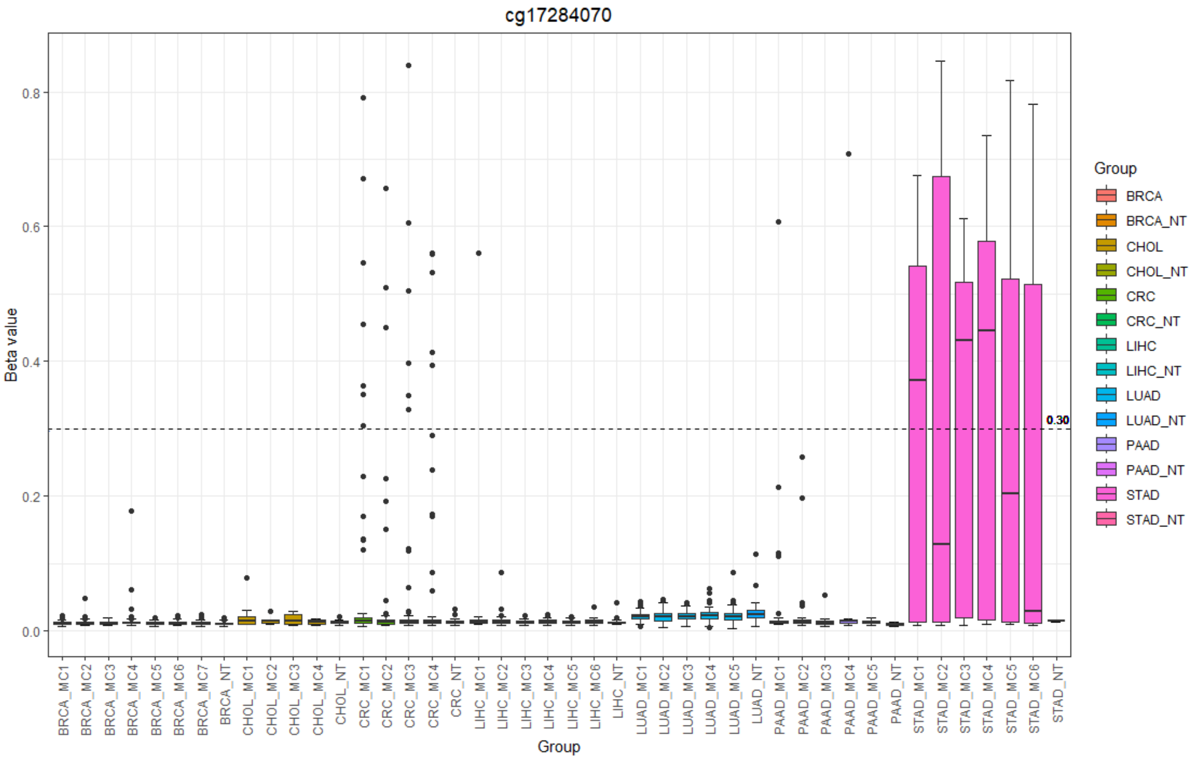
cg17284070
Beta value
0.0
0.2
0.4
0.6
0.8
0.30
Group
BRCA_MC1
BRCA_MC2
BRCA_MC3
BRCA_MC4
BRCA_MC5
BRCA_MC6
BRCA_MC7
BRCA_NT
CHOL_MC1
CHOL_MC2
CHOL_MC3
CHOL_MC4
CHOL_NT
CRC_MC1
CRC_MC2
CRC_MC3
CRC_MC4
CRC_NT
LIHC_MC1
LIHC_MC2
LIHC_MC3
LIHC_MC4
LIHC_MC5
LIHC_MC6
LIHC_NT
LUAD_MC1
LUAD_MC2
LUAD_MC3
LUAD_MC4
LUAD_MC5
LUAD_NT
PAAD_MC1
PAAD_MC2
PAAD_MC3
PAAD_MC4
PAAD_MC5
PAAD_NT
STAD_MC1
STAD_MC2
STAD_MC3
STAD_MC4
STAD_MC5
STAD_MC6
STAD_NT
Group
BRCA
BRCA_NT
CHOL
CHOL_NT
CRC
CRC_NT
LIHC
LIHC_NT
LUAD
LUAD_NT
PAAD
PAAD_NT
STAD
STAD_NT

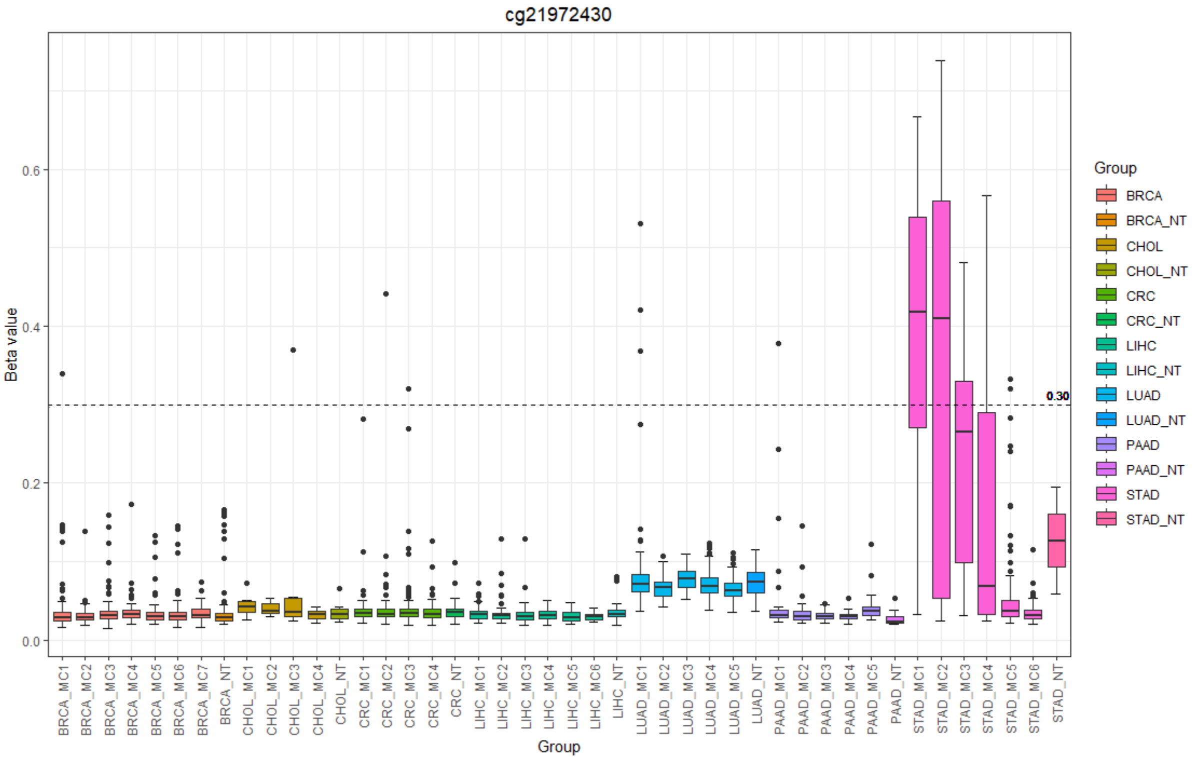
cg21972430
Beta value
0.0
0.2
0.4
0.6
0.30
Group
BRCA_MC1
BRCA_MC2
BRCA_MC3
BRCA_MC4
BRCA_MC5
BRCA_MC6
BRCA_MC7
BRCA_NT
CHOL_MC1
CHOL_MC2
CHOL_MC3
CHOL_MC4
CHOL_NT
CRC_MC1
CRC_MC2
CRC_MC3
CRC_MC4
CRC_NT
LIHC_MC1
LIHC_MC2
LIHC_MC3
LIHC_MC4
LIHC_MC5
LIHC_MC6
LIHC_NT
LUAD_MC1
LUAD_MC2
LUAD_MC3
LUAD_MC4
LUAD_MC5
LUAD_NT
PAAD_MC1
PAAD_MC2
PAAD_MC3
PAAD_MC4
PAAD_MC5
PAAD_NT
STAD_MC1
STAD_MC2
STAD_MC3
STAD_MC4
STAD_MC5
STAD_MC6
STAD_NT
Group
BRCA
BRCA_NT
CHOL
CHOL_NT
CRC
CRC_NT
LIHC
LIHC_NT
LUAD
LUAD_NT
PAAD
PAAD_NT
STAD
STAD_NT

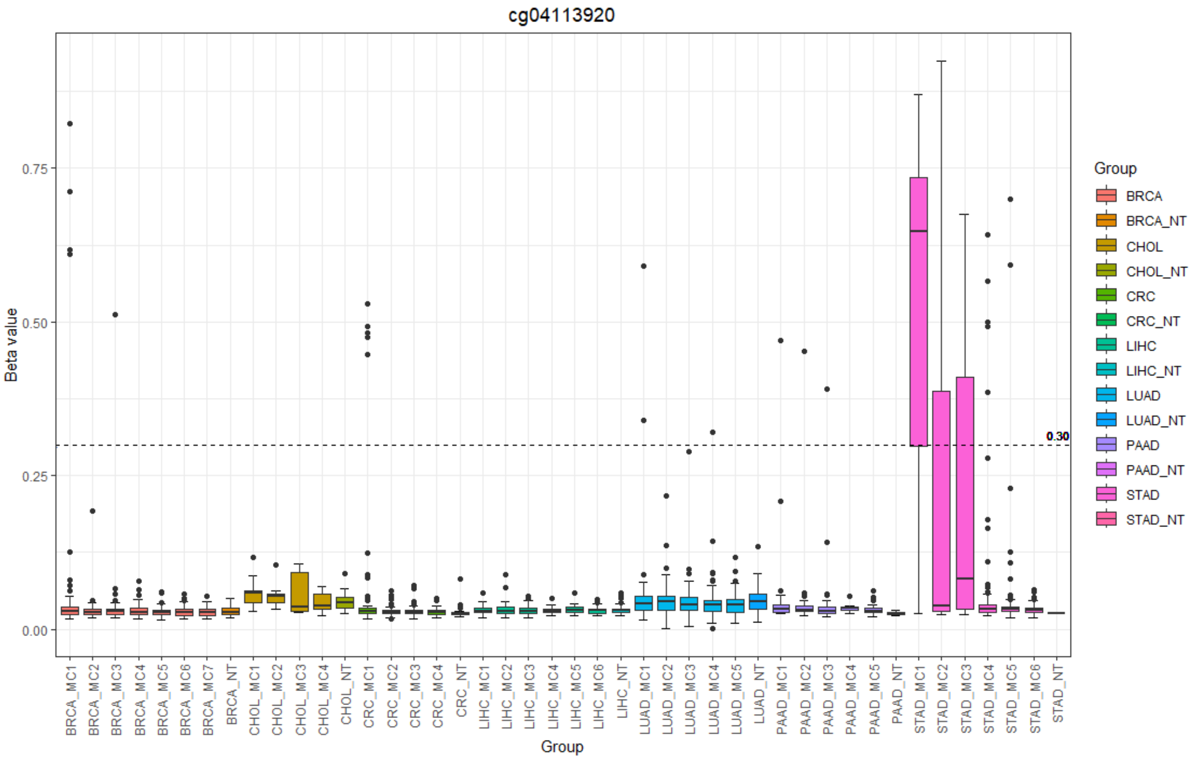
cg04113920
Beta value
0.00
0.25
0.50
0.75
0.30
Group
BRCA_MC1
BRCA_MC2
BRCA_MC3
BRCA_MC4
BRCA_MC5
BRCA_MC6
BRCA_MC7
BRCA_NT
CHOL_MC1
CHOL_MC2
CHOL_MC3
CHOL_MC4
CHOL_NT
CRC_MC1
CRC_MC2
CRC_MC3
CRC_MC4
CRC_NT
LIHC_MC1
LIHC_MC2
LIHC_MC3
LIHC_MC4
LIHC_MC5
LIHC_MC6
LIHC_NT
LUAD_MC1
LUAD_MC2
LUAD_MC3
LUAD_MC4
LUAD_MC5
LUAD_NT
PAAD_MC1
PAAD_MC2
PAAD_MC3
PAAD_MC4
PAAD_MC5
PAAD_NT
STAD_MC1
STAD_MC2
STAD_MC3
STAD_MC4
STAD_MC5
STAD_MC6
STAD_NT
Group
BRCA
BRCA_NT
CHOL
CHOL_NT
CRC
CRC_NT
LIHC
LIHC_NT
LUAD
LUAD_NT
PAAD
PAAD_NT
STAD
STAD_NT

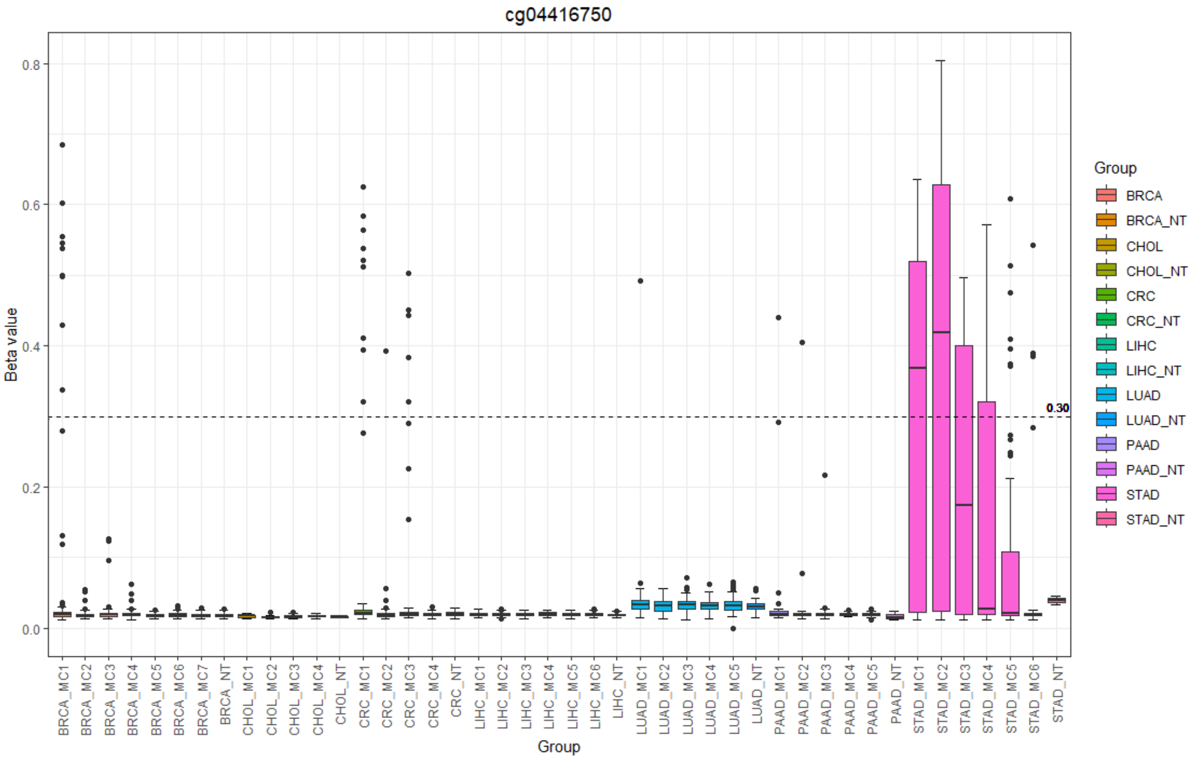
cg04416750
Beta value
0.0
0.2
0.4
0.6
0.8
0.30
Group
BRCA_MC1
BRCA_MC2
BRCA_MC3
BRCA_MC4
BRCA_MC5
BRCA_MC6
BRCA_MC7
BRCA_NT
CHOL_MC1
CHOL_MC2
CHOL_MC3
CHOL_MC4
CHOL_NT
CRC_MC1
CRC_MC2
CRC_MC3
CRC_MC4
CRC_NT
LIHC_MC1
LIHC_MC2
LIHC_MC3
LIHC_MC4
LIHC_MC5
LIHC_MC6
LIHC_NT
LUAD_MC1
LUAD_MC2
LUAD_MC3
LUAD_MC4
LUAD_MC5
LUAD_NT
PAAD_MC1
PAAD_MC2
PAAD_MC3
PAAD_MC4
PAAD_MC5
PAAD_NT
STAD_MC1
STAD_MC2
STAD_MC3
STAD_MC4
STAD_MC5
STAD_MC6
STAD_NT
Group
BRCA
BRCA_NT
CHOL
CHOL_NT
CRC
CRC_NT
LIHC
LIHC_NT
LUAD
LUAD_NT
PAAD
PAAD_NT
STAD
STAD_NT

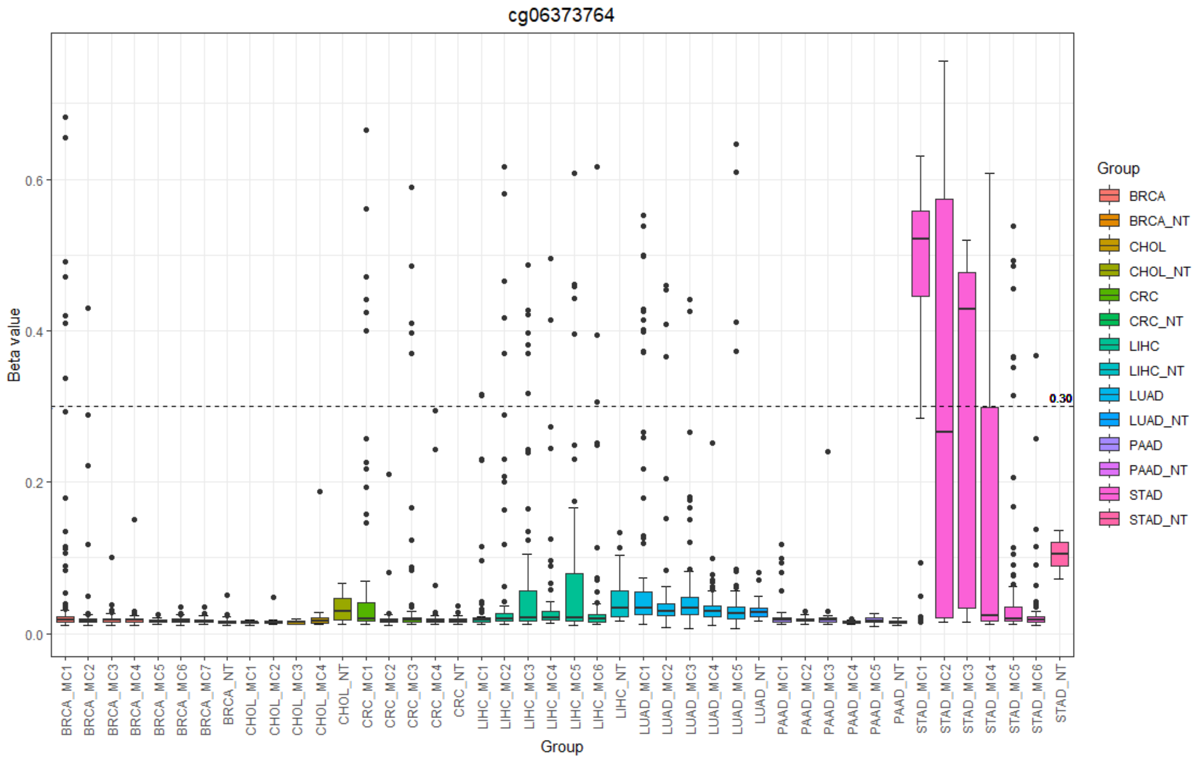
cg06373764
Beta value
Group
0.30
BRCA_MC1 BRCA_MC2 BRCA_MC3 BRCA_MC4 BRCA_MC5 BRCA_MC6 BRCA_MC7 BRCA_NT CHOL_MC1 CHOL_MC2 CHOL_MC3 CHOL_MC4 CHOL_NT CRC_MC1 CRC_MC2 CRC_MC3 CRC_MC4 CRC_NT LIHC_MC1 LIHC_MC2 LIHC_MC3 LIHC_MC4 LIHC_MC5 LIHC_MC6 LIHC_NT LUAD_MC1 LUAD_MC2 LUAD_MC3 LUAD_MC4 LUAD_MC5 LUAD_NT PAAD_MC1 PAAD_MC2 PAAD_MC3 PAAD_MC4 PAAD_MC5 PAAD_NT STAD_MC1 STAD_MC2 STAD_MC3 STAD_MC4 STAD_MC5 STAD_MC6 STAD_NT
Group
BRCA
BRCA_NT
CHOL
CHOL_NT
CRC
CRC_NT
LIHC
LIHC_NT
LUAD
LUAD_NT
PAAD
PAAD_NT
STAD
STAD_NT

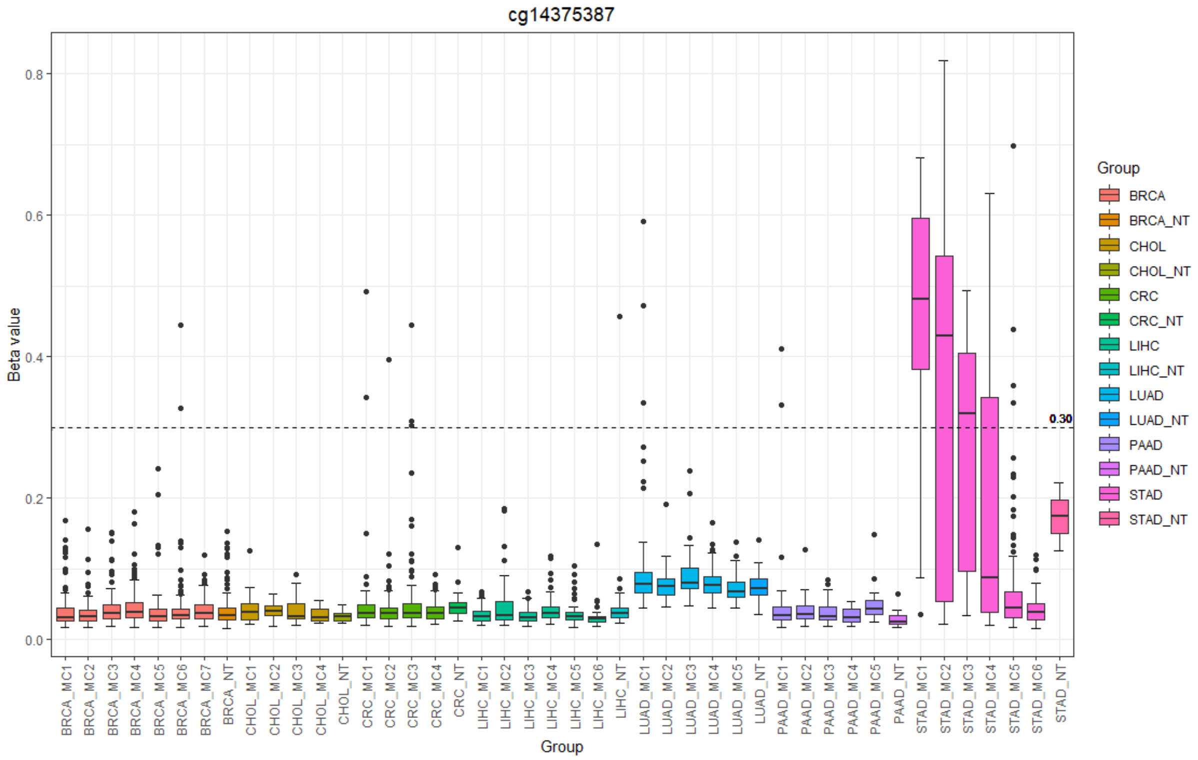
cg14375387
Beta value
Group
0.30
BRCA_MC1 BRCA_MC2 BRCA_MC3 BRCA_MC4 BRCA_MC5 BRCA_MC6 BRCA_MC7 BRCA_NT CHOL_MC1 CHOL_MC2 CHOL_MC3 CHOL_MC4 CHOL_NT CRC_MC1 CRC_MC2 CRC_MC3 CRC_MC4 CRC_NT LIHC_MC1 LIHC_MC2 LIHC_MC3 LIHC_MC4 LIHC_MC5 LIHC_MC6 LIHC_NT LUAD_MC1 LUAD_MC2 LUAD_MC3 LUAD_MC4 LUAD_MC5 LUAD_NT PAAD_MC1 PAAD_MC2 PAAD_MC3 PAAD_MC4 PAAD_MC5 PAAD_NT STAD_MC1 STAD_MC2 STAD_MC3 STAD_MC4 STAD_MC5 STAD_MC6 STAD_NT
Group
BRCA
BRCA_NT
CHOL
CHOL_NT
CRC
CRC_NT
LIHC
LIHC_NT
LUAD
LUAD_NT
PAAD
PAAD_NT
STAD
STAD_NT

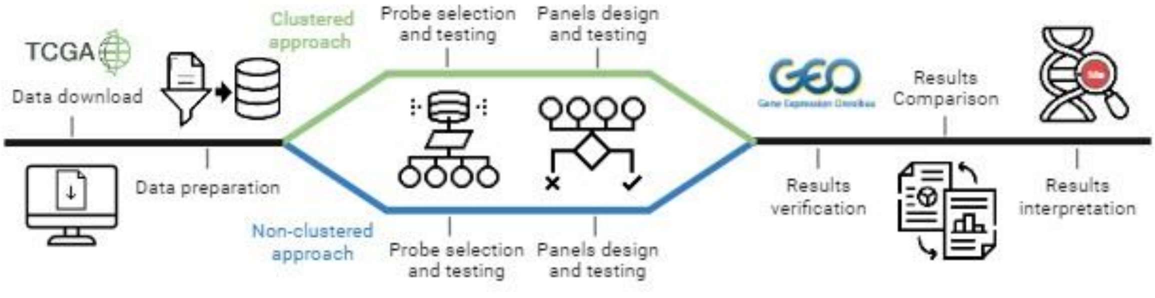

Fig. S4: Simple study workflow. After downloading and preparation, the TCGA dataset were analyzed using two different approaches: a non-clustered approach (no clustering was performed) and a clustered approach (unsupervised clustering was performed within each project). Separate results were obtained for each of these approaches (probe candidates were selected and tested, followed by a panel design and testing for each cancer type). The results were verified by an independent GEO dataset. At the end, results from both approaches were compared and interpreted.

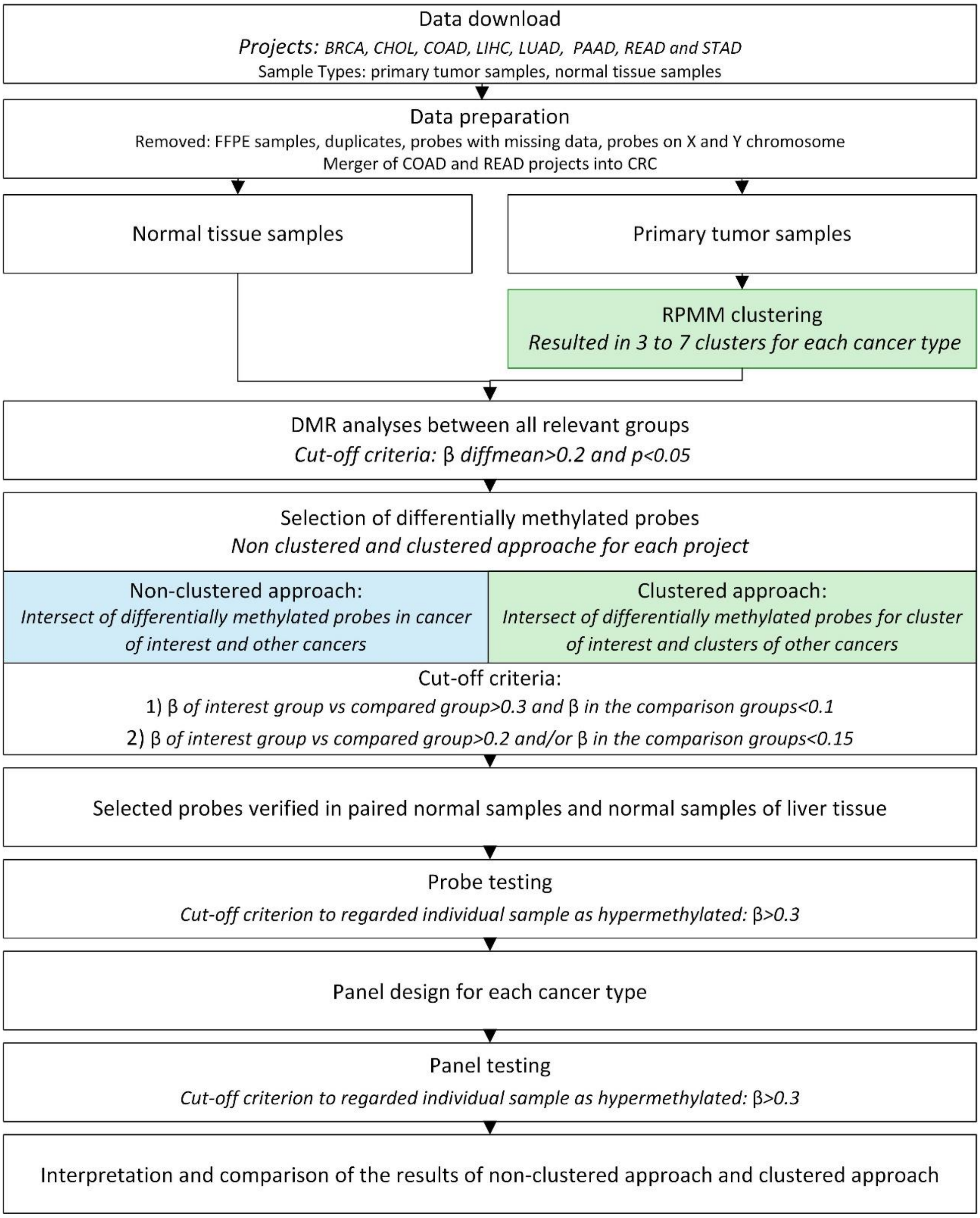

Fig. S5: Detailed workflow showing the probe selection and panel design steps for the non-clustered and clustered approaches. After data download, data preparation and clustering of primary tumor samples in a clustered approach, differentially methylated regions (DMR) analyses were performed. Candidate probes that were differentially methylated in different cancer types were selected and tested in normal tissue samples. After testing the candidate probes, panels were designed and tested for each cancer type. The blue colored steps were used only for the non-clustered approach and the green colored steps were used only for the clustered approach. The white colored steps were used in both approaches.
